# A multi-enzyme machine polymerizes the *Haemophilus influenzae* type b capsule

**DOI:** 10.1038/s41589-023-01324-3

**Published:** 2023-06-05

**Authors:** Javier O. Cifuente, Julia Schulze, Andrea Bethe, Valerio Di Domenico, Christa Litschko, Insa Budde, Lukas Eidenberger, Hauke Thiesler, Isabel Ramón Roth, Monika Berger, Heike Claus, Cecilia D’Angelo, Alberto Marina, Rita Gerardy-Schahn, Mario Schubert, Marcelo E. Guerin, Timm Fiebig

**Affiliations:** 1 Structural Glycobiology Laboratory, Biocruces Bizkaia Health Research Institute, Cruces University Hospital, Barakaldo, Spain; 2 Structural Glycobiology Laboratory, Center for Cooperative Research in Biosciences (CIC bioGUNE), Basque Research and Technology Alliance (BRTA), Bizkaia Technology Park, Derio, Spain; 3 Institute of Clinical Biochemistry, Hannover Medical School, Hannover, Germany; 4 Department of Applied Genetics and Cell Biology, University of Natural Resources and Life Sciences, Vienna, Austria; 5 Institute for Hygiene and Microbiology, University of Würzburg, Würzburg, Germany; 6 Department of Biosciences and Medical Biology, University of Salzburg, Salzburg, Austria; 7 Ikerbasque Basque Foundation for Science, Bilbao, Spain

**Keywords:** Carbohydrates, Glycobiology, Enzyme mechanisms, X-ray crystallography, Vaccines

## Abstract

Bacterial capsules have critical roles in host-pathogen interactions. They provide a protective envelope against host recognition, leading to immune evasion and bacterial survival. Here we define the capsule biosynthesis pathway of *Haemophilus influenzae* serotype b (Hib), a Gram-negative bacterium that causes severe infections in infants and children. Reconstitution of this pathway enabled the fermentation-free production of Hib vaccine antigens starting from widely available precursors and detailed characterization of the enzymatic machinery. The X-ray crystal structure of the capsule polymerase Bcs3 reveals a multi-enzyme machine adopting a basket-like shape that creates a protected environment for the synthesis of the complex Hib polymer. This architecture is commonly exploited for surface glycan synthesis by both Gram-negative and Gram-positive pathogens. Supported by biochemical studies and comprehensive 2D nuclear magnetic resonance, our data explain how the ribofuranosyltransferase CriT, the phosphatase CrpP, the ribitol-phosphate transferase CroT and a polymer-binding domain function as a unique multi-enzyme assembly.

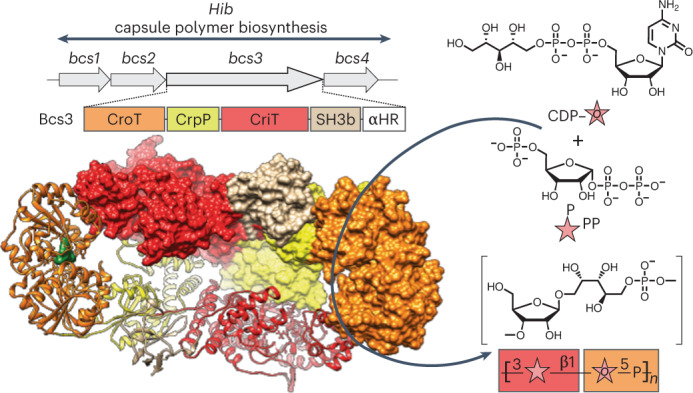

## Abstract (web summary)

Polymerase Bcs3, which allows the fermentation-free synthesis of *Haemophilus influenzae* type b capsule for vaccine development, adopts a basket-like shape with all six active sites facing the interior, creating a protected environment for catalysis.

## Main

**Fig. 1:**
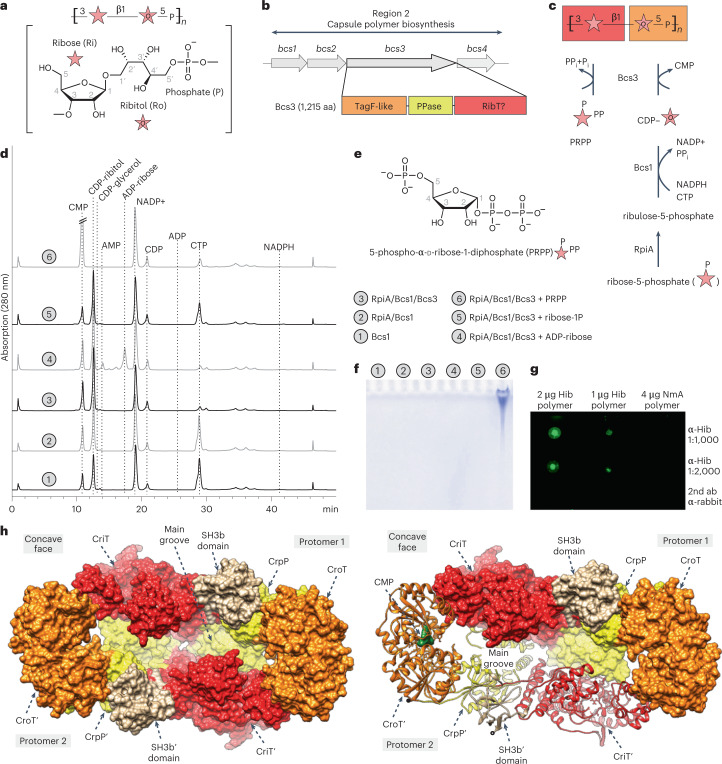
Bcs3 is the capsule polymerase of Hib. **a**, Chemical structure and schematic representation of the Hib capsule polymer repeating unit. **b**, Genetic organization of region 2 of the capsule gene cluster of Hib. Schematic showing the following three predicted regions of the Bcs3 amino acid sequence: an N-terminal TagF-like protein, a central phosphatase protein and a C-terminal region containing a putative ribosyltransferase (RibT) protein. **c**, Schematic of an enzyme cascade for the synthesis of Hib polymer. Ribose-5-phosphate is converted by the d-ribose-5-phosphate isomerase RpiA into ribulose-5-phosphate, which is reduced (using NADPH) and activated (using CTP) by Bcs1 to yield CDP-ribitol, the donor substrate for the ribitol-5-phosphate transfer catalyzed by Bcs3. PRPP is the donor substrate for the ribose transfer catalyzed by Bcs3. Red and orange background colors refer to the moieties that are most likely transferred by the domains shown in **b**. **d**, HPLC-based AEC for the analysis of enzyme reactions as indicated by numbers in gray circles. The CMP peak in chromatogram 6 was cropped to harmonize the overall appearance of the figure. CDP-glycerol was used as a control for the commercially unavailable CDP-ribitol. **e**, Chemical structure of PRPP. **f**, Alcian blue-stained polyacrylamide gel corresponding to **d**. **g**, Dot blot analysis used a Hib-specific agglutination serum for detection. Phosphate-containing polymer from *Neisseria meningitidis* serogroup A^50^ was used as negative control. **h**, The overall structure of the Bcs3 dimer in complex with CMP, with both protomers shown in surface representation (left) and one protomer shown in cartoon representation (right) to visualize the secondary structural organization and the CMP (green). Each protomer of the homodimer is composed of (1) the ribofuranosyltransferase CriT (red), (2) the phosphatase CrpP (yellow), (3) the ribitol-phosphate transferase CroT (orange) and (4) an SH3b domain (tan). See also Supplementary Fig. 5. Source data

*Haemophilus influenzae* is a Gram-negative bacterium colonizing the human nasopharynx. It commonly causes upper and lower respiratory infections and occasionally more serious diseases like meningitis and septicemia with high incidence rates, especially in infants^1^. Based on the chemical properties of the linear, charged capsule polymer surrounding the pathogen, *H.* *influenzae* can be grouped into six serotypes a–f, of which serotype b (Hib) is clinically most important. The capsule polymer of Hib, consisting of a ribofuranose- (Rib*f*) and ribitol-5-phosphate-containing repeating unit^2^ ([→3)-β-d-Rib*f*-(1→1)-d-Ribitol-(5→OPO_3_→]_*n*_) (Fig. 1a), is one of the pathogen’s main virulence factors and enables it to evade the host’s immune system^1,3^.

Capsule polymers can be used as an antigen in vaccine formulations^4^. In the early 1990s, the Hib conjugate vaccine was the first glycoconjugate vaccine ever licensed and was immensely successful in reducing the disease burden^5^. However, the global distribution of Hib conjugate vaccines is still a challenge, partly because the high production costs associated with purifying the capsule polymer from pathogen cultures limit the introduction of Hib vaccination worldwide, including in developing countries^6^. Promising alternative strategies are the use of either chemical and/or enzymatic synthesis for the provision of capsule polymer^7^. Notably, a chemically synthesized, cost-effective Hib vaccine was introduced in the Cuban national immunization schedule in 2004 (ref. ^5^). However, an enzyme-based synthesis approach has not yet been realized, as the enzymatic machinery responsible for capsule biosynthesis in Hib remains elusive. Its characterization would open up exciting opportunities for the fermentation-free production of a Hib vaccine from ultrapure components, both in the context of recombinant proteins and substrates.

**Extended Data Fig. 1:**
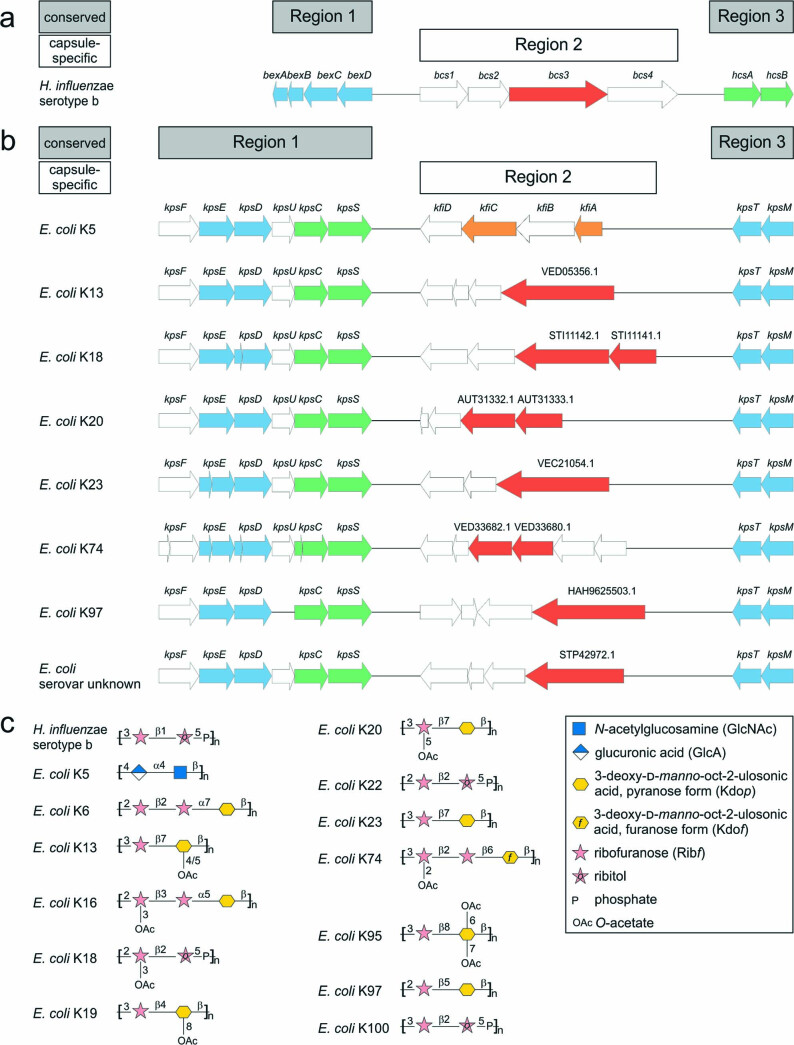
Schematic overview of capsule gene clusters and capsule structures from Hib and *E. coli*. They encode ABC transporter-dependent capsule assembly systems (group 2 for *E. coli* nomenclature) that can be found in other important human and animal pathogens, like extraintestinal pathogenic *E. coli*, *Neisseria meningitidis*, *Actinobacillus pleuropneumoniae*, *Campylobacter jejuni*, and *Pasteurella multocida*^3^. **a, b**, Capsule gene clusters of (**a**) Hib and (**b**) *E. coli* strains are divided in conserved regions (gray boxes) and capsule-specific regions (white boxes), encoding proteins involved in the generation of a conserved glycolipid^31^ (green), proteins involved in polymer export (blue), and proteins involved in capsule polymer synthesis. The *E. coli* K5 capsule gene cluster is depicted as a reference for a well-characterized capsule biosynthesis system^3^ and the genes encoding the polymerase domains, which share no similarity with Bcs3, are indicated in orange. The *bcs3* gene and genes harboring homologs of the CrpP and CriT domains are indicated in red. In *E. coli*, these genes are either paired with genes encoding CroT homologs in strains expressing capsules containing ribitol-phosphate (for example in K18). Or they are paired with proteins of unknown function, for which Phyre2^13^ homology modeling predicts (among others) weak similarity to Kdo-transferases. In both cases, the domains can be part of a single polypeptide or expressed separately. **c**, Capsule structures of Hib and *E. coli* strains as indicated in **a, b**, and capsule structures of *E. coli* strains that harbor ribofuranose in their capsule polymer, but for which sequence information is unavailable (extracted from the *E*. *coli* K antigen 3-dimensional structure database EK3D)^11^.

On the DNA level, capsule expression in Hib is organized in a conserved region named capsule gene cluster, which can be subdivided into regions 1–3 (refs. ^8,9^; Fig. 1b and Extended Data Fig. 1). Regions 1 and 3 are conserved between species and encode the export machinery belonging to an ABC-transporter-dependent capsule assembly system, historically referred to as group 2 biosynthesis system^3^. Region 2 is serotype-specific and encodes the capsule biosynthesis enzymes. In Hib, region 2 contains four open reading frames called *bcs1*-*bcs4* (ref. ^8^; Fig. 1b and Extended Data Fig. 1a), of which only the product of *bcs1* (Bcs1) was biochemically characterized^10^ and shown to encode a CDP-ribitol synthase, which putatively provides the nucleotide-activated precursor for the ribitol-5-phosphate moiety in the Hib polymer (Fig. 1c). So far, no information is available about the function of the gene products Bcs2–4.

At least the following two enzymatic functions are needed to assemble the Hib polymer ([→3)-β-d-Rib*f*-(1→1)-d-Ribitol-(5→OPO_3_→]_*n*_): a ribofuranosyltransferase and a ribitol-phosphate transferase. Despite the abundance of ribose in bacterial surface glycans^11^, the enzymatic machinery for ribose incorporation into capsular polysaccharides has not been identified yet. Ribitol-phosphate transferases have been described biochemically in the context of Gram-positive wall teichoic acids (WTAs)^12^. However, evidence of these enzymes in the Gram-negative background is lacking, and structural information is missing entirely. In this context, the capsule biosynthesis machinery of Hib might connect biosynthesis modules used in both Gram-negative and Gram-positive pathogens, and the structural characterization of the enzyme would greatly improve the understanding of surface glycan biosynthesis in both systems, thereby advancing the search for the discovery of new antimicrobials.

In this study, we defined the entire pathway for the fermentation-free synthesis of the Hib capsule polymer starting from purified enzymes and inexpensive, widely available substrates. We identified Bcs3 as the capsule polymerase of Hib and solved its crystal structure in complex with cytidine monophosphate (CMP), phosphate and Hib polymer. The enzymes of Bcs3 form a machine for heteropolymer synthesis of as yet undescribed complexity. We unveil the molecular basis of substrate specificity in each one of the three enzymes and suggest a mechanism by which they act in concert to sequentially assemble the Hib capsule.

## Results

### Bcs3 is the Hib capsule polymerase

From the noncharacterized genes of the Hib capsule gene cluster (*bcs2*–*4*), only *bcs3* codes for an enzyme large enough (1,215 amino acids) to encompass at least two catalytic activities (Fig. 1b). Homology modeling performed with Bcs3 using Phyre2 (ref. ^13^) predicted (Fig. 1b) (1) an N-terminal TagF-like polymerase^14,15^ (Supplementary Fig. 1), (2) a central phosphatase^16^ and (3) a large C-terminal region of ca. 600 amino acids for which no significant homologs could be provided by Phyre2. We hypothesized that this region might harbor the ribosyltransferase domain. Bcs3 was cloned and purified, and CDP-ribitol was considered a suitable donor substrate. Because CDP-ribitol is not commercially available and its precursor ribulose-5-phosphate is comparatively expensive, the enzymes Bcs1 (ref. ^10^) and RpiA^17^ from Hib were cloned to establish a cascade synthesis for CDP-ribitol starting from d-ribose-5-phosphate, NADPH and CTP (Fig. 1c,d and Supplementary Fig. 2). As expected, the presence of Bcs1 in a reaction containing ribulose-5-phosphate led to the consumption of NADPH, while NADP^+^ and a peak eluting similarly to CDP-glycerol were produced. The same result can be observed if ribulose-5-phosphate is produced in situ from ribose-5-phosphate by RpiA (Fig. 1d, compare reactions 1 and 2), indicating that CDP-ribitol can be generated by RpiA and Bcs1 in a one-pot reaction.

### PRPP is the ribose donor for the synthesis of Hib polymer

**Extended Data Fig. 2:**
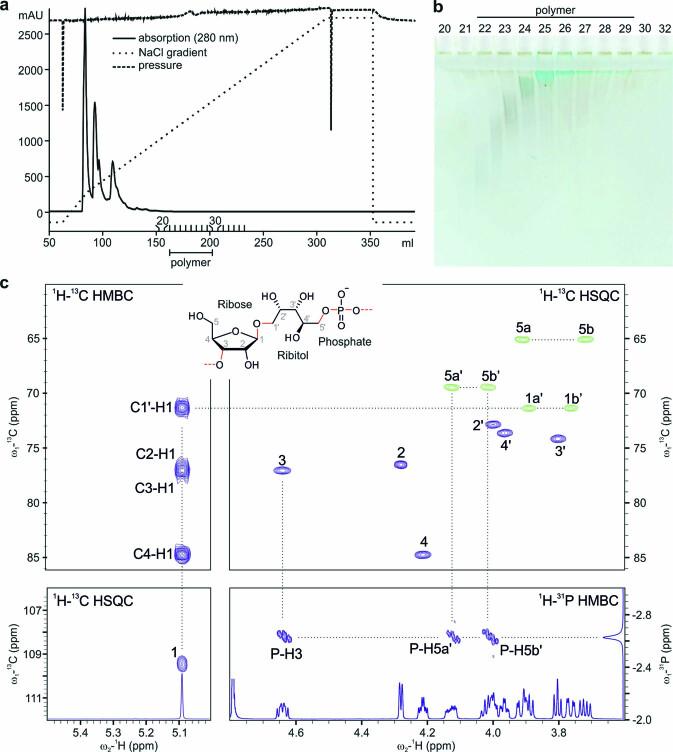
Purification and NMR analysis of enzymatically synthesized polymer. **a**, Hib polymer (5 mg) was purified from an enzyme reaction containing RpiA, Bcs1, and Bcs3 using preparative AEC. The remaining nucleotide substrates and nucleotide products (see Fig. 1c) were UV-detectable at 280 nm, eluted early from the column and could be separated from **b**, the UV-inactive Hib polymer, which was visualized by Alcian blue / silver staining after PAGE. Polymer containing fractions were pooled, dialyzed against water, and **c**, analyzed by 1D and 2D NMR. All correlations are in agreement with published literature (Supplementary Table 1). Correlations confirming the linkage of the moieties in the repeating unit are highlighted in red in the structure and indicated by dashed lines. HMBC: heteronuclear multiple bond correlation, HSQC: heteronuclear single-quantum coherence. Source data

ADP-ribose, α-d-ribose-1-phosphate (Rib-1P) and 5-phospho-α-d-ribose-1-diphosphate (PRPP; Fig. 1e) have been discussed as putative ribose donors for Hib polymer synthesis^9^ and were thus tested as substrate candidates for the putative ribofuranosyltransferase activity of Bcs3. Because CDP-ribitol degraded during attempts of isolation (Supplementary Fig. 3), it was generated in situ by Bcs1 and RpiA. Interestingly, CDP-ribitol was only used up in the presence of PRPP, with concomitant production of CMP and large polymeric products detectable by Alcian blue-stained PA gel^18^ (Fig. 1d,f, reaction 6). In contrast, ADP-ribose and ribose-1-phosphate did not stimulate the consumption of CDP-ribitol nor the production of CMP or polymer (Fig. 1d,f, reactions 4 and 5). Thus, we concluded that PRPP is the donor substrate for Bcs3. To confirm the identity of the produced polymer, the enzyme cascade of RpiA, Bcs1 and Bcs3 was scaled up. Polymer was purified and analyzed by immunoblotting (Fig. 1g) and nuclear magnetic resonance (NMR; Extended Data Fig. 2). ^1^H and ^13^C chemical shifts agreed perfectly with spectra previously reported for natural Hib polymer (Supplementary Tables 1 and 2). Notably, 1D ^31^P NMR and ^1^H-^31^P HMBC spectra showed only a single phosphodiester signal and no indication of the presence of phosphomonoesters in the polymer, suggesting that the predicted phosphatase (Fig. 1b) might be required to remove the 5-phosphate present in PRPP. Of note, the ribofuranose has α-configuration in PRPP and β-configuration in the polymer (Fig. 1a,e), indicating that Bcs3 uses an inverting mechanism.

### The overall architecture of Bcs3

**Extended Data Fig. 3:**
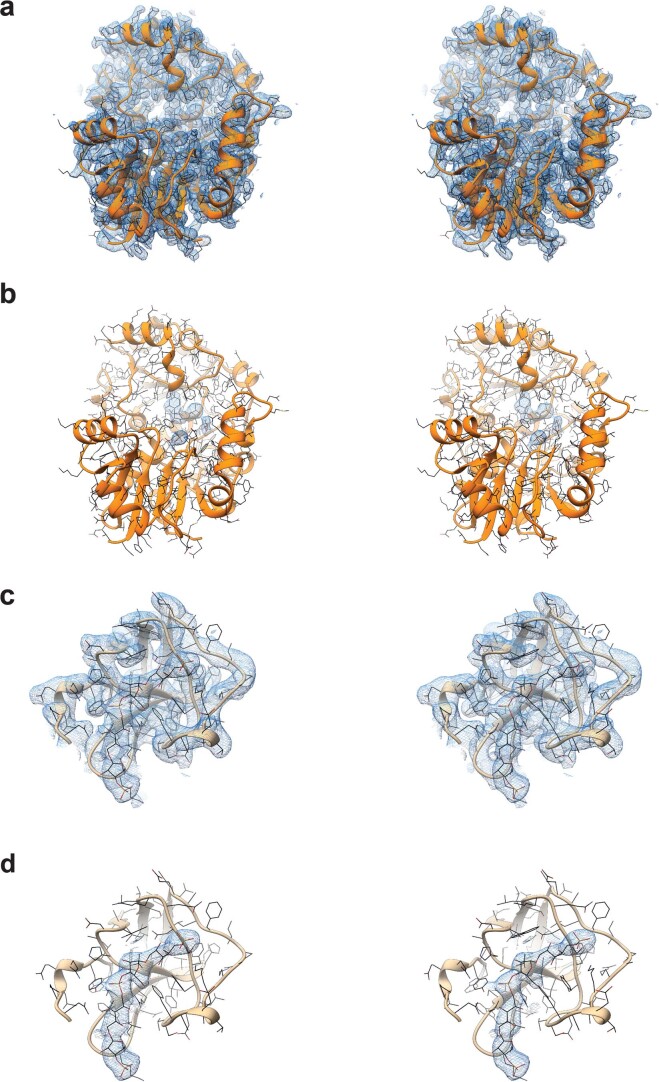
Local stereo views of CroT in complex with CMP, and of the SH3b domain in complex with polymer. **a**, Model of CroT (orange ribbons) in complex with CMP, glycerol, and phosphate and the corresponding electron density map displayed at 1 RMSD. **b**, Local density map for the CMP, glycerol, and phosphate found in CroT. **c**, Model of selected SH3b domain (tan-colored ribbons) in complex with DP2 (dimer of Hib capsule polymer) displaying the overall density map at 1 RMSD. **d**, Density map for the DP2 ligand. The density for ribose, ribitol, and phosphate allows the unambiguous placing of the DP2 and helped determine the orientation of the polymer.

**Extended Data Fig. 4:**
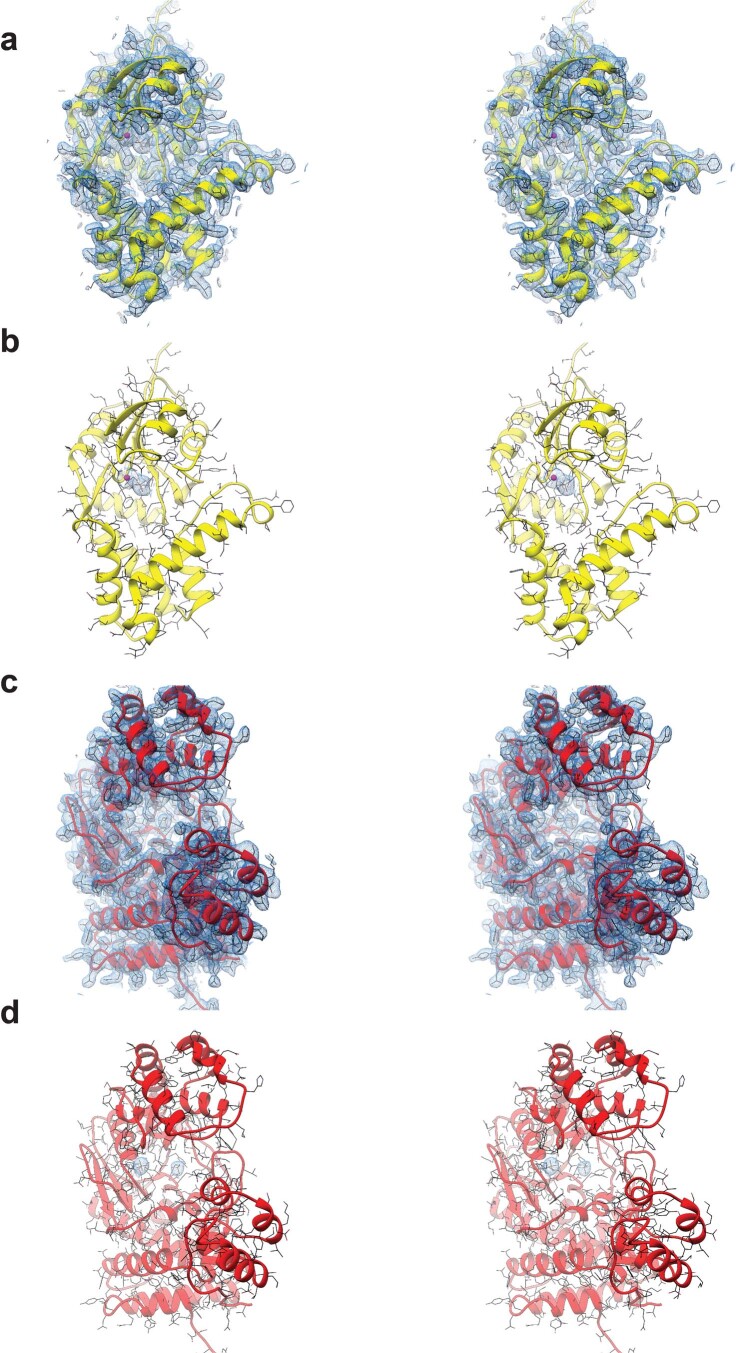
Local stereo views of the CrpP enzyme and CriT in complex with CMP. **a**, Model of selected CrpP (yellow ribbons) and overall density map at 1 RMSD. **b**, The density map for the phosphate and Zn^2+^ found in the active site of CrpP was used as a reference for the polymer substrate docking. **c**, Model of selected CriT (red ribbons) displaying the overall density map at 1 RMSD. CriT has an overall size of 30 Å × 25 Å × 25 Å. The overall scaffold and the conformation of the CriT and CriT’ protomers were essentially preserved (RMSD of 0.77 Å for residues 592–1036). **d**, The density map for the two phosphates found in the active site was used as a reference for the docking of PRPP.

Crystallographic studies were performed using a C-terminally truncated version of the enzyme (Supplementary Fig. 4). The crystal structure of Bcs3 (residues 1–1,115, called Bcs3-CMP hereafter) in complex with CMP was solved by molecular replacement methods using an Alphafold^19^ model (see Methods for details) at a maximum resolution of 2.9 Å (Fig. 1h, Supplementary Fig. 5 and Supplementary Table 3). Bcs3-CMP crystallized in the *P* 2_1_2_1_2_1_ space group, with two molecules in the asymmetric unit and residues 1–1,115 visible in the electron density map (Extended Data Figs. 3 and 4 and Supplementary Fig. 6). The following five different regions were identified from the N- to the C-terminus (Fig. 1h and Supplementary Fig. 5): (1) a TagF-like capsule polymerase^14,15^ called CroT (**C**apsule **r**ibit**o**l-5-phosphate **T**ransferase (residues 1–373), (2) a phosphatase called CrpP (**C**apsule **r**ibose **p**hos**P**hatase (residues 374–591), (3) the ribose-5-phosphate transferase CriT (**C**apsule **ri**bose-5-phosphate **T**ransferase (residues 592–1,036) and (4) an SH3b^20^ domain (residues 1,037–1,115). Region 5, the amino acids C-terminally attached to the SH3b domain, was not visible in the structure (residues 1,116–1,215; Supplementary Fig. 7). However, a bundle of amphipathic α-helices (CT-HB) compatible with membrane association and/or protein–protein interactions could be predicted for this region using Alphafold^19^. Interestingly, these helices are not present in Bcs3 homologs identified via BLAST^21^ in Gram-positive bacteria (Supplementary Data 1), suggesting that they might be specific for the Gram-negative capsule biosynthesis systems^3^ shown in Extended Data Fig. 1.

**Extended Data Fig. 5:**
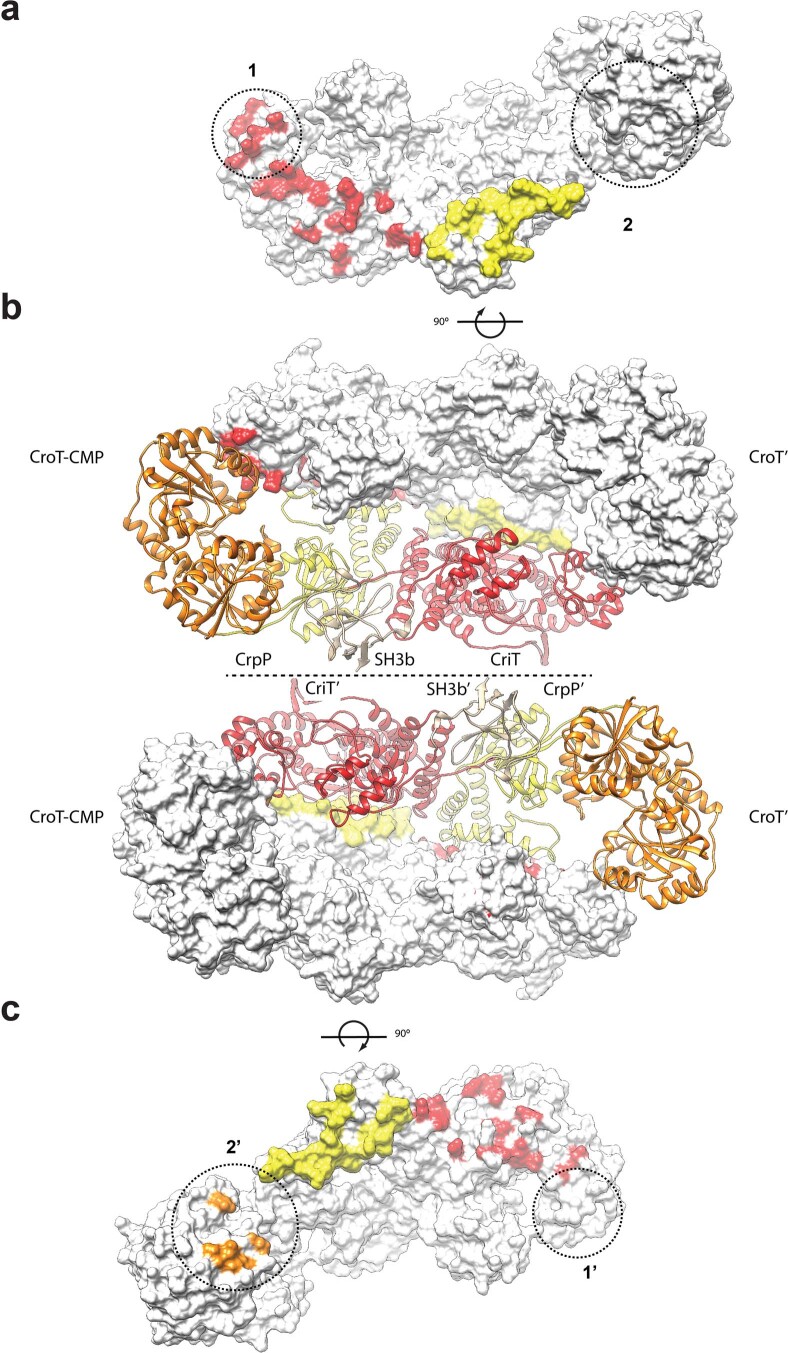
Bcs3 protomer dimerization contacts. **a-c**, Contacts and ribbon representations are colored as follows: CroT (orange), CrpP (yellow), CriT (red), and SH3b domain (tan). In panel **b**, the dimer from the Bcs3-CMP complex is shown in two representations. *Top*: The protomer containing the CroT-CMP complex is shown in ribbons and the complementary protomer in a surface representation (up to the dashed line). *Bottom*: The protomer containing the CroT-CMP complex is shown in a surface representation and the complementary protomer is shown in ribbons (below the dashed line). The panels **a** and **c** present 90° rotations of the individual protomers in surface representation, disclosing the surface contacts between protomers by coloring contacting residues according to the enzyme they belong to. Note the conservation of the main interactions in the center of the protomers that correspond to interactions between CrpP and CriT’, and CrpP’ and CriT. The HB-1 of CriT mediates the interaction of CriT with the phosphatase CrpP and the SH3b domain of the same protomer, as well as with the ribose transferase CriT’ of the other protomer (see HB-1 in Fig. 2a-c). The HB-2 mediates the interaction of CriT with (i) CrpP’ and (ii) the C-terminal Rossmann-fold domain of CroT’ from the neighboring protomer. In contrast, HB-3 is mainly exposed to the solvent. Two encircled regions in the protomers, 1 and 1’ for CriT and CriT’ (**a, c**), and 2 and 2’ for CroT and CroT’ (**a, c**), display variability associated with the conformational changes in the CriT-CroT’ and CriT’-CroT interphase. These changes in the interaction correlate with the presence or absence of CMP in CroT. The contacts visualized above are mediated by the following structural elements (secondary structural elements are shown in Supplementary Data 1): (i) the α42 (residues 756–761) of CriT interacts with α11 (residues 207–210) and α14 (residues 240–248) of CroT, and (ii) α43 (residues 770–780) of CriT interacts with α11 (residues 207–210), the loop connecting β7-α11 (residues 196–206), and α17 (residues 300–303) of CroT. Furthermore, (i) α24 (residues 419–423) of CrpP interacts with the α34-α35 loop (residues 607–612), the α47-α48 loop (residues 923–928), and α48 (residues 929–946) of CriT, (ii) the long α25 (residues 425–443) of CrpP interacts with α39 (residues 722–725), the α39-α40 loop (residues 726–736), β22-α45 loop (residues 818–823), β23-β24 loop (residues 848–857), β24-α46 loop (863–869), and α48 (929–946) of CriT, (iii) the α26-α27 loop (residues 468–470) of CrpP interacts with the end of α36 (residues 638–669) of CriT, and (iv) α26 (residues 456–467) of CrpP interacts with the end of α36 (residues 638–669), β24 (858–862), β23-β24 loop (residues 848–857), and β24-α46 loop (863–869) of CriT.

The Bcs3 protomers build into a physiological and functional head-to-tail dimer (Fig. 1h and Supplementary Fig. 4f,5), which is assembled primarily by interactions between CriT from one protomer and CroT’/CrpP’ from the neighbor protomer, with an overall basket-like shape (Extended Data Fig. 5). The active sites of the three enzymes in each protomer, as well as each of the SH3b domains and the predicted amphipathic α-helices, are located at the concave surface of the dimer (Supplementary Fig. 5). The concave surface of Bcs3 contains several hydrophobic patches interspersed with clusters of positively charged residues, which could indicate potential binding sites for Rib*f*/ribitol and for phosphate of the repeating unit, respectively. In contrast, the opposite side of Bcs3 displays a negatively charged surface, which would generate a substantial electrostatic repulsion with the bacterial plasma membrane (Supplementary Fig. 5d).

### Structural basis of CriT substrate specificity and catalysis

**Fig. 2:**
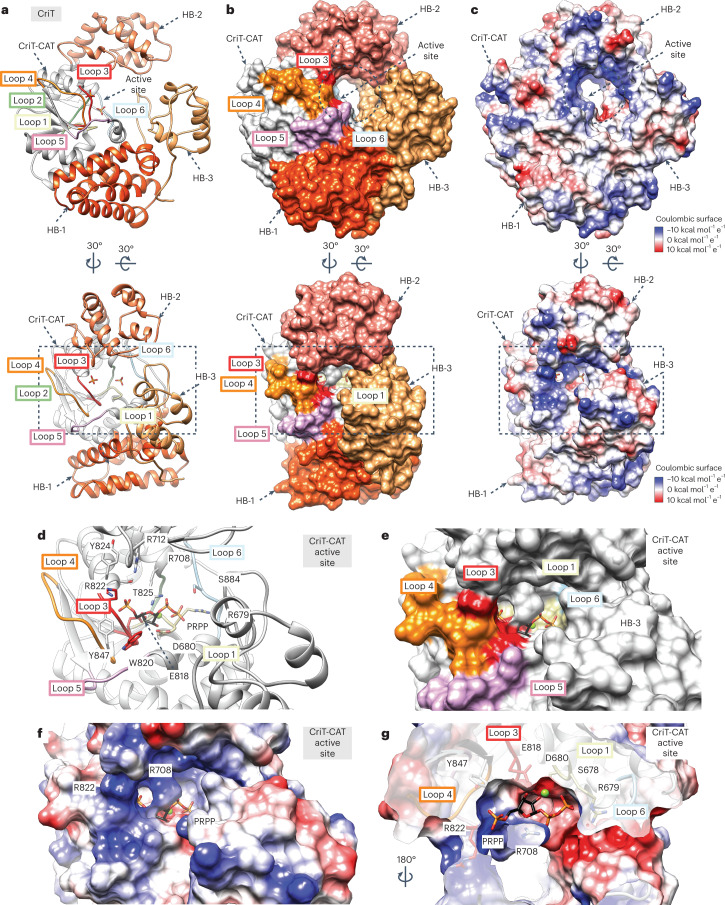
The structure of CriT. **a**, Ribbon representation of a selected CriT from the crystal structure of Bcs3-CMP. Two different orientations show the overall architecture and the central location of the active site. Colored elements highlight HB-1 (orange), HB-2 (pink-orange), HB-3 (gold) and CriT-CAT (gray). Loops participating in the active site are colored as follows: loop 1 (tan, residues 678–680, connecting β20-α37), loop 2 (green, residues 705–707, connecting β21-α38), loop 3 (red, residues 818–823, connecting β22-α45), loop 4 (orange, residues 848–857, connecting β23-β24), loop 5 (pink, residues 863–869, connecting β24-α46) and loop 6 (light blue, residues 880–887, connecting α46-β25). Note the presence of the two phosphates found in the active site. **b**, Corresponding surface representation of CriT using the color code shown in **a** reveals the surface topology and active site. Note that HB-2 and HB-3 generate a donut shape surrounding the active site (dashed circle). **c**, Corresponding Coulombic charge surface representation reveals an intense positive character at the active site entrance. **d**–**f**, Detail of the active site in ribbon (**d**) and surface representation (**e**, **f**) showing the docked PRPP and Mg^2+^. Relevant active site residues participating in substrate binding are indicated. Specifically, the side chains of R708, R712 and T825, and main chains of residues 821–825 (part of loop 3, red, (**d**)) coordinate the PRPP 5-phosphate group, whereas the side chains of S678, S707, S884 and R679 form the pocket that accounts for the interaction with the β-phosphate of the pyrophosphate moiety. Note that the side chains of E818 and D680 accommodate the ribose moiety, while positively charged residues surrounding the binding site hold the phosphate moieties in place. **g**, A cross-section reveals the PRPP-Mg^2+^ binding site cavity, where R708 appears to be involved in positioning the ribose ring.

CriT comprises the following four domains from the N- to the C-terminus (Fig. 2a–c): (1) a first α-helical bundle (HB-1; residues 599–654 and 904–959), (2) a catalytic domain (CriT-CAT; residues 655–715 and 796–903), (3) a second α-helical bundle (HB-2; residues 716–795) and (4) a third α-helical bundle (HB-3; residues 960–1,036). The CriT-CAT domain adopts an α/β core structure composed of five parallel β-strands arranged in a twisted β-sheet, surrounded by six α-helices (Fig. 2a). A similar structural arrangement can be observed in phosphoribosyltransferases (PRTases) involved in the formation of *N*-glycosidic bonds during nucleotide synthesis^22^. PRTases contain a deep pocket for PRPP binding^23^. In Bcs3-CMP, a corresponding pocket contains two phosphate groups (PO4_1_ and PO4_2_; Extended Data Fig. 4d and Supplementary Fig. 8), is located at one end of the β-sheet, and is surrounded by α-helices α37, α38, α45 and α46, and the loops shown in Fig. 2. Structural comparison of the CriT-CAT domain with PRTases revealed that the 5-phosphate of PRPP superimposes well with PO4_2_, whereas the β-phosphate of PRPP superimposes well with PO4_1_ (Supplementary Fig. 9). Thus, molecular docking guided by homology (Methods) was used to place PRPP in an energetically favored configuration into the deep pocket of the CriT-CAT (Fig. 2d–g).

**Fig. 3:**
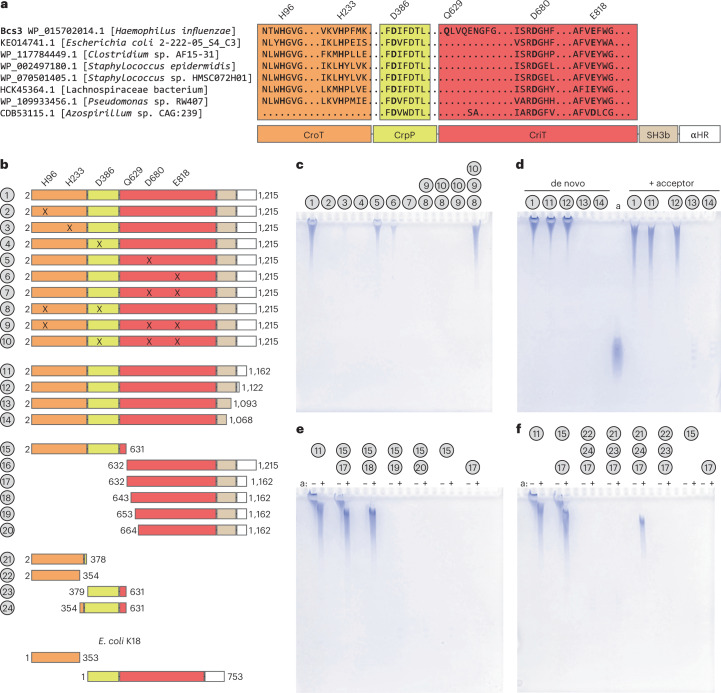
Biochemical characterization of Bcs3. **a**, Schematic representation of a multiple-sequences alignment to highlight important amino acid residues and structural elements. Distantly related hits (26–24% identity) from a BLAST^21^ search of Bcs3 are shown. An amino acid stretch unique to Bcs3 in the depicted alignment and starting with Q629 was identified and used to separate the predicted CrpP from the CriT domain. **b**, Overview of Bcs3 constructs generated in this study. Truncation constructs 21–24 were generated based on the homologs identified in *E. coli* K18, which expresses CroT as a separate polypeptide. X, amino acid exchanges to alanine; the domains are colored according to Fig. 3a. See also Supplementary Fig. 10. **c**–**f**, PAGE analysis of reactions containing the purified construct(s) (Supplementary Fig. 2) as indicated in **b**. Polymers were visualized using Alcian blue^18^. Constructs are numbered, as shown in Fig. 3a. a, acceptor (pool 3, Extended Data Fig. 7k). Source data

PRTases use the side chains of two conserved vicinal aspartates (DD motif) to accommodate the ribose ring of PRPP in the corresponding active sites^24^. In addition, the ribose C2 and C3 hydroxyl groups and the pyrophosphate moiety interact with Mg^2+^. The functional conservation of the DD motif in CriT-CAT is achieved by equivalent positioning of the side chains of D680 and E818, which are conserved in all homologs identified in this study (Fig. 3a, Supplementary Data 1 and Supplementary Fig. 9). The replacement of D680 and E818 by alanine led to a clear reduction of the Bcs3 enzymatic activity (Fig. 3b,c, constructs 5–7). The location of these residues in different structural elements, namely loop 3 and loop 1 (Fig. 2d), provides a more open topology of the CriT-CAT active site when compared to the PRTase homologs (Supplementary Fig. 9) and enables the Hib polymer to access the binding site. As a consequence, C1 from PRPP faces a putative entrance for the nonreducing end ribitol of the nascent polymer (Fig. 2e–g). Alongside this entrance, we hypothesize that E874 is ideally positioned to deprotonate 1-OH of ribitol for a nucleophilic attack (Supplementary Fig. 10). Based on homology with PRTases, we propose that CriT follows the mechanism proposed in Supplementary Fig. 11a.

### Structural basis of CrpP substrate specificity and catalysis

**Fig. 4:**
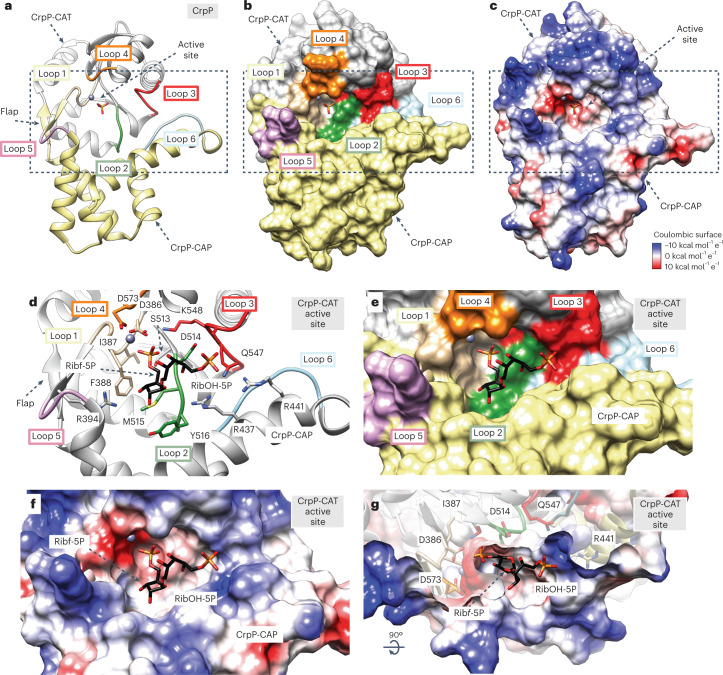
The structure of CrpP. **a**, Ribbon representation of a selected CrpP from the crystal structure of Bcs3-CMP. The orientation displays the catalytic face view revealing the overall architecture and the active site location of CrpP. Note that the cap domain CrpP-CAP (yellow) is inserted between the two β-sheets of the ‘flap’ motif, connecting CrpP-CAP and CrpP-CAT (gray). Loops flanking the active site, which contains a phosphate and a metal (Zn^2+^), are loop 1 (tan, residues 386–391, connecting β13-β14), loop 2 (dark green, residues 514–518, connecting β16-α29), loop 3 (red, residues 545–549, connecting α31-α32), loop 4 (orange, residues 573–575, connecting β18-α33), loop 5 (pink, residues 395–398, connecting β14-α33) and loop 6 (light blue, residues 444–455, connecting α25-α26). **b**,**c**, Surface representations of CrpP colored according to the color scheme and the surface Coulombic charge distribution, respectively. The latter reveals a topology with a strong negative charge at the active site responsible for housing the metal. It contrasts with the surrounding charge at the active site entrance. **d**–**f**, Detail of the active site showing the docked ligand ([OPO_3_→3)-β-d-Rib*f*-(1→1)-d-Ribitol-(5→OPO_3_]), which represents the nonreducing end of the polymer. Relevant active site residues participating in substrate binding are indicated. Note that the side chain of the catalytic residue D386 interacts with the Zn^2+^ and is close to the 5-phosphate moiety interacting with K548 (motif III; Supplementary Fig. 12). In addition, residues located in loops 1 and 2 interact with the ribose. Ribitol-5-phosphate faces away from the active site, stabilized by the interaction of the phosphate with positively charged residues at the active site entrance. **g**, Cross-section of the active site highlighting how the side chain of the catalytic residue D386 faces the metal and the phosphate pocket.

The phosphatase CrpP adopts a general topology of the haloacid dehalogenase (HAD)-like enzymes, a large superfamily of phosphohydrolases found in organisms from all three superkingdoms of life^16,25^. CrpP comprises the following two domains from the N- to the C-terminus: a catalytic Rossmann-fold domain (CrpP-CAT; residues 374–392 and 491–591, gray) and a mostly α-helical mobile cap domain (CrpP-CAP; residues 393–490, yellow; Fig. 4a–c). The CrpP-CAT comprises a five-stranded parallel β-sheet flanked by six α-helices on both sides. CrpP-CAT displays the following two key structural signature motifs that are shared by HAD-like enzymes^16^: (1) a single helical turn (residues 387–390 in CrpP, tan and loop 1) and (2) a ‘flap’ in the form of a β-hairpin motif (β14 and β15 in CrpP) located immediately after the first β-strand of the Rossmann-fold domain (Fig. 4a). These two signatures are predicted to provide the necessary mobility between the ‘open’ and ‘closed’ conformations in HAD-like enzymes^16^.

Besides the structural motifs, HAD-like phosphohydrolases are defined by the presence of four sequence motifs^16^. Motif I comprises a signature DXD located at the end of the first β-strand, right at the beginning of loop 1. The first aspartate of this motif acts as a nucleophile that forms an aspartyl-intermediate during catalysis and only this first aspartic acid is conserved in CrpP (D386; loop 1 in Fig. 4d, Supplementary Figs. 12 and 13 and Supplementary Data 1). In HAD-like phosphohydrolases, a deep pocket contains the binding site for the terminal phosphomonoester substrate. In CrpP, such a binding site could be located at one end of the β-sheet (β13, β16 and β18) and would be flanked by α-helix α33 and loops 1–4 of CrpP-CAT, as well as by the flap (β14-β15), α23, α25, α26 and loops 5 and 6 of CrpP-CAP (Fig. 4a,b). In agreement with that, a phosphate and a Zn^2+^ found in CrpP’s deep pocket superimpose well with substrates and Mg^2+^ identified in structural homologs (Fig. 4a–c, Extended Data Fig. 4a,b and Supplementary Fig. 12). Guided by the ligands and homology, we performed molecular docking, which placed one Hib repeating unit ending with a 5-phosphorylated, nonreducing end Rib*f* ([OPO_3_→3)-β-d-Rib*f*-(1→1)-d-Ribitol-(5→OPO_3_]) in an energetically favored configuration into the CrpP-CAT deep pocket (Fig. 4d–g; Methods). Notably, an internal 5-phosphorylated Rib*f* could not be placed into the pocket, supporting an exo-cleavage of the 5-phosphate over an endo-cleavage, and suggesting that CrpP acts after CriT and before CroT in the reaction cycle. Notably, the architecture of the active sites of CrpP and other HAD-like phosphatases support a common catalytic mechanism (Supplementary Fig. 11b).

### Structural basis of CroT substrate specificity and catalysis

**Fig. 5:**
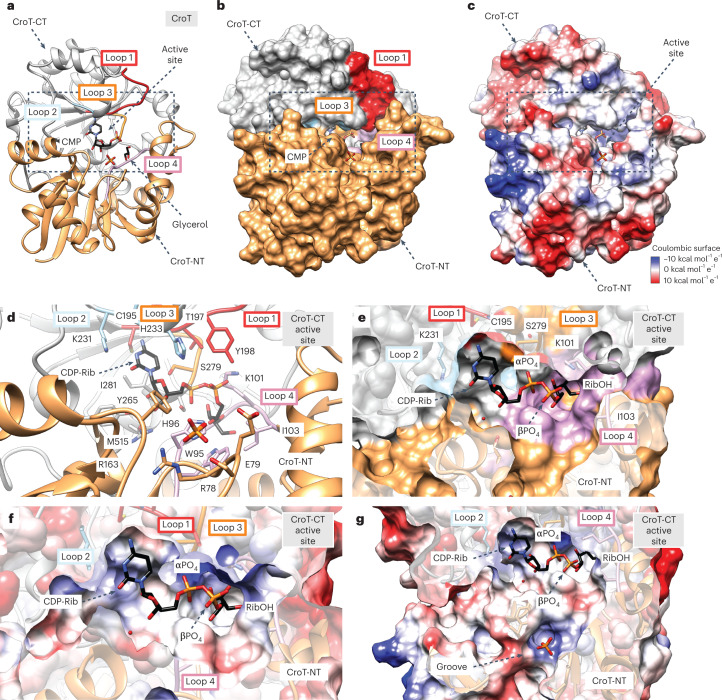
The structure of CroT. **a**, Ribbon representation of a selected CroT enzyme in the crystal structure of Bcs3-CMP. The orientation displays the catalytic face view revealing the overall architecture and the active site location. The following two Rossmann-folded domains are colored: CroT-NT (pale orange, residues 1–161 and 352–373) and CroT-CT (gray, residues 162–351). The catalytic site cleft is indicated with a dashed box. Note the position of the CMP and glycerol in the cleft and the surrounding loops forming the active site as follows: loop 1 (red, residues 195–205, connecting β7-α11), loop 2 (light blue, residues 231–233, connecting β8-α13), loop 3 (orange, residues 277–279, connecting β10-α16) and loop 4 (pink, residues 94–103, connecting β5-α4). **b**, Corresponding surface representation highlights how the closed conformation adopted by CroT buries CMP. **c**, Coulombic charge surface representation. Note that the entrance to the catalytic site shows a general positive charge distribution. **d**, Detail of the active site in ribbons showing the docked CDP-ribitol, a phosphate and active site residues. H96 and the side chains of H233 and K101 accommodate the pyrophosphate moiety. **e**,**f**, Ribitol is accommodated in a deep cavity formed by loop 4, which complements the charge distribution of the ligand. **g**, A zoomed-out view shows the NT-domain groove, which might function as a putative entrance for the acceptor substrate (nonreducing end ribose). The phosphate found in the groove indicates a potential polymer-binding site. Note that the β-phosphate of CDP-ribitol is oriented toward the putative active site entrance.

**Extended Data Fig. 6:**
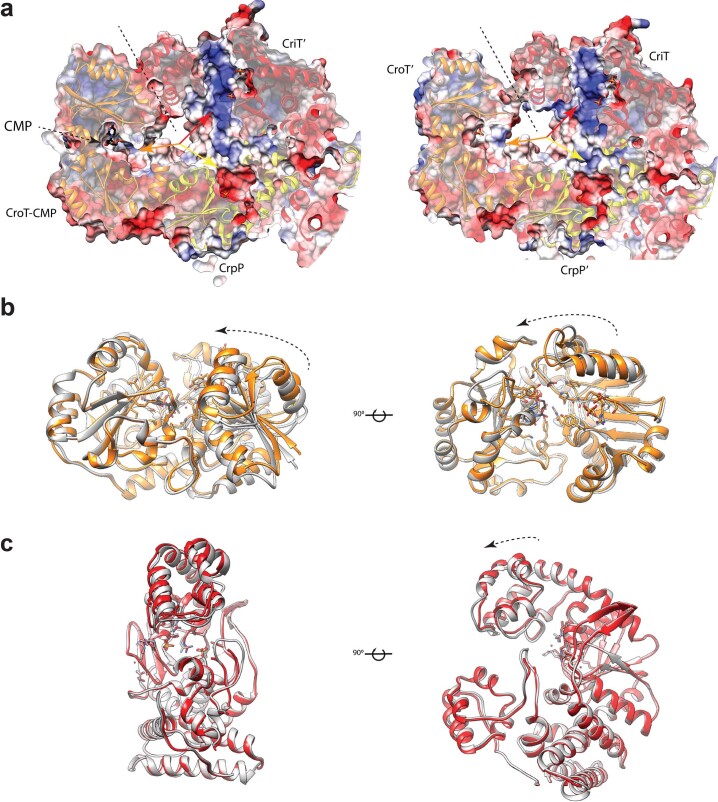
Conformational comparison between both reaction center triads in the Bcs3-CMP complex crystal structure. **a**, A surface and ribbon representation of a cross-section of the concave face of the Bcs3 dimer showing the two reaction centers, each containing one triangular active site. The reaction center triads are formed by CriT’/CrpP/CroT-CMP (*left*), and CriT/CrpP’/CroT’ (*right*). Colored arrows indicate the presence of grooves for the putative entrance of the polymer to the active sites of CroT (orange arrow), CriT (red arrow), and CrpP (yellow arrow). Observe conformational changes in the CriT-CroT’ and CriT’-CroT interphase between reaction centers, indicated by a dashed line. A closer interaction between CroT and CriT correlates with the occupation of the CroT active site by CMP. **b**, Local structural superposition of the CroT nucleotide-binding domains of the CroT-CMP complex (in orange) and CroT’ (in gray). The superposition demonstrates the relative open-to-close motion between the two CroT enzymes, with a more closed conformation for CroT-CMP and a more open conformation for CroT’, indicated by dashed arrows. **c**, Local structural superposition of CriT (in red) and CriT’ (in gray). The superposition demonstrates the relative, mostly unaffected conformation of the CriT enzymes, only presenting a slight movement for the CriT’ HB3 indicated by the dashed arrows. The above presented data suggest that conformational changes in the reaction centers are due to the entrance and release of substrates in the CroT enzymes, which likely undergo conformational changes during the catalytic cycle.

CroT displays the typical GT-B fold of glycosyltransferases (Supplementary Fig. 14), consisting of an N-terminal (CroT-NT) and a C-terminal (CroT-CT) Rossmann-fold domain, with a deep fissure at the interface that includes the catalytic center^26^ (Fig. 5a,b). The core of each domain is composed of a six-stranded parallel β-sheet flanked by α-helices on both sides (Fig. 5a). The close inspection of the electron density maps revealed a CMP molecule and glycerol located in one of the CroT protomers (Fig. 5a,b, Extended Data Fig. 3a,b and Supplementary Fig. 15). The CMP binding pocket on the C-terminal side of the interdomain crevice is defined by α-helices α8, α15 and α16, loops 1–3 of the CroT-CT domain, as well as loop 4 of the CroT-NT domain (Fig. 5a–c). A structural comparison of the CroT protomer complexed with CMP and the apo CroT protomer reveals an important interdomain movement that has been proposed for other members of the GT-B superfamily during substrate binding and catalysis (Extended Data Fig. 6 and Supplementary Fig. 16).

CroT transfers ribitol-phosphate from CDP-ribitol, generating a phosphodiester bond with the 3-OH of the nonreducing end ribose of the Hib polymer. The only other bacterial polyol-phosphate transferase whose structure was solved is the glycerol-phosphate polymerase TagF from *Staphylococcus* *epidermidis*^14^. TagF creates a phosphodiester bridge by transferring a glycerol-3-phosphate moiety from CDP-glycerol to the 1-OH of poly-glycerol-phosphate. It uses a simple displacement mechanism and by homology, we propose a similar mechanism for CroT (Supplementary Fig. 11c). To support this, we performed molecular docking calculations of CDP-ribitol into CroT (Methods). A structural comparison of the CroT-CMP complex with the TagF_H444N_-CDP-glycerol complex (PDB code 3L7K^14^) revealed that the CMP moieties of both structures superimpose well (Supplementary Fig. 14). Therefore, the ribitol-phosphate moiety was placed into the CroT active site using the observed orientation of the glycerol-3-phosphate moiety in the TagF_H444N_-CDP-glycerol complex^14^, as well as the glycerol and CMP identified in CroT as a reference. The glycerol moiety of CDP-glycerol accommodates into a small pocket flanked by P447 in TagF, whereas in CroT, the equivalent residue G99 creates extra space, presumably facilitating the ribitol-phosphate binding. In agreement with that, the proline is conserved in homologs of CroT that transfer glycerol-phosphate^15,27^, whereas the glycine is conserved in homologs transferring ribitol-phosphate^12^ (Supplementary Data 2, highlighted in yellow). The putative base H96, as well as residue H233, are strictly conserved among all homologs identified in this study and substitution to alanine abolished or drastically reduced activity (Fig. 3a–c, constructs 2–3, and Supplementary Data 1 and2).

### Structural basis of Bcs3 Hib capsule polymer recognition

**Fig. 6:**
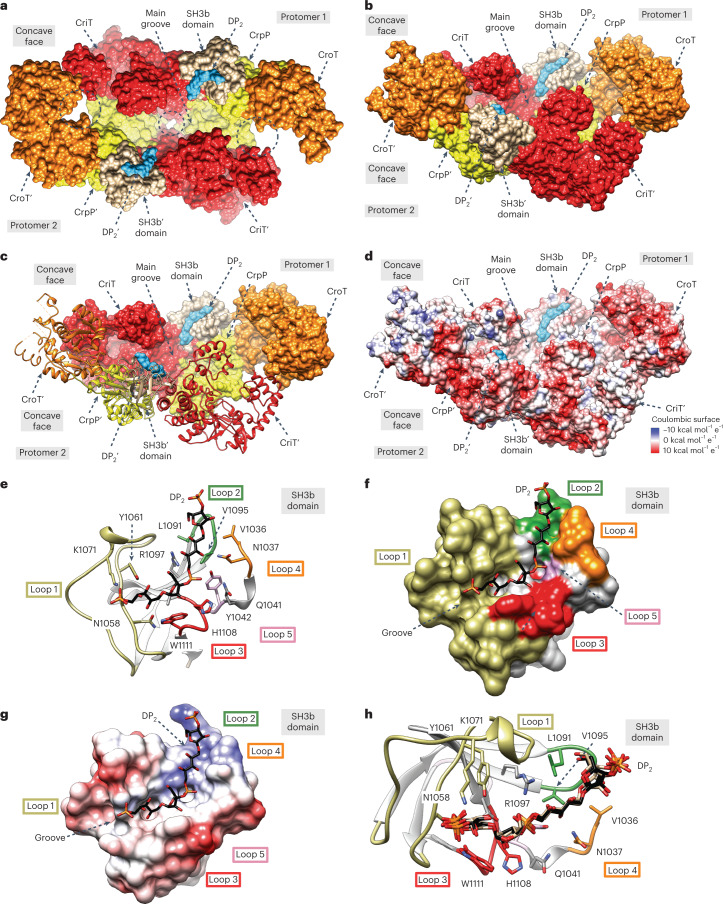
The structure of Bcs3 in complex with DP2. **a**, Surface representation of a selected dimer of Bcs3-DP2, showing two DP2 molecules depicted as cyan surfaces and each located in a concave grove of an SH3b domain. DP2 faces the common main groove of the Bcs3 dimer with its nonreducing end, at which group 2 capsule polymers are elongated^3^. **b**, The reducing end of DP2 is facing away from the main grove of the concave face. It is tempting to speculate that this arrangement allows the polymer to exit the basket-like compartment during elongation. **c**, View similar to **b** but the front protomer is depicted as ribbons. **d**, Corresponding Coulombic surface charge distribution. **e**, Detail of the SH3b domain in complex with DP2 showing the two β-sheets and the loops forming the polymer-binding groove. The first β-sheet is composed of β27, β28 and β32; the second β-sheet comprises β29, β30 and β31 (beta sheets numbered in Supplementary Data 1). The DP2 molecule accommodates into a groove defined by β30 and β31, the loops connecting β27-β28 (loop 1, the so-called RT loop in SH3 domains^28^; residues 1,056–1,079, tan), β29-β30 (loop 2; residues 1,091–1,095, dark green) and β31-β32 (loop 3; residues 1,107–1,111, red), as well as the loop connecting SH3b to the third α-helical bundle (HB-3) of CriT (loop 4; residues 1,031–1,038, orange). Residues implicated in the interactions with DP2 are depicted. **f**,**g**, Surface representation of a selected SH3b domain showing the overall fist-like shape comprising a central groove decorated by positive and neutral patches that hold the polymer in position. **h**, The superposition of all four DP2 polymer fragments belonging to all four SH3b domains shows a common overall orientation and binding mode of the ligand.

**Extended Data Fig. 7:**
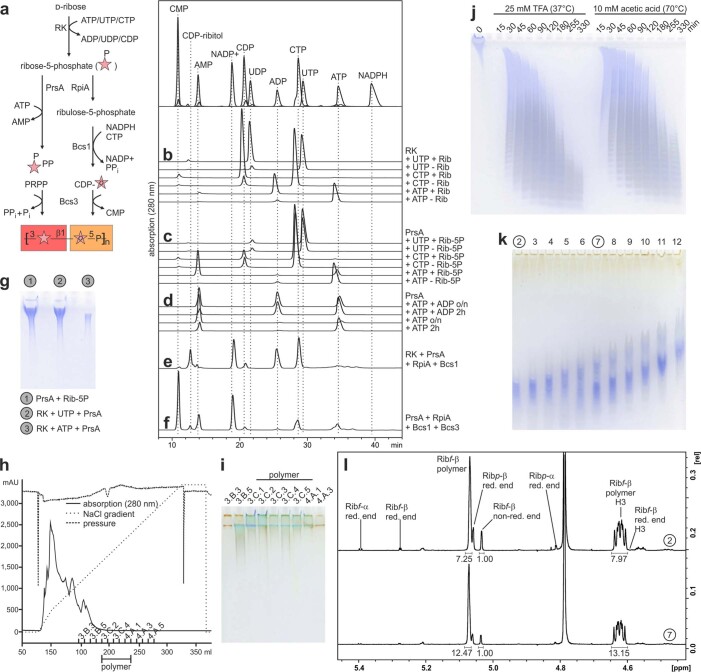
Development of a multi-enzyme cascade suitable for generating Hib polymer at mg-scale and production of oligomers. See Supplementary Note for a detailed description of the results. **a**, Schematic of the enzyme cascade for the synthesis of Hib polymer starting from d-ribose (Rib). **b-f**, HPLC-AEC analysis of samples as indicated. Enzymatic activity of (**b**) RK and (**c**) PrsA using ATP, CTP or UTP as donor substrate. Enzymatic activity of (**d**) PrsA in presence of ADP. The reaction cascade (**e**, **f**) was started with (**e**) Rib or (**f**) Rib-5P using the indicated enzymes and their substrates as shown in **a**. **g**, Alcian blue stained polyacrylamide (PA) gel of polymer synthesized starting from Rib-5P (lane 1) or Rib phosphorylated by RK using UTP (lane 2) or ATP (lane 3), respectively. **h**, Purification of polymer from an up-scaled synthesis using preparative AEC. Remaining nucleotide substrates and nucleotide products (see **a**) are UV-detectable at 280 nm, eluted early from the column, and could be separated from **i**, the UV-inactive Hib polymer, which was visualized by Alcian blue staining after PAGE. **j**, Test hydrolysis of Hib polymer in 25 mM TFA (37 °C) or 10 mM acetic acid (70 °C). After the indicated time points, samples were taken and analyzed using PAGE followed by Alcian blue / silver staining. **k**, 50 mg of Hib polymer were hydrolyzed (25 mM TFA, 120 min, 37 °C), fragments were separated by preparative AEC, collected as 12 separate pools (pool 1 not shown), and visualized by Alcian blue / silver staining after PAGE. **l**, 1D NMR analyses of the indicated oligomer pools. The annotation of the signals is based on a comprehensive 2D NMR analysis and in agreement with published literature (see Supplementary Table 2 for chemical shifts and references). The ratio between the signal intensities of the anomeric protons from the non-reducing end β-Rib*f* and the internal β-Rib*f* in the polymer allows an estimation of the average degree of polymerization of the oligomers comprised in the pool. E.g. for pool 2: 1 + 7.25 = 8.25 → oligomers of pool 2 have an average chain length of app. eight repeating units. Alternatively, the H3 signal of the internal β-Rib*f* can be used instead of the anomeric signal. Source data

To study the location of Hib capsule polymer in Bcs3, crystals were soaked with oligomer fractions having a narrowed degree of polymerization (DP) (Extended Data Fig. 7, Supplementary Note and Supplementary Table 2). A structure was solved by molecular replacement methods (3.6 Å maximum resolution) showing Bcs3 in complex with two complete repeating units (DP2) of the Hib capsule polymer and an additional nonreducing end phosphate coming from a third repeating unit (Fig. 6). Bcs3-DP2 crystallized in the *P* 1 2_1_ 1 space group, with four molecules in the asymmetric unit (Supplementary Table 3). The overall architecture between Bcs3-CMP and Bcs3-DP2 is essentially preserved. However, we observed more flexibility for the CroT domain in Bcs3-DP2 than for the CroT domain in Bcs3-CMP (Supplementary Figs. 6 and 16). DP2 is clearly visible in the electron density maps and is present in all four Bcs3 molecules bound to an SH3b domain (residues 1,037–1,115), with the nonreducing end facing toward the active centers in the main groove (Fig. 6a,b, Extended Data Fig. 3c,d and Supplementary Fig. 17). SH3b domains are the prokaryotic counterparts of the well-characterized SH3 domain family^20,28^. The SH3b domain in Bcs3-DP2 revealed a completely conserved fold—a compact β-barrel, formed by two anti-parallel, three-stranded β-sheets (Fig. 6e and Supplementary Fig. 14c).

### Molecular basis of Bcs3 enzymology

**Extended Data Fig. 8:**
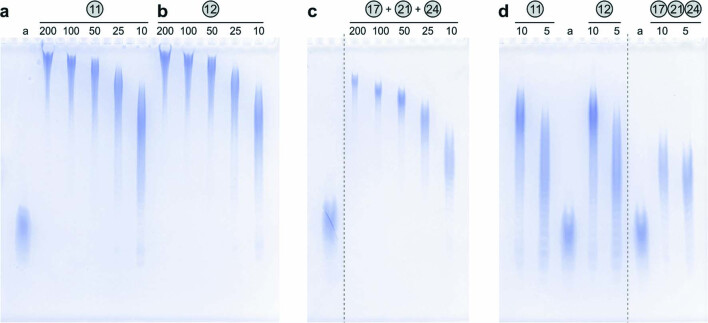
Analysis of the chain length distribution generated by Bcs3 and Bcs3 truncations. Reactions were performed with the constructs as indicated (numbering refers to Fig. 3b or Supplementary Fig. 2) at varying donor (CDP-ribitol, PRPP) to acceptor (pool 3, Extended Data Fig. 7) ratios and products were visualized after PAGE by Alcian blue staining. The donor to acceptor ratio (d/a) is indicated above each lane. a: acceptor substrate in a concentration detectable on a gel (6x higher than in a reaction with d/a of 5). **a, b**, Even at low d/a (50, 25, 10), products of considerable length are visible in reactions containing the C-terminally truncated constructs 11 (Bcs3_2–1162_) and 12 (Bcs3_2–1122_), which both contain an intact polymer binding domain. Product populations are disperse and small neglected acceptors can be detected, especially at a low d/a of for example 10 or 5 (**d**). Both observations are hallmarks of processive elongation^39^ mediated by an extended acceptor binding site and are consistent with a model in which the polymer binding domain tethers the acceptor. **c**, With the intent to disrupt the efficiency and/or processivity of the Bcs3 machine, acceptor elongation was performed using the separated CroT, CrpP, and CriT domains in *trans*-complementation reactions. Indeed, products generated using the truncations 21 (Bcs3_2–378_), 24 (Bcs3_354–631_), and 17 (Bcs3_6312–1162_) are smaller and of lower dispersity (**c, d**). Both observations are consistent with a more distributive elongation mode, putatively enforced by the domain separation and the fact that each domain carries an MBP tag, which, we hypothesize, prevents the concerted action of the domains to a certain degree. A dashed line indicates that samples not relevant for data presentation were excised. Source data

As described above, the constructs Bcs3_H96A_/Bcs3_H233A_ (CroT), Bcs3_D386A_ (CrpP) and Bcs3_D680A_/Bcs3_E818A_/Bcs3_D680A/E818A_ (CriT) were (partially) inactive (Fig. 3a–c, constructs 2–7), confirming the importance of the targeted residues for the catalytic function of the Bcs3 enzymes. Constructs 8–10, in which only one of the enzymes remained intact, could be combined in *trans* during polymer assembly, corroborating that the introduced substitutions did not alter the function of the remaining Bcs3 domains (Fig. 3b,c, last lane) and indicating that the polymer could be released from each construct despite the fact that processivity is observed for wild-type Bcs3 (Extended Data Fig. 8).

To determine the relevance of the two predicted amphipathic α-helices and the SH3b domain, we performed C-terminal truncations in Bcs3 (Fig. 3b, constructs 11–14). Interestingly, the deletion of the last 53 amino acids (Fig. 3b; Bcs3_2–__1162_, construct 11) comprising the entire amphipathic α-helical bundle (α56-α59 in Supplementary Data 1) improved the yield of purified protein by fourfold (20 mg l^−1^ (Bcs3) versus 86 mg l^−1^ (Bcs3_2–__1162_)). Because truncations can influence the ability of capsule polymerases to start the reaction de novo^29^, activity tests were performed in the presence and absence of acceptor. Both Bcs3_2–__1162_ and Bcs3_2–__1122_ maintained their enzymatic activity, indicating that the catalytic machinery is not dependent on the two predicted amphipathic α-helices (Fig. 3d). As expected, the observed polymers are slightly shorter in the presence of acceptor, indicating specific elongation and a certain control over the product length, which would be beneficial for the biotechnological use of the enzyme^29,30^ (Extended Data Fig. 8). Notably, Bcs3_2–__1093_ and Bcs3_2–__1068_, in which the SH3b domain is disrupted, were completely inactive, strongly supporting the mechanistic role of the SH3b domain for Hib capsule polymer recognition.

N-terminal truncations were performed to further advance our understanding of the CriT domain. CriT and CrpP were separated between α35 and α36, which are part of the N-terminal HB-1 of CriT (Fig. 2a). The loop connecting these structural elements is of variable length (Supplementary Data 1) or even missing (Fig. 3a) in CriT homologs identified by BLAST^21^. The resulting constructs Bcs3_2–631_, Bcs3_632–__1162_ and Bcs3_632–__1215_ were inactive alone but active if combined in *trans* (Fig. 3b,e, constructs 15–17), demonstrating that the separated enzymes indeed maintain their ability to generate polymer and that HB-1 of CriT can be partly reduced in size. Since Bcs3_632–__1162_ was purified with improved quality if compared to Bcs3_632–__1215_ (Supplementary Fig. 2c), Bcs3_632–__1162_ was N-terminally truncated to narrow down the minimal active CriT domain. The resulting Bcs3_643–__1162_, Bcs3_653–1162_ and Bcs3_664–__1162_ (Fig. 3b, constructs 18–20) were combined in *trans* with Bcs3_2–631_. The ability to initiate chain elongation de novo was only observed for Bcs3_2–631_ + Bcs3_632–1162_, with Bcs3_2–631_ + Bcs3_643–1162_ still being able to elongate priming acceptor oligosaccharides (Fig. 3e, 15 + 17, 15 + 18).

The rationale for separating CroT and CrpP was based on a comparison of Bcs3 with the capsule biosynthesis enzymes of *Escherichia* *coli* K18 (Extended Data Fig. 1b). The Hib ([→3)-β-d-Rib*f*-(1→1)-d-Ribitol-(5→OPO_3_→]_*n*_) and *E. coli* K18 ([→2)-β-d-Rib*f*-(1→2)-d-Ribitol-(5→OPO_3_→]_*n*_) capsules only differ with regard to how their constituents are linked. We identified separate open reading frames for CroT and CrpP/CriT in the K18 capsule genes cluster (Fig. 3b and Extended Data Fig. 1b). An alignment of Bcs3 with CroT-K18 and CrpP-CriT-K18 demonstrates that the region comprising α21 and α22 (amino acids 348–379) of Bcs3 is missing in *E. coli* K18, indicating that it might not be crucial for activity (Supplementary Data 3). Consequently, we designed Bcs3_2–354_, Bcs3_2–379_, Bcs3_354–631_ and Bcs3_378–631_ (Fig. 3b, constructs 21–24) and tested them in all possible combinations together with the CriT domain Bcs3_632–1162_. The activity was only visible in the presence of the acceptor and when constructs Bcs3_2–378_ + Bcs3_354–631_ were combined (Fig. 3f, 17 + 24 + 21), indicating that the truncated region is important for the function of both CroT and CrpP under in vitro conditions.

## Discussion

Gram-negative bacteria use ABC-transporter-dependent assembly systems for the expression of polymers in which charged carbohydrate moieties (sialic acid, 3-deoxy-d-*manno*-oct-2-ulosonic acid (Kdo) and glucuronic acid) or phosphate residues alternate with noncharged carbohydrates (group 2 for *E. coli* nomenclature)^31^. Polymers exclusively containing sugar units are generated by enzymes, adopting one of the two most abundant GT folds: GT-A or GT-B^3,26,32,33^. Polymerases assembling a heteropolymer, in which monosaccharides are bridged by phosphodiester linkages^29,34^, belong to a protein family called *stealth*^35^. Polyol-phosphate-containing polymers are assembled by TagF-like enzymes^15,18,27,30^. Structural data for capsule polymerases embedded in group 2 systems are limited to the chondroitin polymerase K4CP from *E. coli* K4 (ref. ^32^) and the polysialyltransferase from *Mannheimia haemolytica* serotype A2 (ref. ^33^; *Mh*PST). With Bcs3, we present the structure of a multi-enzyme machine that generates a complex heteropolymer and combines three catalytic domains that are new in the context of capsule biosynthesis.

**Extended Data Fig. 9:**
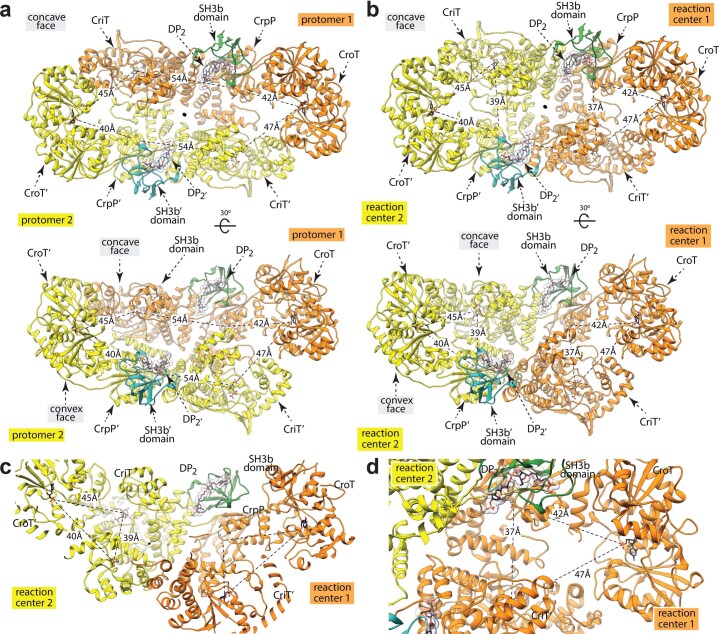
A model of action for Bcs3. The dimeric structural arrangement of Bcs3 allows the formation of **a**, a large reaction center in which the nascent chain is assembled by enzymes from both protomers - CriT/CrpP/CroT and CriT’/CrpP’/CroT’ - in a common cycle with all six enzymes working in sequential order. However, the elongated shape of the basket-like compartment, which places the CroT enzymes far away from each other, might prevent them from participating in the synthesis of a common polymeric thread. Moreover, this model would likely promote the two capsule polymers forming a coil after a few rounds of catalysis. **b**, Alternatively, two reaction centers are formed by different protomers - CriT/CrpP’/CroT’ and CriT’/CrpP/CroT - along the transversal longest axis across the main cleft of Bcs3. This model is supported by **c, d**, the fact that the active sites of the CriT/CrpP’/CroT’ and CriT’/CrpP/CroT triads are in the same plane, respectively, each separated by an angle of ca. 120°. It is worth noting that each reaction center is equidistant from the three active sites, forming a triangle (**b-d**, see also Extended Data Fig. 6a). The Bcs3-DP2 complex structure shows that the two chains attached to the corresponding SH3b domains each point to one of the reaction centers proposed by model 2. It is tempting to speculate that this structural arrangement of the active sites within a concave basket-like compartment promotes substrate channeling and facilitates the sequence of reactions, a characteristic of multi-enzyme complexes involved in efficient pathways.

**Extended Data Fig. 10:**
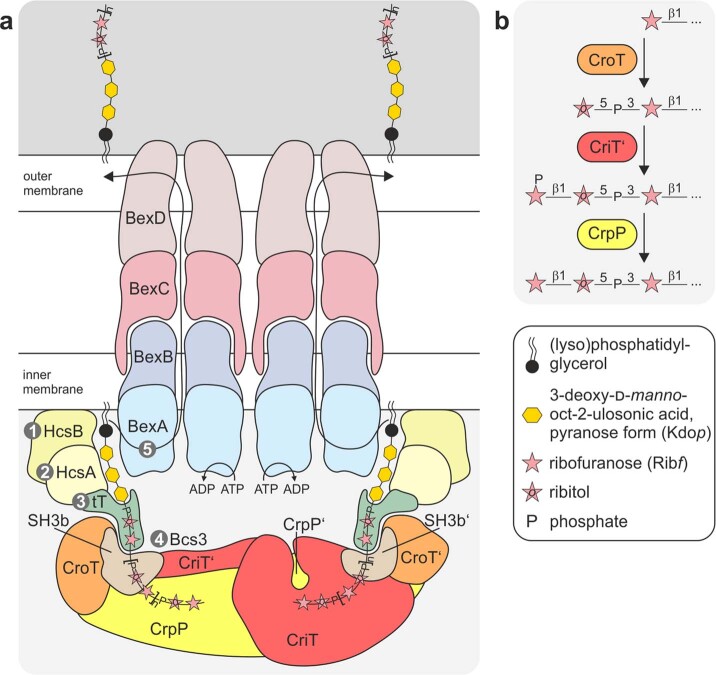
Model of Bcs3 in the context of the ABC-transporter dependent capsule assembly system of Hib. **a, (1, 2)** Capsule expression in ABC-transporter dependent assembly systems starts with the synthesis of a poly-Kdo linker attached to a (lyso)phosphatidylglycerol. This precursor is conserved in all Gram-negative bacteria utilizing the ABC-transporter dependent pathway and its assembly is catalyzed in *E. coli* by the β-Kdo transferases KpsS and KpsC^31^. KpsS transfers a single Kdo, which is then extended by KpsC. By homology, HcsB and HcsA were suggested to catalyze this reaction in Hib^71^. **(3)** One or several yet to be identified transition transferases were hypothesized to add the first repeating unit to the poly-Kdo, in order to create an acceptor for the **(4)** capsule polymerase, which assembles the capsule polymer and determines the serotype. The electrostatic surface potential of Bcs3 and its concave surface suggest an orientation in which the active centers of the enzyme face the inner membrane and/or the **(5)** ABC transporter-dependent export complex (BexA-D). The distance between the two SH3b binding domains might allow an enzymatic assembly in which each of the two chains generated by the Bcs3 dimer is exported by one ABC transporter complex. It is of note that interactions between the components of the Hib assembly system have not been experimentally proven. However, studies in *E. coli* present evidence for the formation of a multi-protein complex^72,73^. Moreover, ATP-dependent translocation coupled with Bcs3 synthesis and channeling of two polymer threads by the SH3b domain through two transporters is hypothesized as an efficient scenario for capsule expression. To allow a concise presentation, enzymes providing nucleotide substrates (for example CMP-Kdo, CDP-ribitol) are not shown. **b**, Reaction cycle of Bcs3 for the addition of one repeating unit to the non-reducing end of the nascent Hib capsule polymer.

Our structural data support a mechanistic model in which the catalytic cycle that adds one repeating unit to the nascent chain consists of three steps as follows: (1) CriT transfers Rib*f*-5P from PRPP to the C1’ OH group of the nonreducing end ribitol of the growing chain, (2) CrpP removes the phosphate group from the 5 position of the now terminal Rib*f*-5P transferred by CriT and (3) CroT catalyzes the transfer of ribitol-phosphate from CDP-ribitol to the 3 position of the ribose ring released by CrpP (Extended Data Figs. 9 and 10). Even though the dimeric structural arrangement of Bcs3 allows the nascent chain to be elongated according to two scenarios (Extended Data Fig. 9), the arrangement of CriT, CrpP, CroT and SH3b supports a model in which two reaction centers inside the concave side of Bcs3 are formed, comprising two triads of enzymes—CriT/CrpP’/CroT’ and CriT’/CrpP/CroT (Extended Data Figs. 6a and 9c). The fact that constructs 15 (CroT/CrpP) and 17 (CriT) can form a polymerase (Fig. 3e) that is even active de novo supports this hypothesis.

A polysaccharide-binding site could be identified through molecular dynamics simulations and a priming acceptor monosaccharide in the crystal structure of the hyaluronan synthase Cv-HAS from Chlorella virus^36^. However, no structural evidence is available for extended binding sites or binding modules complexed with native polymer for bacterial group 2 capsule polymerases^32,33^. The Hib capsule dimer found in Bcs3 is bound to an SH3b domain, which is located 20–40 Å away from the active sites, suggesting a tethering mechanism^37^ that allows Bcs3 to stay attached to its acceptor while the nonreducing end is elongated (Extended Data Fig. 9). It is tempting to speculate that the SH3b domain allows a sliding motion to guide the polymer to the outside of the concave Bcs3 basket. The concave walls of Bcs3 are decorated with scattered patches of positively charged residues we suspect to participate in the channeling of the capsule polymer during the catalytic cycle, possibly via phosphate interactions. We hypothesize that this allows efficient elongation^38^ and will likely contribute to the level of processivity^39^ observed in Extended Data Fig. 8 and discussed in Supplementary Discussion.

In the living cell, most physiological processes are coordinated in networks. Colocalization of enzymes in compartments promotes higher performance of individual enzyme activities and reaction efficiency^40^. The Bcs3 crystal structures reveal a complex architecture compatible with a multi-enzyme macromolecular machine that acts as a concave basket-like compartment to polymerize the Hib capsule polymer. The electrostatic surface potential of Bcs3 suggests that the concave surface—including the three active sites, the SH3b domain and the predicted amphipathic α-helices—is oriented toward the membrane. Interestingly, the exit points of the SH3b domains are approximately 70–80 Å apart, while ABC transporters exporting polymers similar to the Hib capsule have a diameter of approximately 60–70 Å^41,42^. Combined with the observation that capsule polymers are synthesized in rafts^43^, it is tempting to speculate that the Bcs3 architecture supports the bridging of two ABC-transporter complexes, thereby coupling capsule biosynthesis to export (Extended Data Fig. 10).

The identity of the activated precursor for the transfer of Rib*f* and the machinery for its incorporation into bacterial CPS has long been a matter of debate. We show that Rib*f* transfer catalyzed by Bcs3 is realized by combining a HAD-like phosphatase (CrpP) and a glycosyltransferase (CriT) of a yet undescribed GT fold. Typically, glycosyltransferases adopt Rossmann GT-A or GT-B folds and use NDP/NMP-sugars or lipid-linked donors as substrates^26^. In contrast, CriT resembles PRPP-transferases involved in the formation of *N*-glycosidic linkages^22^ but uses PRPP as a substrate for the transfer of *O*-linked ribofuranose-5-phosphate, which is dephosphorylated by CrpP. During the final preparation of this study, a study was published^44^ describing CrpP and CriT homologs, and the crystal structure of a HAD-like phosphatase and a putative ribofuranosyltransferase from *Thermobacillus composti*. A structural comparison with Bcs3 revealed considerable differences between the two structures, indicating structural variety in PRPP-dependent glycosyltransferases (Supplementary Fig. 18).

The closest homologs to the Hib polymer are complex Gram-positive WTAs, but polymerases generating complex WTA have not been identified yet^12^. WTAs make upto 60% of the cell wall of Gram-positive pathogens, extend beyond the peptidoglycan layer and are thus in direct contact with the host immune system^12,45^, making them attractive vaccine antigens^7,46^, and WTA biosynthesis enzymes promising drug targets^47^. Of the four WTA types (I–IV), only WTA I biosynthesis has been investigated thoroughly^12^. We identified the homologs of the entire Bcs3 sequence in Gram-positive pathogens (Supplementary Data 1). Future studies will show if these homologs are part of WTA biosynthesis gene clusters. Notably, BLAST searches performed herein using CriT and CroT as queries could not identify homologs in the human proteome, corroborating that mammalian enzymes^22,48^ with similar substrate usage are distinct from CriT and CroT and emphasizing the importance of the Bcs3 structure for the development of inhibitors against bacterial pathogens.

Enzymatic and chemo-enzymatic polymer synthesis is a promising strategy to provide antigens for glycoconjugate vaccine development^4,7^. It omits pathogen culture and starts with highly pure precursors^27,49^. Herein, the established multi-enzyme cascade is scalable, could be used to produce milligram amounts of polymer and allows a considerable measure of length control (Extended Data Figs. 7 and 8). The developed hydrolysis protocols yielded oligomers with free reducing ends that can be coupled through reductive amination and have the right size (for example, 10–25 repeating units) for vaccine development studies^7^. The single action transferases developed herein might even allow the step-wise build-up of the polymer according to established protocols^30^. The structural and biochemical characterization of Bcs3, the identification of homologs in both Gram-negative and Gram-positive pathogens and the development of fermentation-free synthesis protocols represent important milestones toward understanding, manipulating and exploiting multi-enzyme machines that will allow new access to structurally complex surface glycans.

## Methods

### General cloning

A PreScission cleavage site was inserted into the modified *p-Mal-c* (New England BioLabs) vector pMBP-S3N10-csxA-His_6_ (ref. ^51^) (tac) resulting in pMBP-PreScission-S3N10-csxA-His_6_. The gene *cps3B*^27^ was cloned via BamHI/XhoI into plasmid pMBP-PreScission-S3N10-csxA-His_6_, replacing the *csxA* sequence and resulting in pMBP-PreScission-S3N10-cps3B-His_6_. The genes *bcs1* (OOD27571.1), *bcs3* (OOD27573.1; Uniprot: Q2ERG0), *rpiA* (OOD27170.1), *prsA* (OOD25971.1) and *rk* (OOD27132.1) were amplified by PCR using heat-inactivated lysate of Hib strain ATCC 10211 (GenBank accession number MTGI00000000.1) as a template. The resulting PCR products were cloned into pMBP-S3N10-csxA-His_6_ via BamHI/XhoI restriction sites or pMBP-PreScission-S3N10-cps3B-His_6_ via restriction-free cloning^52^ replacing *csxA* and *cps3B*, respectively. Single amino acid mutants and truncations of Bcs3 were introduced according to ref. ^53^ or by using the Q5 Site-Directed Mutagenesis Kit (New England BioLabs) according to the manufacturer’s guidelines. Constructs and primers used in this study are listed in Supplementary Tables 4 and 5.

### Expression and purification of recombinant proteins

For the expression of recombinant proteins, *E. coli* M15 (pREP4) cells were transformed with expression plasmids and grown in 250–500 ml of PowerBroth medium (ATHENAES) supplemented with 50 μg ml^−^^1^ carbenicillin at 37 °C. When the culture reached an optical density at 600 nm (OD_600_) of 1.0, the culture was incubated at 4 °C for 30–45 min, Bcs3 expression was induced by the addition of 0.5 mM isopropyl β-d-thiogalactopyranoside (IPTG) and incubation was continued at 15 °C and 200 rpm overnight. Cells were collected by centrifugation at 6,000*g* for 10 min and resuspended in 10 ml of purification buffer (50 mM Tris, pH 8.0, 300 mM NaCl), containing EDTA-free protease inhibitors (Complete EDTA-free; Roche). Cells were sonicated (Sonorex sonication; Bandelin) for 10 cycles of 30 s each at 25–30% power and 50% amplitude, interrupted by 30 s of cooling on ice. Cell debris was separated by centrifugation at 27,000*g* for 30 min. The supernatant was filtered through a 0.8-µm cutoff membrane and protein was purified by affinity chromatography using a HisTrap column (1 ml bed volume; GE Healthcare) or MBPTrap column (1 or 5 ml bed volume; GE Healthcare), previously equilibrated in purification buffer. The enzyme was eluted using a linear gradient from 35 to 500 mM imidazole over 15 column volumes (HisTrap column) or with 10 mM maltose over 5 column volumes (MBPTrap column). Protein-containing fractions were pooled and applied to a HiPrep 26/10 Desalting column (GE Healthcare) equilibrated in purification buffer. The protein was concentrated using an Amicon centrifugal device (10,000–100,000 molecular weight cutoff, depending on protein size; Merck) and aliquots were snap-frozen in liquid nitrogen and stored at −80 °C. Aliquots of 10 µl of each purification step were analyzed by SDS-PAGE as previously described^51^. The crystallization construct MBP-Bcs3_2–1162_-His_6_ was purified using MBPTrap column as described above and shown in Supplementary Fig. 4. Protein-containing fractions were further purified by size exclusion chromatography (SEC) using a Superdex 200 10/300 GL column (GE Healthcare) equilibrated in purification buffer. PreScission digest was performed with 5.8 µg PreScission per milligram of recombinant protein overnight at 4 °C. After centrifugation at 15,000*g* for 5 min, the supernatant was applied to MBPTrap column to retain the MBP tag. The Bcs3-His_6_-containing flowthrough was further purified using a HisTrap column as described above, followed by preparative SEC. The SEC column was equilibrated using a gel filtration marker kit for protein molecular weights of 29,000–700,000 (Sigma) according to the manufacturer’s guidelines. The protein was concentrated using an Amicon centrifugal device (50,000 molecular weight cutoff; Merck) to give 10 mg ml^−1^ of protein, and aliquots were snap-frozen in liquid nitrogen and stored at −80 °C.

### Enzymatic reactions, HPLC-AEC analysis and PAGE

Enzymatic reactions were carried out with 0.5 to 2 µM of the enzyme at 37 °C in a total volume of 20 to 30 µl of assay buffer (50 mM Tris, pH 8.0, 10 mM MgCl_2_, 1 mM DTT (dithiothreitol)) containing 5 mM of each required substrate (ADP-ribose (Sigma), ATP (Roche), CTP (Sigma), NADPH (Roche), PRPP (Sigma), d-ribose (Sigma), ribose-1-phosphate (Sigma), ribose-5-phosphate (Sigma), ribulose-5-phosphate (Sigma) and UTP (Roche)). After 2 h of incubation or after overnight incubation, samples were analyzed by high-performance liquid chromatography-anion-exchange chromatography (HPLC-AEC) and high-percentage (25%) polyacrylamide gel electrophoresis (PAGE) as previously described^18^ with minor modifications—chromatograms were recorded using 5 µl sample and a linear elution gradient from 0% to 25% of mobile phase 2 (1 M NaCl, 20 mM Tris, pH 8.0) over 42 min. Data were collected using LCsolution version 1.25 SP4 (Shimadzu). Alcian blue-/silver-stained PA gels were scanned on an Amersham Imager 680 and colors were adjusted equally across the entire image to improve the visualization of Alcian blue. If in situ-synthesized ribose-5-phosphate was used as a substrate for further enzymatic reactions, 0.5–2 µM ribokinase was preincubated in a total volume of 30–50 µl assay buffer at 37 °C for 2 h and heat-inactivated at 80 °C for 5 min. The substrate concentration of d-ribose and ATP was 20 mM, resulting in 5 mM of ribose-5-phosphate in subsequent reactions.

### Upscaling of polymer synthesis and purification

For the synthesis of 5–50 mg polymer, enzymes were incubated overnight at 37 °C in 3–30 ml of assay buffer (50 mM Tris, pH 8.0, 10 mM MgCl_2_, 1 mM DTT (dithiothreitol)) supplemented with 5 mM of each substrate. In vitro-synthesized polymer was purified by AEC using a Mono Q 10/100 GL column (GE Healthcare) as described previously^30^ with the minor adjustment that polymer-containing fractions were either dialyzed against water or thoroughly washed using an Amicon centrifugal device (30,000 molecular weight cutoff; Merck).

### Preparation of oligomers

For the generation of oligomers, hydrolysis conditions for the Hib polymer (2.5 mg ml^−^^1^) were tested in (1) 25 mM TFA at 37 °C or (2) 10 mM acetic acid at 70 °C. Samples of 10 µl were collected after 15, 30, 45, 60, 90, 120, 180, 255 and 330 min of reaction time and mixed with 10 µl of 2 M sucrose. The hydrolysis products were analyzed by Alcian blue/silver strained high-percentage (25%) PAGE as previously described^18^. Preparative hydrolysis of 50 mg polymer was performed by incubation with 25 mM TFA for 120 min at 37 °C. The reaction mixture was freeze-dried and dissolved in 20 ml buffer (50 mM Tris, pH 7.0, 100 mM NaCl, 10 mM MgCl_2_). Dephosphorylation of hydrolysis products was performed by the addition of 200 U alkaline phosphatase (Quick CIP; New England BioLabs) for 75 min at 37 °C, followed by the addition of another 100 U of alkaline phosphatase and incubation for 75 min at 37 °C. Alkaline phosphatase was removed using an Amicon centrifugal device (50,000 molecular weight cutoff; Merck). The oligomer-containing filtrate was diluted to 140 ml and further purified by AEC (Mono Q 10/100 GL; GE Healthcare) using a linear gradient from 0 mM to 350 mM NaCl over 40 column volumes. After analysis by high-percentage PAGE, fractions were divided into 12 pools, dialyzed against water (Spectra/Por 7, 1,000 molecular weight cutoff; Roth) and then freeze-dried (Extended Data Fig. 7).

### Dot blot/agglutination assay

Dot blot analysis of purified polymer was performed as previously described^51^. Hib polymer was detected using a rabbit anti-Hib agglutinating serum (Remel/Thermo Fisher Scientific; 1:1,000 or 1:2,000 dilution).

### NMR analysis

All spectra were measured on a 600-MHz Bruker Avance III HD equipped with a ^1^H/^13^C/^15^N/^31^P QXI probe at 298 K. Typically, samples were dissolved in 500 μl D_2_O (100.0 atom%; Armar Chemicals) and measured in a 5-mm NMR standard tube. ^1^H 1D spectra were recorded with eight transients and a recycle delay of 10 s. ^31^P 1D spectra were recorded with a recycle delay of 3 s and 352 transients. Standard ^1^H-^13^C HSQC experiments from the Bruker library (hsqcedetgpsisp2.2) were recorded with 32 scans, 2,048 × 230 points and a recycle delay of 1.5 s. ^1^H-^1^H TOCSY spectra were measured with four scans, 2,048 × 360 points, a recycle delay of 1.5 s and a mixing time of either 80 ms or 12 ms. ^1^H-^13^C HMBC spectra (Bruker pulse sequence: hmbclpndqf) were recorded with 32 scans, 4,096 × 512 points and a recycle delay of 1.5 s. ^1^H-^31^P HMBC spectra (Bruker pulse sequence: hmbcgpndqf) were recorded with 16 scans, 4,096 × 320 points and a recycle delay of 1.5 s. All spectra were processed using Topspin 3.6 (Bruker) and analyzed using Sparky 3.111 and 3.115 (T. D. Goddard and D. G. Kneller, SPARKY 3, University of California, San Francisco, CA, USA). Proton chemical shifts were calibrated to 2,2-dimethyl-2-silapentane-5-sulfonic acid (DSS) using an external sample of 2 mM sucrose and 0.5 mM DSS in H_2_O/D_2_O (Bruker). Indirect chemical shift referencing was applied to ^13^C and ^31^P according to IUPAB^54^ using the chemical shift referencing ratios of 0.251449530 and 0.404808636.

### Bcs3 crystallization and data collection

The Bcs3-CMP complex was crystallized by mixing 0.25 µl of a protein solution (9.5 mg ml^−^^1^ in 50 mM Tris, pH 8.0, 300 mM NaCl, 1 mM DTT) in the presence of 5 mM CMP with 0.25 µl of 0.04 M potassium phosphate monobasic, 16% (wt/vol) PEG 8000 and 20% (vol/vol) glycerol (JCSG + commercial screening, Molecular Dimensions). Crystals were frozen under liquid nitrogen. Complete X-ray diffraction datasets were collected at the beamline i24 using a beam transmission equal to 16.33% (Diamond Light Source Synchrotron). Bcs3-CMP crystallized in the *P* 2_1_2_1_2_1_ space group with two molecules in the asymmetric unit and diffracted to a maximum resolution of 2.9 Å (Supplementary Table 3). Bcs3-DP2 was crystallized by mixing 0.25 µl of protein solution (9.5 mg ml^−^^1^ in 50 mM Tris, pH 8.0, 300 mM NaCl, 1 mM DTT) with 0.25 µl of 0.06 M magnesium chloride hexahydrate, 0.06 M calcium chloride dihydrate, 0.1 M Imidazole, 0.1 M MES monohydrate, pH 6.5, 37.5% (vol/vol) MPB, 37.5% (vol/vol) PEG 1000 and 37.5% (vol/vol) PEG 3350 (Morpheus, Molecular Dimensions). The crystal was plunged frozen in liquid nitrogen. Complete X-ray diffraction datasets were collected at the beamline iO4 using a beam transmission equal to 100% (Diamond Light Source Synchrotron). Bcs3-DP2 crystallized in the *P* 1 2_1_ 1 space group with four molecules in the asymmetric unit and diffracted to a maximum resolution of 3.6 Å (Supplementary Table 3). The dataset was processed in situ using the Automatic Software Pipeline available at Diamond Light Source Synchrotron, integrated and scaled through the xia2 system.

### Bcs3 structure determination and refinement

The structure determination of the Bcs3-CMP complex was performed by molecular replacement methods implemented in Phaser^55^ and the PHENIX suite^56^. The initial coordinates of Bcs3 were generated by using a model predicted by AlphaFold as a search model^19^. The model rebuilding was carried out with Buccaneer^57^ and the *CCP4* suite^58^. The final manual building was performed with Coot^59^ and refinement with phenix.refine^60^ and Refmac5 (ref. ^61^). The structure was validated by MolProbity^62^. The Bcs3-DP2 complex was solved by molecular replacement using the Bcs3-CMP complex as a search model. Data collection and refinement statistics are presented in Supplementary Table 3. Molecular graphics and structural analyses were performed with the UCSF Chimera package^63^.

### Structural analysis and sequence alignment

Homologs of Bcs3 were identified by BLAST^21^, aligned using Clustal Omega^64^, MUSCLE^65^ or Cobalt^66^ and annotated using Jalview^67^. Structure-based sequence alignment analysis was performed using Chimera^63^. Protein pocket volume was calculated using HOLLOW^68^. Z-score values were produced using DALI^69^. Domain interface analysis was performed using PISA^70^. Conserved and similar residues were labeled using the BoxShade server (http://embnet.vital-it.ch/software/BOX_form.html).

### Molecular substrate docking

The substrate binding analysis of the active sites has been performed for CroT, CrpP and CriT using a mixed approach based on (1) the experimental location of phosphate groups and/or metals at the active site, (2) placement of the substrate by homology and (3) the minimization of the substrate docked ligand using USCF Chimera. For CroT, the modeling of CDP-ribitol in the active site was achieved firstly by placing CDP-glycerol from the complex TagF mutant N444H (PDB 3L7K)^14^ by structural superposition. Then, the docked CDP-glycerol was modified to CDP-ribitol using UCSF Chimera editing tools and following the orientation of CMP and glycerol found in the CroT active site. Finally, docking was performed using a tight search box to minimize the position of CDP-ribitol using Vina. For CrpP, the terminal 5-phospho-d-ribose end of a 5-phospho-d-Rib*f*-β-(1→1)-d-Ribitol-5-phosphate was placed in the active site using the phosphate found in the structure as a reference. A guided docking was performed following the orientation of substrates in CrpP homologs: (1) the structure of pyridoxal phosphate phosphatase in complex with a non-hydrolyzable pyridoxal 5-phosphate analog and Mg^2+^ (PDB code 5AES) and (2) the structure of phosphoserine phosphatase in complex with phosphoserine (PDB 1L7P). The docking of the moiety was oriented according to the substrates in the homologous structures and minimized in Chimera. Finally, for CriT, the placing of a PRPP-Mg^2+^ complex at the active site was guided by the position of the two phosphates found in the active site, and docked by homology extracting a ligand displaying an overall consensus orientation from the following four PRTase homologs in complex with PRPP-Mg^2+^: hypoxanthine-guanine phosphoribosyltransferase (PDB code 1FSG), adenine phosphoribosyltransferase (PDB code 1ZN7), orotate phosphoribosyltransferase (PDB code 1LH0) and uracil phosphoribosyltransferase (PDB code 1JLS). All dockings were thoroughly inspected and confronted with the protein surfaces to verify the absence of clashes.

### Statistics and reproducibility

Alignments were performed once with each algorithm, but separate algorithms, for example, MUSCLE, Cobalt and Clustal omega, yielded highly similar results regarding the critical amino acids targeted in this study. Phyre2 homology modeling was performed in 2015, 2018, 2019, 2020 and 2021 to reevaluate the results and include potential new entries to the PDB. All submissions yielded highly similar results. The detection of Hib polymer (Fig. 2f) using agglutination sera was repeated two times with similar results and the polymer structure was confirmed by NMR. Qualitative analysis of polymer production by different Bcs3 constructs or construct combinations (Fig. 3c–f and Supplementary Fig. 10) was performed at least three times with similar results. Constructs were purified once, each purification was documented by SDS-PAGE and separate Coomassie-stained gels displaying all constructs were included in this study (Supplementary Figs. 2 and 10a). CDP-ribitol was purified at least three times with similar results. Bcs3 activity in the presence of purified CDP-ribitol was analyzed twice by HPLC and a representative sample was chosen to be displayed by PAGE (Supplementary Fig. 3d). The crystallization construct was purified twice with similar results using the protocol shown in Supplementary Fig. 4. Four enzymatic syntheses of Hib polymer (yielding 5 mg, 2 × 30 mg and 50 mg of Hib polymer) were performed with similar results, and representative documentation of these reactions and the purification of the polymer are shown in Extended Data Fig. 2 and 7. The effect of ATP and UTP on the enzyme cascade was analyzed twice by HPLC and Alcian blue staining after PAGE (Extended Data Fig. 7g). Test hydrolysis of Hib polymer (Extended Data Fig. 7j) and purification and visualization of oligomers (Extended Data Fig. 7k) were performed once. To analyze the elongation mechanism of polymerase constructs, at least five reactions were performed at different donor-to-acceptor ratios with highly consistent results (Extended Data Fig. 8).

### Reporting summary

Further information on research design is available in the Nature Portfolio Reporting Summary linked to this article.

## Online content

Any methods, additional references, Nature Portfolio reporting summaries, source data, extended data, supplementary information, acknowledgements, peer review information, details of author contributions and competing interests, and statements of data and code availability are available at 10.1038/s41589-023-01324-3.

## Supplementary information

Supplementary Information Supplementary Tables 1–5, Supplementary Figs. 1–22 (Supplementary Figs. 19–22 contain supporting data for Supplementary Figs. 2, 3, 4 and 10), Supplementary Note and Supplementary Discussion.

Reporting Summary

Supplementary Data 1 Sequence alignment of Bcs3 homologs—A BLAST^69^ search was performed using the amino acid sequence of full-length Bcs3 as query and hits with high sequence coverage were manually chosen, aligned using Cobalt^70^ and annotated using Jalview^71^. Homologs from Gram-negative and Gram-positive pathogens are highlighted in dark gray (top half of the alignment) or light gray (bottom half), respectively. Database references are provided. The secondary structural elements extracted from a selected protomer of Bcs3-CMP are shown above the alignment. Amino acid residues with at least 70 % of identity are highlighted in black. A sequence logo plot is depicted below the alignment to provide a detailed overview of the general consensus at every position. Amino acids H96, H233, D386, D680 and E818, which were targeted in this study by site-directed mutagenesis, are shown in red. The consensus of the DUF5776 according to the conserved domain database^51^ for the SH3b domain is shown in blue, highlighting that this domain is present in the majority of identified homologs. Interestingly, the C-terminal alpha-helical bundle (α56-α59) is only present in homologs having a Gram-negative background. The C-terminus of the *E. coli* sequence was manually aligned to allow a gap between α53 and α56.

Supplementary Data 2 Sequence alignment of CroT homologs—Biochemically characterized CroT homolgoues from Gram-negative (gray) and Gram-positive (light gray) sources were aligned using MUSCLE^72^ and the alignment was annotated using Jalview^71^. Database references are provided. Homologs transferring ribitol-phosphate and glycerol-phosphate are indicated by full and empty circles, respectively^9,73–76^. The secondary structural elements extracted from a selected CriT from Bcs3-CMP are shown above the alignment. Amino acid residues with >70 % of identity are highlighted in black. A sequence logo plot is depicted below the alignment to provide a detailed overview of the general consensus at every position. Amino acids H96 and H233, which were targeted in this study by site-directed mutagenesis, are shown in red. Glycine and proline residues putatively determining the enzymes’ preference for ribitol and glycerol, respectively, are highlighted in yellow.

Supplementary Data 3 Sequence alignment of Bcs3 with homologs from *E. coli*—Bcs3 was aligned using MUSCLE^72^ with the putative CroT-K18 (STI11141.1) and CrpP-CriT-K18 (STI11142.1) from *E. coli* K18 (see Extended Data Fig. 1), and with a homolog (STP42972.1) identified by BLAST (see Supplementary Data 1) in a non-serotyped *E. coli* strain, which expresses all three domains as single polypeptide and has high sequence identity with CroT-K18 and CrpP-CriT-K18. The secondary structural elements extracted from a selected protomer from Bcs3-CMP are shown above the alignment. The color code introduced for CroT, CrpP, CriT and SH3b in Figs. 1 and 3 was used to color the Bcs3 sequence. Amino acids targeted to separate the Bcs3 enzymes are highlighted in gray.

## Data Availability

The atomic coordinates and structure factors have been deposited in the Protein Data Bank, accession codes 8A0C for the Bcs3-CMP complex and 8A0M for the Bcs3-DP2 complex. Data collection and refinement statistics are presented in Supplementary Table 3. PDB IDs used in the analysis of this work include 1ZN7, 1LH0, 1FSG, 1JLS, 1L7N, 5AES, 3VAY, 1L7P, 3L7K, 4ZHT, 6JDT, 4X1T, 5LEO, 6RJE, 1R77, 7BNH and 7SHG. Accession codes for sequences used in this study are available in Supplementary Table 4 and Extended Data Fig. 1. NMR chemical shifts are presented in Supplementary Tables 1 and 2. Source data are provided in separate files with this paper.

## Source data

Source Data Fig. 1 Unprocessed gel and blot.
Source Data Fig. 3 Unprocessed gels.
Source Data Extended Data Fig. 2 Unprocessed gel.
Source Data Extended Data Fig. 7 Unprocessed gels.
Source Data Extended Data Fig. 8 Unprocessed gels.
