# Supplementary material for: A multi-enzyme machine polymerizes the Haemophilus influenzae type b capsule
Source: Nat Chem Biol. 2023 Jun 5;19(7):865–77. doi: 10.1038/s41589-023-01324-3 (PMC10299916; doi:10.1038/s41589-023-01324-3)
Supplement: Supplementary file 1 — Supplementary Tables 1–5, Supplementary Figs. 1–22 (Supplementary Figs. 19–22 contain supporting data for Supplementary Figs. 2, 3, 4 and 10), Supplementary Note and Supplementary Discussion. [file 41589_2023_1324_MOESM1_ESM.pdf]

nature chemical biology

Article

https://doi.org/10.1038/s41589-023-01324-3

A multi-enzyme machine polymerizes the *Haemophilus influenzae* type b capsule

In the format provided by the authors and unedited

TABLE OF CONTENTS

Supplementary Tables

Supplementary Table 1 | NMR analysis of the Bcs3 product.

Supplementary Table 2 | NMR analysis of hydrolyzed Hib polymer.

Supplementary Table 3 | Data collection and refinement statistics.

Supplementary Table 4 | Overview of plasmids and recombinant proteins used in this study.

Supplementary Table 5 | Primers used in this study.

Supplementary Figures

Supplementary Fig. 1 | The TagF-like capsule polymerase family.

Supplementary Fig. 2 | Coomassie-stained polyacrylamide gels of all constructs (final pools) used in this study.

Supplementary Fig. 3 | Purification of CDP-ribitol.

Supplementary Fig. 4 | Purification of the crystallization construct MBP-S3N10-PreScission-$Bcs3_{2-1162}$-His_6.

Supplementary Fig. 5 | The overall structure of Bcs3.

Supplementary Fig. 6 | Stereo views of maps for the Bcs3 dimers.

Supplementary Fig. 7 | Alphafold model of the C-terminal amphipathic α-helical bundle of Bcs3.

Supplementary Fig. 8 | CriT active site environment and phosphate binding sites.

Supplementary Fig. 9 | Structural comparison of CriT and PRTase homologs in complex with PRPP-Mg^{2+}.

Supplementary Fig. 10 | E874 of CriT is a promising candidate for the base that activates 1-OH of the non-reducing end ribitol for the nucleophilic attack on ribose C1 of PRPP.

Supplementary Fig. 11 | Overview of the putative catalytic mechanisms utilized by CroT, CrpP, and CriT.

Supplementary Fig. 12 | Structural comparison of CrpP and HAD phosphatase homologs.

Supplementary Fig. 13 | CrpP active site environment and phosphate and metal binding site.

Supplementary Fig. 14 | Structural comparison of CroT and SH3b domains with homologs.

Supplementary Fig. 15 | CroT active site environment and CMP and glycerol binding sites.

Supplementary Fig. 16 | Structural superposition of protomer chains found in the Bcs3-CMP and Bcs3-DP2 crystal structures.

Supplementary Fig. 17 | DP2 binding site.

Supplementary Fig. 18 | Structural comparison of CriT and CrpP from Bcs3 with the HAD-like phosphatase and the putative ribofuranosyltransferase from *T. composti*.

Supplementary Fig. 19 | Source Data for Supplementary Figure 2.

Supplementary Fig. 20 | Source Data for Supplementary Figure 3.

Supplementary Fig. 21 | Source Data for Supplementary Figure 4.

Supplementary Fig. 22 | Source Data for Supplementary Figure 10.

Supplementary Notes

Supplementary Note 1 | Development of a multi-enzyme cascade suitable for generating Hib polymer at mg-scale and production of oligomers.

Supplementary Discussion

Supplementary Discussion | The elongation mechanism of group 2 capsule polymerases.

Supplementary References

SUPPLEMENTARY TABLES

Supplementary Table 1 | NMR analysis of the Bcs3 product. Observed chemical shifts of the polymer dissolved in D_2O, measured at 298 K, and referenced to DSS in comparison with previously reported values[1].

Moiety	H1	H1'	H2	H3	H4	H5a	H5b	C1	C2	C3	C4	C5	P	
Rib*f*-β	5.092	—	4.283	4.641	4.214	3.912	3.718	109.4	76.5	77.0	84.8	65.1	—	this work
	5.091	—	4.271	4.641	4.215	3.904	3.717	109.4	76.4	77.1	84.7	65.1	—	Lemercinier et al.[a,b]
Ribitol-5P	3.889	3.761	3.997	3.799	3.965	4.126	4.011	71.4	72.8	74.2	73.6	69.5	-2.64	this work
	3.876	3.787	3.996	3.796	3.951	4.130	4.021	71.2	72.9	73.5	73.6	69.4	-2.59	Lemercinier et al.[a,b]

[a] the reported values showed an offset. For better comparison, a correction of +0.025 ppm was added to the ^{1}H values from the publication; +1.35 ppm was added to the ^{13}C values; 3.52 ppm was subtracted from the ^{31}P values to correct for the different referencing of 85 % H_3PO_4 (capillary) versus 10 % trimethylphosphate, 2 mM DSS in 99.8 % D2O, 25°C according to the recommendations of the Biological Magnetic Resonance Data Bank[2].

[b] the reported values were measured at 303 K.

Supplementary Table 2 | NMR analysis of hydrolyzed Hib polymer. Observed chemical shifts of the hydrolyzed polymer dissolved in D_2O in comparison with reported values. The data were measured at 298 K and are referenced to DSS.

Moiety	H1	H1'	H2	H3	H4	H5	H5'	C1	C2	C3	C4	C5	
polymer													
Rib*f*-β	5.070	—	4.263	4.623	4.192	3.897	3.708	109.5	76.5	77.1	84.8	65.1	this work
	5.091	—	4.271	4.641	4.215	3.904	3.717	109.4	76.4	77.1	84.7	65.1	Lemercinier et al.[a,b]
Ribitol-5P	3.874	3.741	3.982	3.784	3.949	4.107	3.998	71.3	72.9	74.1	73.6	69.4	this work
	3.876	3.787	3.996	3.796	3.951	4.130	4.021	71.2	72.9	73.5	73.6	69.4	Lemercinier et al.[a,b]
non-red. end													
Rib*f*-β	5.036		4.114	4.264	4.036	3.840	3.677	109.6	77.1	73.4	85.5	65.3	this work
								109.3	77.0	74.0	85.1	65.1	Garegg et al. 1980[c]
red. end													
Rib*f*-α	5.397		4.217	4.496	4.327	3.784	3.702	98.9	73.4	76.3	85.5	65.0	this work
	5.40												Ravenscroft et al.[d]
	5.40		4.13	4.13	4.15	3.78	3.67	99.0	73.8	72.8	85.8	64.1	Napolitano et al.[e]
								99.1	73.7	72.8	85.8	64.2	King-Morris et al.[f]
Rib*f*-β	5.279		4.132	4.584		3.878	3.697	103.5	77.3	76.8		65.0	this work
	5.30												Ravenscroft et al.[d]
	5.27		4.02	4.24	4.00	3.84	3.69	103.8	78.1	73.3	85.3	65.4	Napolitano et al.[e]
								103.7	78.0	73.2	85.3	65.3	King-Morris et al.[f]
Rib*p*-α	4.815		4.032					96.6	70.1				this work
	4.81												Ravenscroft et al.[d]
								96.3	72.8	72.0	70.1	65.9	King-Morris et al.[f]
Rib*p*-β	5.064		3.710	4.563	4.013	3.954	3.770	96.9	73.3	75.9	70.2	65.8	this work
	5.05			4.54									Ravenscroft et al.[d]
								96.6	73.8	71.7	70.0	65.8	King-Morris et al.[f]

[a] The reported values showed an offset. For better comparison, a correction of +0.025 ppm was added to the ^{1}H values from the publication; +1.35 ppm was added to the ^{13}C values; 3.52 ppm was subtracted from the ^{31}P values to correct for the different referencing of 85 % H_3PO_4 (capillary) versus 10 % trimethylphosphate, 2 mM DSS in 99.8 % D2O, 25°C according to the recommendations of the Biological Magnetic Resonance Data Bank[2,3].

[b] The reported values were measured at 303 K.

[c] Values for Rib*f*-β-ribitol reported by Garegg et al.[4]. Because the reported values showed an offset, a correction of +1.8 ppm was added to the ^{13}C values for better comparison.

[d] Chemical shifts of the hydrolyzed Hib polymer; ^{1}H values were estimated from Figure 3 in Ravenscroft et al.[5].

[e] Chemical shifts reported for the free monosaccharides from Napolitano et al.[6]. Because the reported values showed an offset, a correction of +0.14 ppm was added to the ^{1}H values from the publication and +1.3 ppm was added to the ^{13}C values for better comparison.

[f] Chemical shifts reported for the free monosaccharides from King-Morris et al.[7]. Because the reported values showed an offset, a correction of +1.3 ppm was added to the ^{13}C values for better comparison.

Supplementary Table 3 | Data collection and refinement statistics.

	Bcs3-CMP complex	Bcs3-DP2 complex
PDB	8A0C	8A0M
Beamline	I24 Diamond	I04 Diamond
Wavelength	0.9999	0.9795
Resolution range	56.8 - 2.9 (3.004 - 2.9)	63.94 - 3.6 (3.729 - 3.6)
Space group	P 21 21 21	P 1 21 1
Unit cell		
a, b, c	66.9703 164.78 302.567	127.911 232.806 129.177
α, β, γ	90 90 90	90 118.39 90
Total reflections	1442611 (144994)	541911 (51165)
Unique reflections	75365 (7378)	76828 (7658)
Multiplicity	19.1 (19.7)	7.1 (6.7)
Completeness (%)	99.90 (99.73)	99.71 (99.75)
Mean I/sigma(I)	5.70 (1.30)	8.79 (1.38)
Wilson B-factor	43.94	100.15
R-merge	0.3687 (0.9486)	0.1692 (0.8142)
CC1/2	0.98 (0.846)	0.988 (0.796)
CC*	0.995 (0.957)	0.997 (0.942)
Reflections used in refinement	75340 (7372)	76616 (7639)
R-work	0.1971 (0.2523)	0.2201 (0.2865)
R-free	0.2568 (0.3046)	0.2609 (0.3432)
CC(work)	0.928 (0.800)	0.904 (0.806)
CC(free)	0.902 (0.763)	0.882 (0.763)
Number of non-hydrogen atoms	18885	32156
Macromolecules	18341	31944
Ligands	158	364
Solvent	440	12
Protein residues	2229	4162
RMS(bonds)	0.003	0.003
RMS(angles)	0.48	0.55
Ramachandran favored (%)	95.28	95.15
Ramachandran allowed (%)	4.50	4.58
Ramachandran outliers* (%)	0.22	0.27
Rotamer outliers (%)	1.32	0.03
Clashscore	4.18	11.69
Average B-factor	43.07	99.26
Macromolecules	43.31	99.08
Ligands	50.99	128.80
Solvent	31.45	86.30

Statistics for the highest-resolution shell are shown in parentheses.

*Ramachandran outliers were mostly found in poorly defined density loops.

Supplementary Table 4 | Overview of plasmids and recombinant proteins used in this study. The construct used for crystallization studies is shown in bold.

Protein	Accession number	Identifier	Recombinant construct	MW	Primer
RpiA	OOD27170.1	5186	MBP-S3N10-Prescission-RpiA-His$_6$	66939	TF144/TF145
Bcs1	OOD27571.1	5189	MBP-S3N10-Prescission-Bcs1-His$_6$	96155	TF142/TF143
PrsA	OOD25971.1	5592	MBP-S3N10-Prescission-Ribose-P-pyrophosphokinase-His$_6$	78104	AB171/AB172
RK	OOD27132.1	5972	MBP-S3N10-Prescission-Hib-Ribokinase-His$_6$	75941	AB235/AB236
		5973	Ribokinase-His$_6$	33218	AB237/AB236
Bcs3	OOD27573.1	5191	MBP-S3N10-Bcs3-His$_6$	185312	IB036/IB037
		5701	MBP-S3N10-Bcs3$_{2-1162}$-His$_6$	179028	IB036/AB202
		5703	MBP-S3N10-Bcs3$_{632-1215}$-His$_6$	111283	AB206/IB37
		5784	MBP-S3N10-Bcs3$_{2-1068}$-His$_6$	168270	IB036/AB224
		5849	**MBP-S3N10-Prescission-Bcs3$_{2-1162}$-His$_6$**	179912	AB228/AB202
		5859	MBP-S3N10-Prescission-Bcs3$_{2-1122}$-His$_6$	175434	AB228/AB229
		5879	MBP-S3N10-Prescission-Bcs3$_{2-1093}$-His$_6$	172081	AB228/AB230
		5892	MBP-S3N10-Prescission-Bcs3$_{632-1162}$-His$_6$	105883	AB231/AB202
		5996	MBP-S3N10-Prescission-Bcs3$_{664-1162}$-His$_6$	102159	AB246/AB202
		6066	MBP-S3N10-Prescission-Bcs3$_{643-1162}$-His$_6$	104645	AB250/AB202
		6067	MBP-S3N10-Prescission-Bcs3$_{653-1162}$-His$_6$	103422	AB251/AB202
		6179	MBP-S3N10-Prescission-Bcs3$_{2-631}$-His$_6$	118037	AB228/AB258
		6368	MBP-S3N10-Prescission-Bcs3$_{2-354}$-His$_6$	85366	AB268/AB269
		6369	MBP-S3N10-Prescission-Bcs3$_{2-378}$-His$_6$	88101	AB268/AB270
		6370	MBP-S3N10-Prescission-Bcs3$_{354-631}$-His$_6$	76736	AB271/AB272
		6371	MBP-S3N10-Prescission-Bcs3$_{379-631}$-His$_6$	73944	AB273/AB272
		5627	MBP-S3N10-Bcs3$_{H96A}$-His$_6$	185246	AB178/AB179
		5628	MBP-S3N10-Bcs3$_{H233A}$-His$_6$	185246	AB180/AB181
		5631	MBP-S3N10-Bcs3$_{D386A}$-His$_6$	185268	AB189/AB190
		5699	MBP-S3N10-Bcs3$_{D680A}$-His$_6$	185268	AB200/AB201
		5732	MBP-S3N10-Prescission-Bcs3$_{E818A}$-His$_6$	185254	AB209/AB217
		5824	MBP-S3N10-Bcs3$_{D680A/E818A}$-His$_6$	185210	AB200/AB201
		5911	MBP-S3N10-Bcs3$_{H96A/D386A}$-His$_6$	185202	AB178/AB179
		5912	MBP-S3N10-Bcs3$_{H96A/D680A/E818A}$-His$_6$	185144	AB178/AB179
		5913	MBP-S3N10-Bcs3$_{D386A/D680A/E818A}$-His$_6$	185166	AB189/AB190
		6507	MBP-S3N10-Bcs3$_{E874A}$-His$_6$	185254	AB286/AB287

Supplementary Table 5 | Primers used in this study.

Primer	Sequence (5' to 3')
AB171	GCATCTGGATCCCCTGACATTAAACTCTTCGCG
AB172	GCATCTCTCGAGGTTAAACATCGCAGAAATAGATTC
AB178	AAAGTTTTCAACACATGGGCTGGCGTTGGTTTAAAACATATTGAATTAGCATTAGG
AB179	AAACCAACGCCAGCCCATGTGTTGAAAACTTTGATATTTTCATTCATGTAAACTGG
AB180	ATCGTTAAAGTTGCGCCGTTCATGAAGAAAGATAATTACTTCGCTGAAATG
AB181	TCTTCATGAACGGCGCAACTTTAACGATGAATAGTTGATTTTTATGACGGC
AB189	AGAACTCCATACATTTGCTATTTTTGATACCTTAATTCGCCGCTCGACATTACGTCC
AB190	AATTAAGGTATCAAAAATAGCAAATGTATGGAGTTCTTTTAACGATTTTGGTTGTAATTGG
AB200	TTACTTTATCTCTCGCGCGGGGCATTTCTTAAAGCAAATTGCTGACAAAATTATTGAAATTCG
AB201	TGCTTTAAGAAATGCCCCGCGCGAGAGATAAAGTAAATGGTTTCATAGCCGCGTTTAATTGC
AB202	GGTGGTGGTGGTGGTGCTCGAGTGACTTATCAATATTATTATCTAACTTACC
AB206	ATAACAATAACAACAACAATAACAATAACGTCGACCAAGAAAATGGTTTCGGTAATACCC
AB209	AATTTGCGTTTGTTGCGTTCTGGGGGCGAGGCTATACACAAGATACCTTTGG
AB217	TTTCTTTTGGATTAATTTCTTGTTGAATG
AB224	GGTGGTGGTGGTGGTGCTCGAGTTCAGCAAAACTCACATCTAAATAGAAGCTG
AB228	CTGGAGGTGCTGTTTCAAGGTCCGGTCGACAAAACTTGGTTATTTGGCTCTTATG
AB229	GGTGGTGGTGGTGGTGCTCGAGATTGGTTTTATCATCATTAAATGATTTTTTTACCCAGTC
AB230	GGTGGTGGTGGTGGTGCTCGAGGCCTTTTAACCAATCAACGGCAATAAC
AB231	GGAGGTGCTGTTTCAAGGTCCGGTCGACCAAGAAAATGGTTTCGGTAATACC
AB236	GGTGGTGGTGGTGGTGCTCGAGAGCGTGTTCAAGAAATTTCAAAGT
AB237	CACTTCACCAACAAGGACCATAGCATATGAGAAAAACCCTCACAATTCTCGG
AB246	GGAGGTGCTGTTTCAAGGTCCGGTCGACAAAGATGCAATTAAACGCGGC
AB250	GGAGGTGCTGTTTCAAGGTCCGGTCGACACCAAATATTACAACTATGCTTATG
AB251	GGAGGTGCTGTTTCAAGGTCCGGTCGACGCCTCTTTTGTTCCTTATATCAAC
AB258	GGTGGTGGTGGTGGTGCTCGAGAACAAGCTGTGTGCGGTAACGA
AB268	GGAGGTGCTGTTTCAAGGTCCGGTCGACAAAACTTGGTTATTTGGC
AB269	GGTGGTGGTGGTGGTGCTCGAGTCCAAAGAAATAATCCATAAGATAT
AB270	GGTGGTGGTGGTGGTGCTCGAGCGATTTTGGTTGTAATTGGCAATTA
AB271	GGAGGTGCTGTTTCAAGGTCCGGTCGACGGATTTAAGAAAGAAAATA
AB272	GGTGGTGGTGGTGGTGCTCGAGAACAAGCTGTGTGCGGTAACGAT
AB273	GGAGGTGCTGTTTCAAGGTCCGGTCGACTTAAAAGAACTCCATACAT
AB286	TTCTCATTCTTCGCGCCTATTTTTGCACAAACCCCATACGACAGTATTCC
AB287	TGTGCAAAAATAGGCGCGAAGAATGAGAAGTTTTGTGGTGCTAAGATGAAG
IB036	GAGCTCCAATAACAATAACAACAACAATAACAATAACGTCGACAAAACTTGGTTATTTGGCTCTTATGCTTG
TF142	GCATCTCTCGAGTTTATAGAGATCAGCTAAAATATTGGTAATATATTC
TF143	GCATCTGGATCCAATAAAAATAAAAACATAGGAATCATTCTAGC
TF144	GCATCTGGATCCAATCAATTAGAAATGAAAAAACTCGCC
TF145	GCATCTCTCGAGGTCAATAACTTTGGCACCCTCAG

SUPPLEMENTARY FIGURES

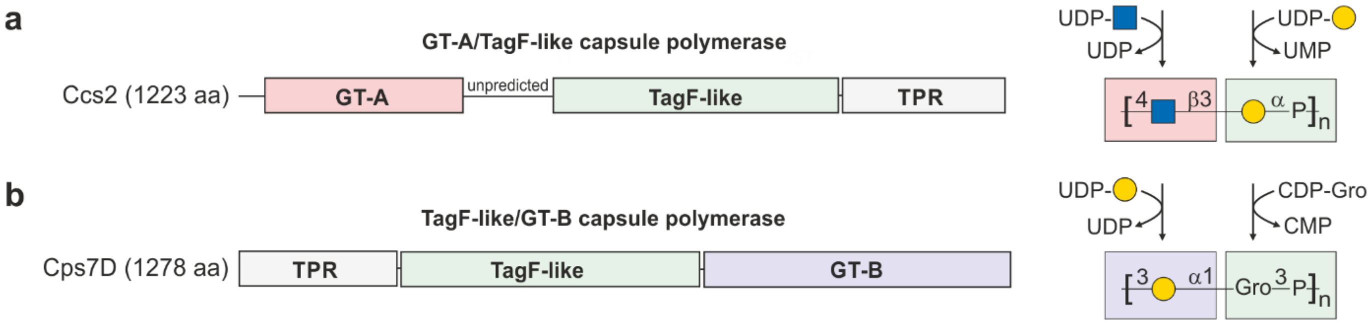

Supplementary Fig. 1 | The TagF-like capsule polymerase family. Domains with similarity to the teichoic acid synthase TagF[8] have been previously identified[9] in ABC-transporter dependent capsule biosynthesis systems, but structural information is missing. **a, b,** Schematic domain organization and catalyzed reactions of (**a**) the GT-A/TagF folded capsule polymerase Ccs2 of *Haemophilus influenzae* type c and (**b**) the TagF/GT-B folded capsule polymerase of *Actinobacillus pleuropneumoniae* serotype 7[9]. The length of each polypeptide in amino acids (aa; indicated in parentheses after each name) is indicated. The majority of polymerases contain a non-catalytic domain rich in tetratricopeptide repeats (TPR). The polymers generated by TagF-like polymerases are linear. Red, violet, and green background colors indicate the domains that transfer the respective moiety shown in the structural representation. A domain with similarity to TagF[8,9] was also identified in Bcs3, whereas GT-A , GT-B, and TPR domains are missing.

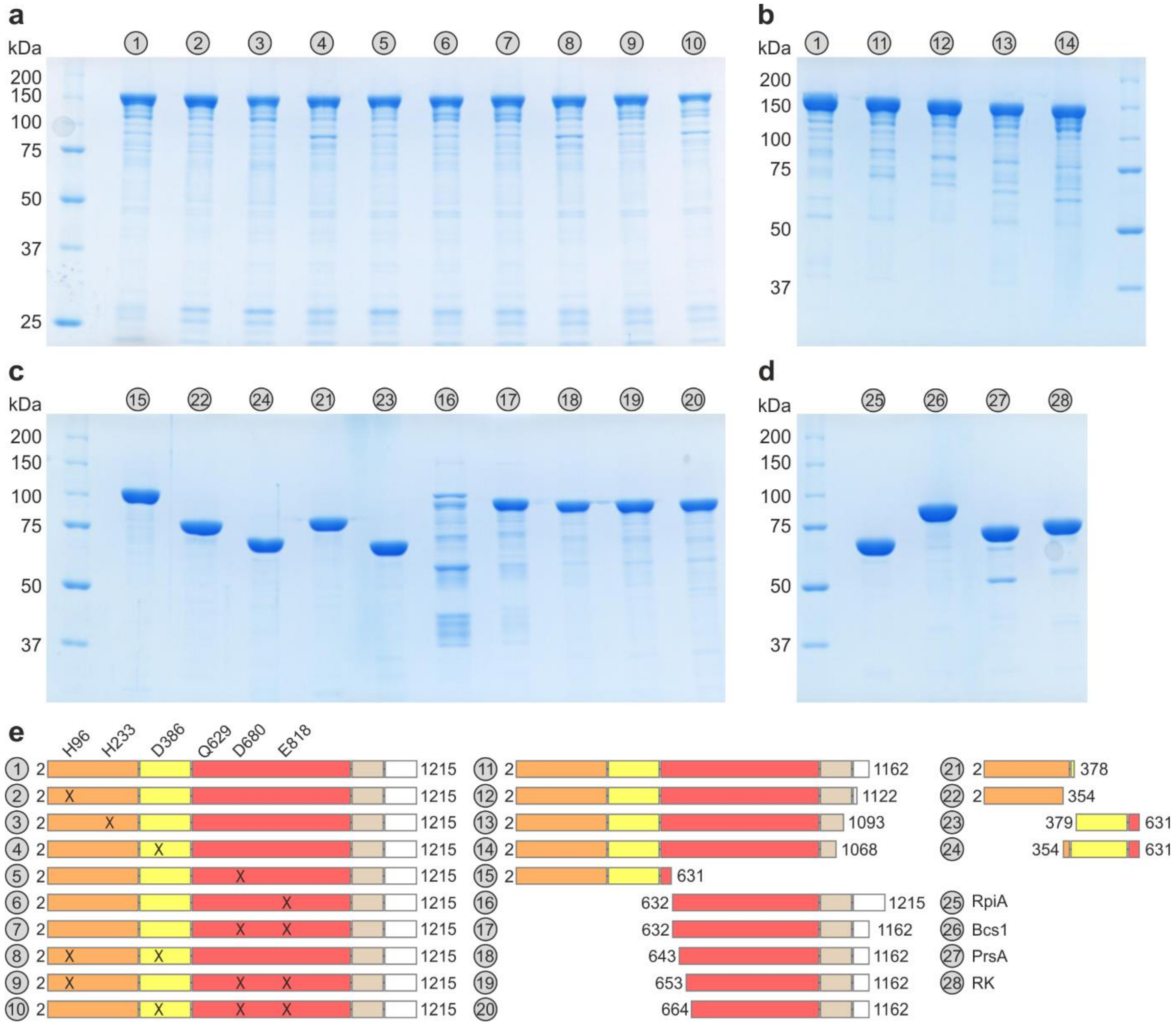

Supplementary Fig. 2 | Coomassie-stained polyacrylamide gels of all constructs (final pools) used in this study (a-d). The schematic representation of the constructs shown in panel **e** is consistent with the representation in Fig. 3. Construct 11 was used for crystallization studies.

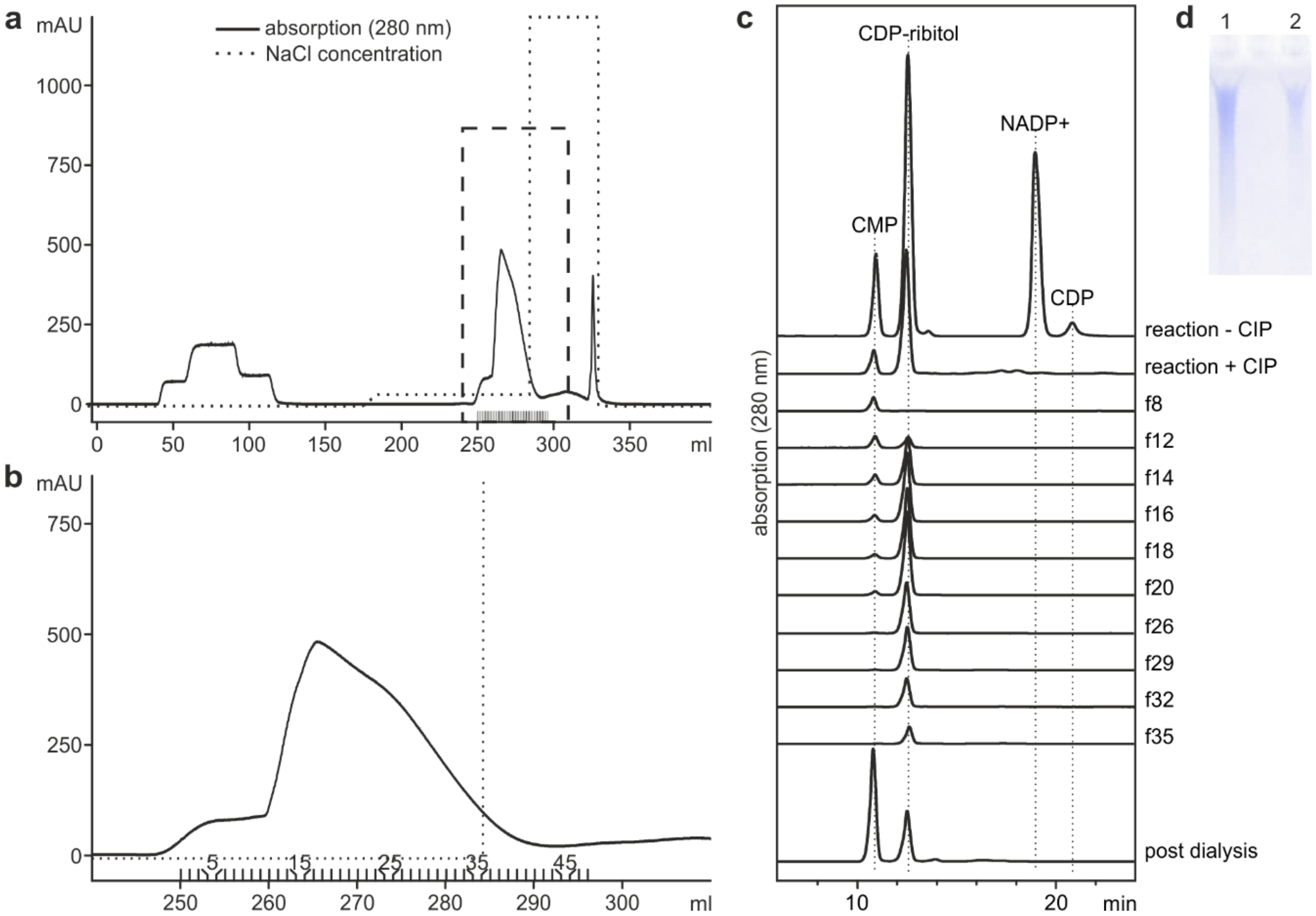

Supplementary Fig. 3 | Purification of CDP-ribitol. A reaction containing RpiA, Bcs1, CTP, NADPH, and ribose-5-phosphate was up-scaled to produce a maximum of 3 mg of CDP-ribitol. After overnight incubation, calf intestinal alkaline phosphatase (CIP) was added to decrease the amount of NADP+ and CMP, which might be co-purified with CDP-ribitol. **a,** The crude reaction was purified by preparative AEC using a DNA Pac PA-100 column (22x250 mm, 22x50 mm Guard, ThermoScientific) and an isocratic gradient of 29 mM sodium chloride. **b,** Zoomed-in section of (**a**) showing the collected fractions. **c,** HPLC-AEC analysis of samples as indicated including the crude reaction mixture +/- CIP. The fractions 26-35, collected after preparative AEC (**b**), were highly pure and devoid of CMP. Fractions 20-45 were pooled and dialysed against water or Tris (pH 7.0) to remove sodium chloride, which ultimately led to the hydrolysis of CDP-ribitol (see post dialysis). **d**, Polymer could be produced using the non-dialyzed, sodium-chloride containing fractions (lane 2) and visualized using Alcian blue stained PA gels. However, the yield was lower if compared to reactions in which CDP-ribitol was supplied *in situ* by RpiA/Bcs1 (lane 1).

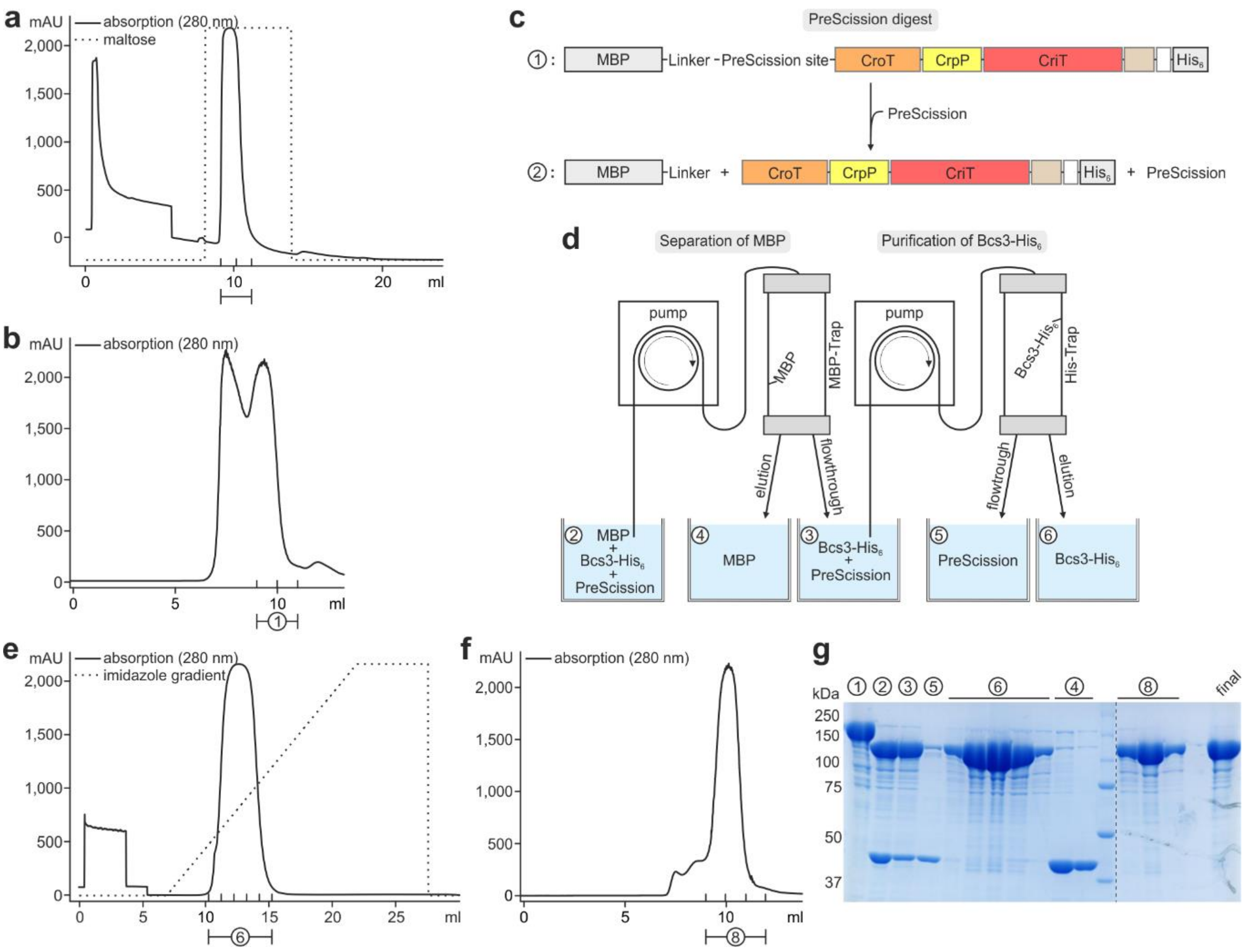

Supplementary Fig. 4 | Purification of the crystallization construct MBP-S3N10-PreScission-Bcs3$_{2\text{-}1162}$-His$_6$. a, b, Chromatograms of the protein purification using (**a**) affinity chromatography (MBPTrap). Indicated fractions were further purified by **(b)** size exclusion chromatography (SEC). **c, d,** Schematic overview of the experimental setup. Removal of the MBP-tag by (**c**) PreScission digest and (**d**) purification of the resulting Bcs3$_{2\text{-}1162}$-His$_6$ (indicated as Bcs3-His$_6$). **e, f,** Chromatograms of Bcs3$_{2\text{-}1162}$-His$_6$ purification using (**e**) affinity chromatography and (**f**) preparative SEC. Bcs3$_{2\text{-}1162}$-His$_6$ (Mw = 137 kDa) elutes with an apparent molecular weight (M_{obs}) of 284 kDa, suggesting the formation of dimers in solution (M_{obs}:Mw = 2.07) after PreScission digest. The dimerization was confirmed by HPLC-SEC. **g,** Coomassie stained SDS-PAGE of protein samples taken during the purification procedure and the final protein used for crystallization. The numbers correspond to the purification stages indicated in panels **a-f**. A dashed line indicates that samples not relevant for data presentation were excised.

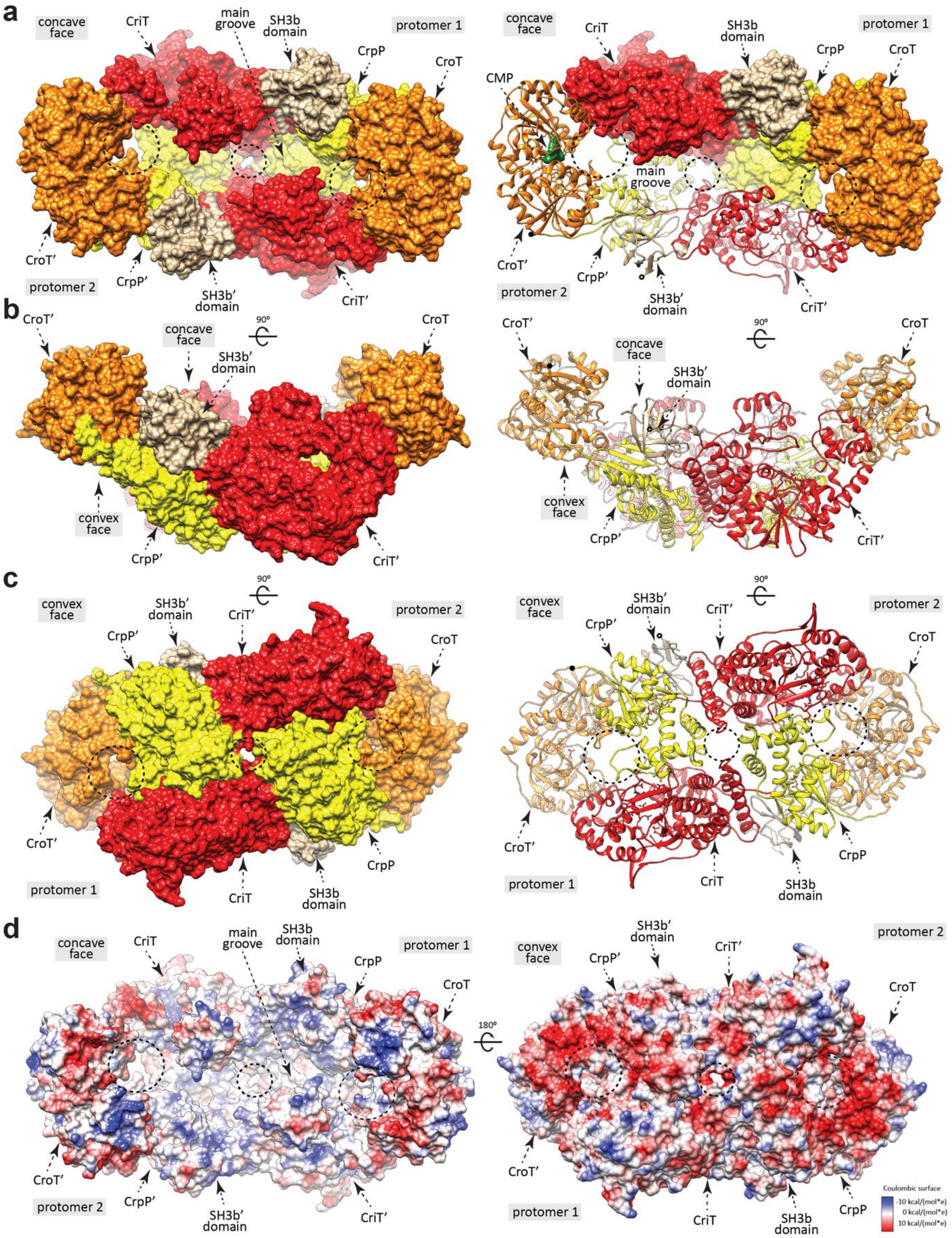

Supplementary Fig. 5 | The overall structure of Bcs3. a, *Left*: Surface representation showing the multi-modular architecture of Bcs3 as observed in the crystal structure of the Bcs3-CMP complex. The Bcs3 protomers build into a physiological dimer, which could be confirmed by size exclusion chromatography (Supplementary Fig. 4f) and which has an overall size of ca. 170 Å × 90 Å × 80 Å (root mean square deviation (RMSD) value between chains of 2.83 Å for 1113 residues; Supplementary Fig. 6). Each protomer of the homodimer is composed of (i) the ribofuranosyltransferase CriT (red), (ii) the phosphatase CrpP (yellow), (iii) the ribitol-phosphate transferase CroT

(orange), and (iv) an SH3b domain (tan). The active sites of the three enzymes in each protomer, as well as each of the SH3b domains and the predicted amphipathic α-helices, face the concave surface of the homodimer. Three pores (dotted circles) connect the concave face with the convex face of Bcs3: (i) each of the two largest pores (ca. 15 Å in diameter) are flanked by CriT from one protomer and CroT' and CrpP' from the neighbor protomer, whereas (ii) a small central pore (ca. 10 Å in diameter) is formed as a consequence of the interactions between the CriTs and the CrpPs of the two protomers. *Right*: One protomer of the Bcs3 homodimer is shown as surface representation, whereas a second protomer is shown as cartoon representation, showing the general fold and secondary structure organization of Bcs3. The location of CMP in the CroT active site is shown in green. **b,** *Left*: Surface representation showing the concave and convex faces of Bcs3 as observed in the crystal structure of the Bcs3-CMP complex. *Right*: Cartoon representation showing the concave and convex faces of Bcs3, in a similar orientation. **c**, *Left*: Surface representation showing the convex face of Bcs3 as observed in the crystal structure of the Bcs3-CMP complex. *Right*: Cartoon representation showing the convex face of Bcs3, in a similar orientation. **d,** Two views of the coulombic charge surface representation of Bcs3 showing the concave and convex sides. *Left*: Note that the concave face presents an overall surface with scattered patches of positively charged and neutral residues, whereas the convex face (*right*) presents a predominantly negatively charged surface.

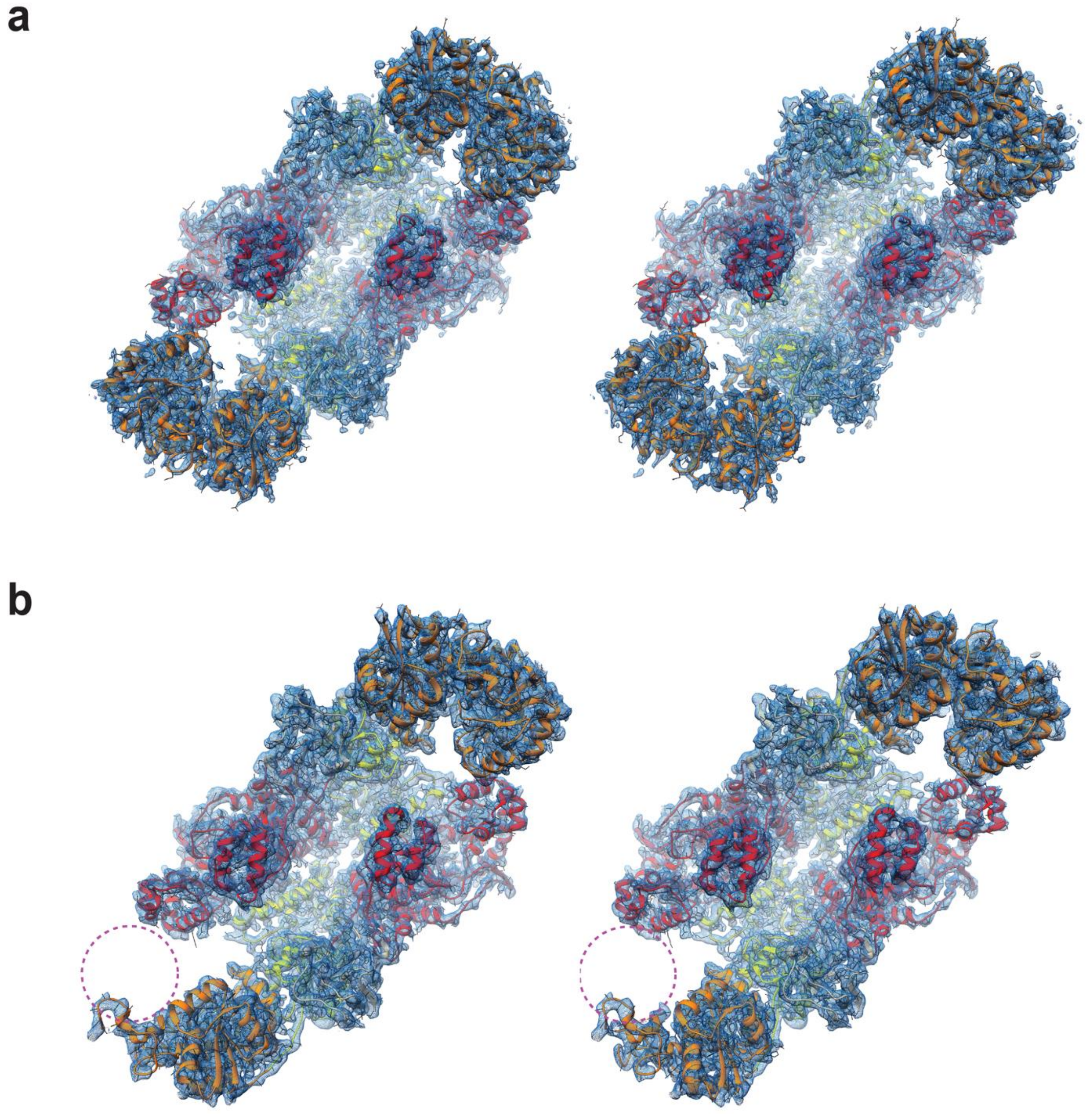

Supplementary Fig. 6 | Stereo views of maps for the Bcs3 dimers. a, Overall density map of Bcs3 in complex with CMP displayed at 1 RMSD. **b,** Overall density map of a selected dimer of Bcs3 in complex with polymer displayed at 1 RMSD. The CroT enzyme present in one of the protomers contains an unmodelled region corresponding to the C-terminal Rossmann-fold (dashed circle) and indicating flexibility of this region in the crystal structure.

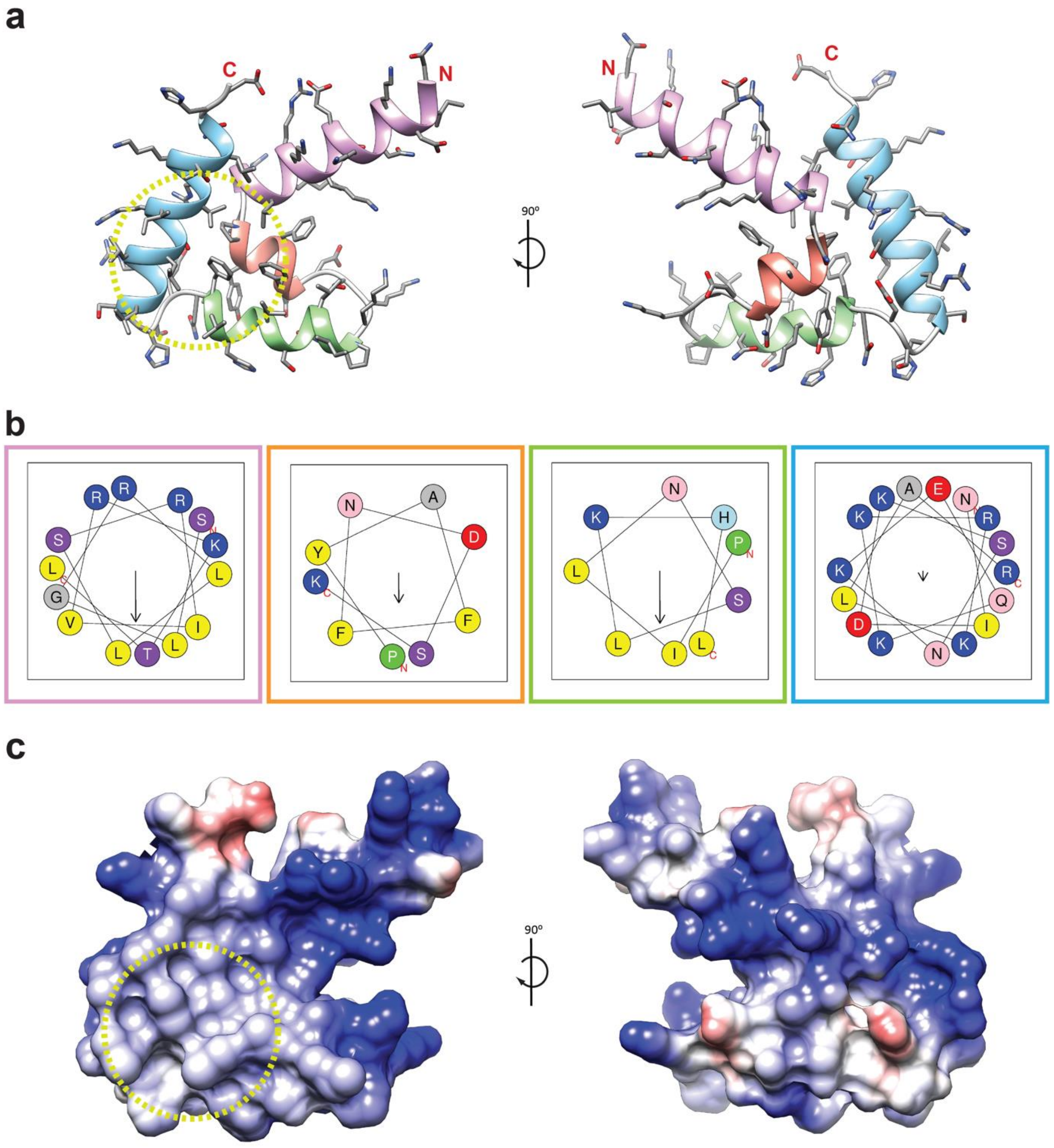

Supplementary Fig. 7 | Predicted structure of the C-terminal region of Bcs3. The C-terminal region (1116-1215) of Bcs3 was not visible in the crystal structure and was thus subjected to fold modeling using Alphafold Colab[10,11]. The model did not provide a secondary structure for the first 52 N-terminal amino acid residues (coil; not shown). However, the C-terminal 48 residues are predicted to adopt an α-helical bundle (CT-HB). **a,** The predicted CT-HB is shown in two orientations with its four α-helices indicated by colors: α56 (pink), α57 (orange), α58 (green), and α59 (light blue). The N- and C-terminus are indicated as well (red font). Note that the central region of the predicted cluster is rich in aromatic and aliphatic residues (yellow dashed circle). **b,** Helical wheel projections of the four corresponding helices generated using the heliQuest server[12] (https://heliquest.ipmc.cnrs.fr/). The frame color corresponds to the helix color used in **a**. Residues are colored by their group physicochemical characteristics in non-polar (ILFVY - yellow), small (AG - grey), proline (P - green), small polar (ST - purple), positively charged (KR -

blue), acidic (DE - red), polar (NQ - pink), and histidine (H - light blue). N- and C-terminal residues are indicated as red subscript. Central arrows are proportional to the hydrophobic moment of the helix. Note that α56, α57, and α58 are the main contributors to the amphipathic character of CT-HB. **c,** Surface representation with coulombic charge coloring of the CT-HB as shown in **a**. Note the predicted hydrophobic patch on one side of the CT-HB (yellow dashed circle).

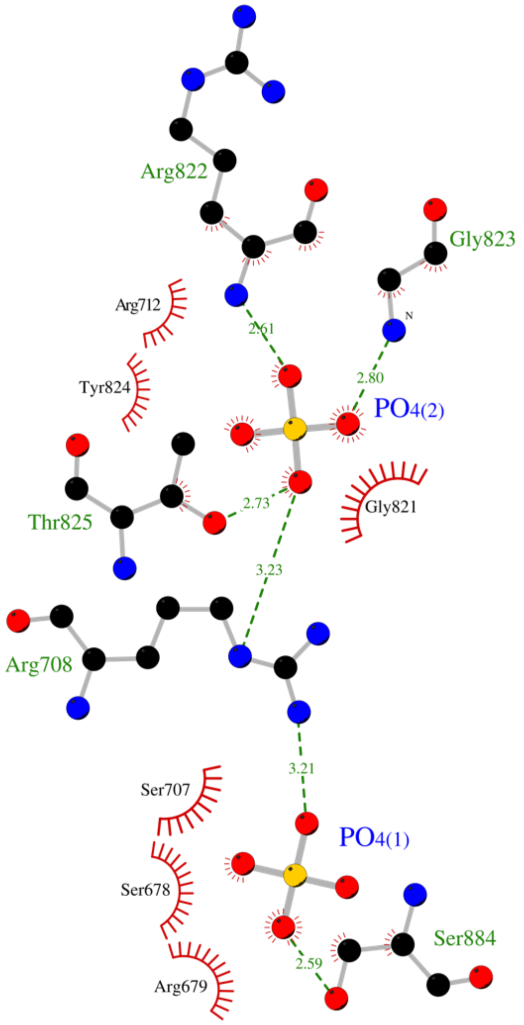

Supplementary Fig. 8 | CriT active site environment and phosphate binding sites. 2D representation of the two phosphates interacting with active site residues, generated with Ligplot+[13]. Interactions between Bcs3 and these phosphates include several residues. Specifically, $PO4_1$ interacts with R679; the backbone carbonyl group from S678; the side chains of S707; and it forms a hydrogen bond with S884; all residues contribute to the formation of the $PO4_1$ binding cavity. Additionally, the side chains of D680 and E818 are located close to $PO4_1$. For $PO4_2$, a hydrogen bond is formed with the backbone amide group at position R822; the side chain of R708 Nε makes electrostatic interactions; the backbone amide group at position G823 makes a hydrogen bond; the side chains R712 and side-chain T825 make electrostatic interactions with $PO4_2$. Interestingly, the negatively charged oxygens of the $PO4_1$ and $PO4_2$ groups are anchored at the amino terminus of the α45, and the α37 and α38, respectively, where they are apparently stabilized by the positively charged helix dipole.

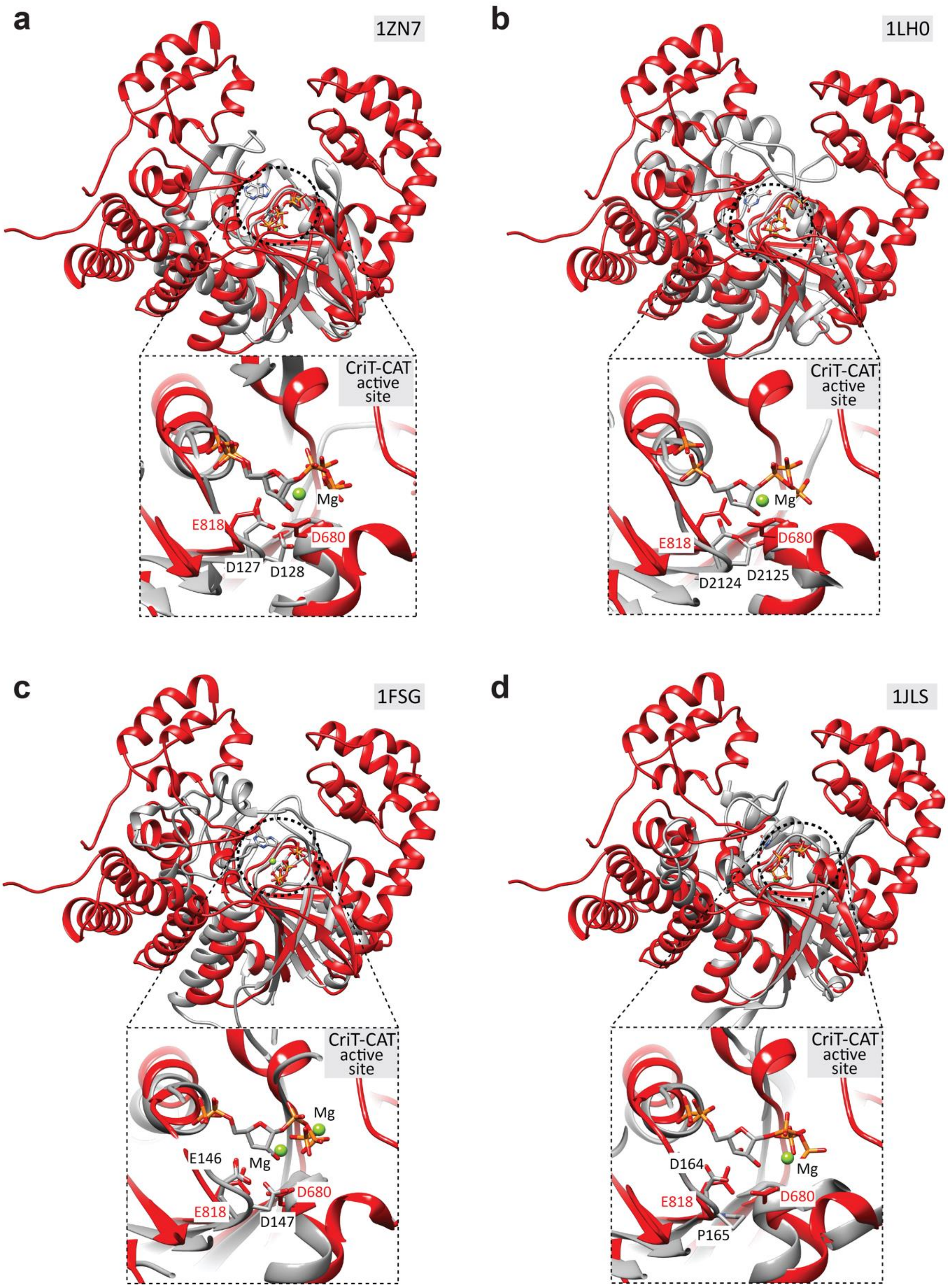

Supplementary Fig. 9 | Structural comparison of CriT and PRTase homologs in complex with PRPP-Mg^{2+}. Gallery of one-to-one structural superposition of CriT (in red) with PRTase homologs (in grey) demonstrating the similarity of the core Rossmann-fold domain. The PDP codes of the PRTase homologs complexed with PRPP and Mg^{2+} are indicated in the top left corner of each panel: **a,** 1FSG[14], *T. gondii* hypoxanthine-guanine phosphoribosyltransferase (HPRT); **b,** 1ZN7[15], human adenine phosphoribosyltransferase (APRT); **c,** 1LH0[16], *S.*

typhimurium orotate phosphoribosyltransferase (OPRT); and **d,** 1JLS[17], *T. gondii* uracil phosphoribosyltransferase (UGRT) mutant C128V. The active site (dashed circle) and the close-up (dashed square) show the binding of PRPP in complex with Mg^{2+} in a consensus conformation that was used for guided docking by homology in CriT. The docking is supported by the two phosphates found in the CriT active site, which correspond to the 5-phosphate and the beta-phosphate of PRPP in the homologs. Moreover, CriT residues E818 and D680 labeled in red show co-localization with the corresponding DD motif of PRTases, which are involved in the positioning of PRPP at the homologs' active sites.

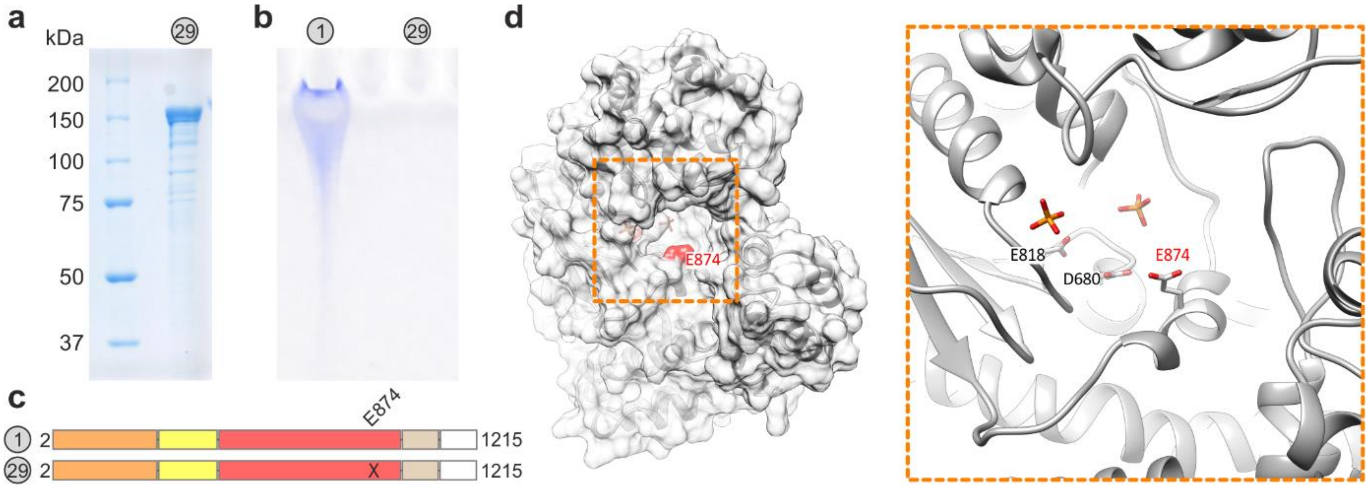

Supplementary Fig. 10 | E874 of CriT is a promising candidate for the putative base that activates 1-OH of the non-reducing end ribitol for the nucleophilic attack on ribose C1 of PRPP. See also Supplementary Fig. 11a. **a,** SDS-page shows purified Bcs3$_{E874A}$ (construct 29). **b,** Alcian blue stained PA gel demonstrating that polymeric products are absent in a reaction containing Bcs3$_{E874A}$. Bcs3 was used as control. **c,** Schematic representation of Bcs3 (construct 1, see Fig. 3b) and Bcs3$_{E874A}$ (construct 29). **d,** *Left*: Surface representation of CriT showing the exposed residue E874 depicted as a red-colored protrusion at the catalytic site entrance. The location is close to the reaction center alongside the putative axis for the entrance of the polymer, making it the preferred candidate for assisting the deprotonation of the attacking 1-OH of the non-reducing end ribitol of the nascent chain. *Right*: Close-up of the CriT active site, showing the position of E874 in relation to E818, D680, and the two phosphates found at the active site.

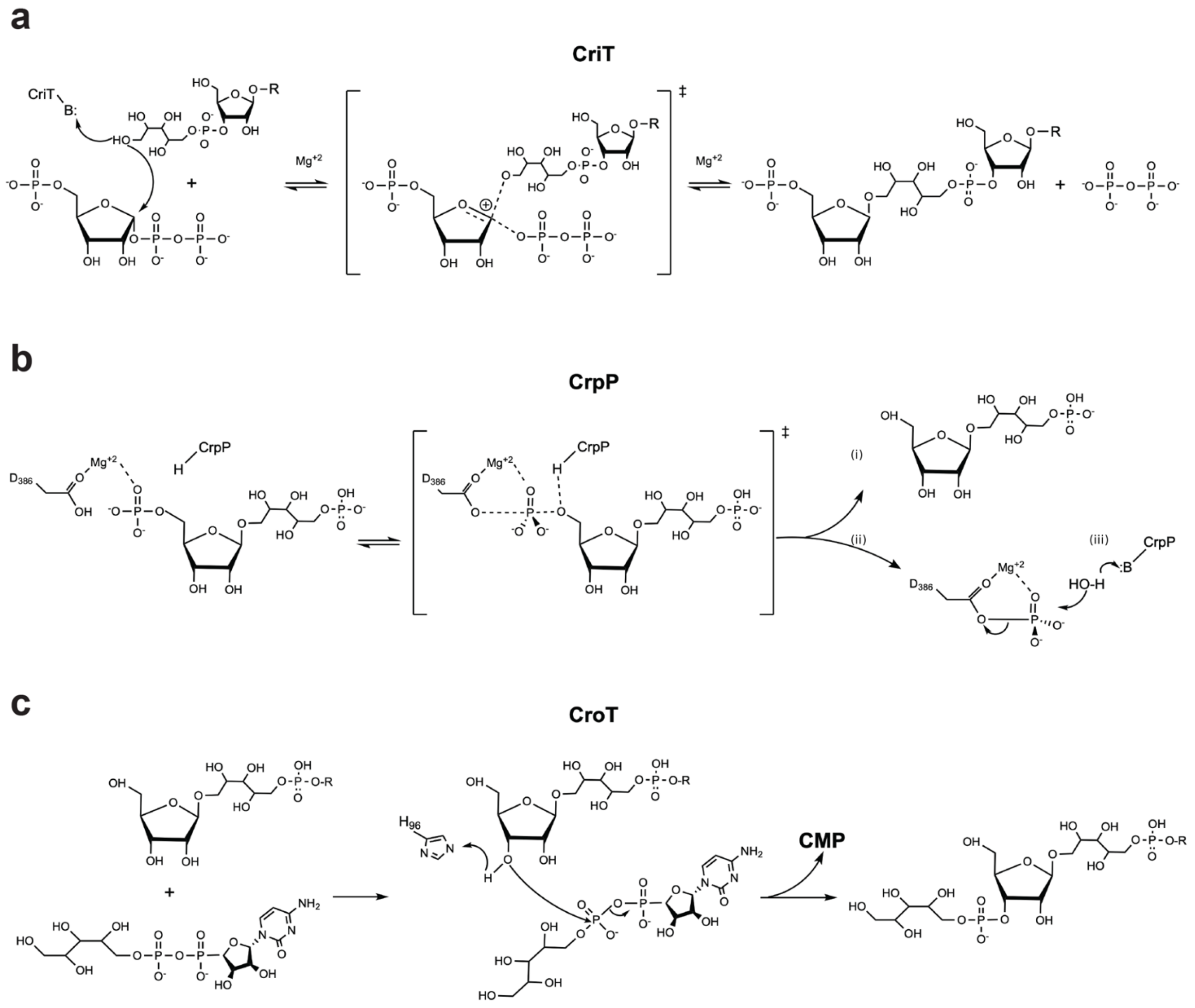

Supplementary Fig. 11 | Overview of the putative catalytic mechanisms utilized by CroT, CrpP, and CriT. a, Proposed mechanism for CriT: Studies performed with homologous phosphoribosyltransferases (PRTases) involved in nucleotide synthesis support that the 5-phosphoribosyl group is transferred from PRPP in a Mg^{2+}-dependent reaction involving an oxocarbenium transition state at ribose C1 formed as a result of a nucleophilic attack. To allow this nucleophilic attack to happen, the acceptor nitrogen from the nucleobase ring is activated by a base. A nucleoside monophosphate and pyrophosphate are the products of this reaction[14–21]. Based on homology, we propose that the mechanism for CriT requires the presence of Mg^{2+} and is initiated by a base (B:) abstracting a proton from O1 of the ribitol moiety at the growing extreme of the polymer. We suggest this base to be E874, which is located at the active site entrance and for which mutation to alanine leads to inactive enzyme (Supplementary Fig. 10). The ribitol O1 then engages in a nucleophilic attack on the C1 of PRPP, resulting in the release of pyrophosphate, the formation of a glycosidic linkage and the inversion of stereochemistry at the anomeric carbon C1 of the ribose, finally yielding a polymer terminus that is elongated by ribofuranose-5-phosphate. **b,** The substrate for CrpP is the terminal ribofuranose-5-phosphate released by CriT (**a**). According to homologous HAD phosphatases[22–27], we propose that the 5-phosphate in the active site of CrpP coordinates with Mg^{2+}, which is held in place by the catalytic residue D386.

A pentavalent transition state is formed (central panel). Cleavage of the phosphoester bond is achieved by protonating O5 of the ribose, involving a yet to be identified acid and leading to the release of the polymer extreme (i). The resulting covalent phosphoaspartyl-enzyme intermediate (ii) is hydrolyzed by the action of a water molecule activated by a yet to be identified base (B:), a required step to regenerate D386 and to release phosphate (iii). **c,** The proposed mechanism utilized by CroT comprises the binding of CDP-ribitol in the active site and the entry of the terminal ribose of the polymer (the product of CrpP). In analogy to what is proposed for the homologous glycerol-phosphate polymerase TagF[8,28,29], the transfer of the ribitol-phosphate moiety to the terminal ribosyl acceptor is initiated by the deprotonation of the ribose 3-hydroxyl group, catalyzed by the base H96. This allows a nucleophilic attack of ribose O3 towards the β-phosphate of CDP-ribitol (central panel). Possibly through the formation of a pentavalent transition state, CMP is released, and a new phosphoester is formed, yielding a polymer terminus elongated by a ribitol-phosphate moiety.

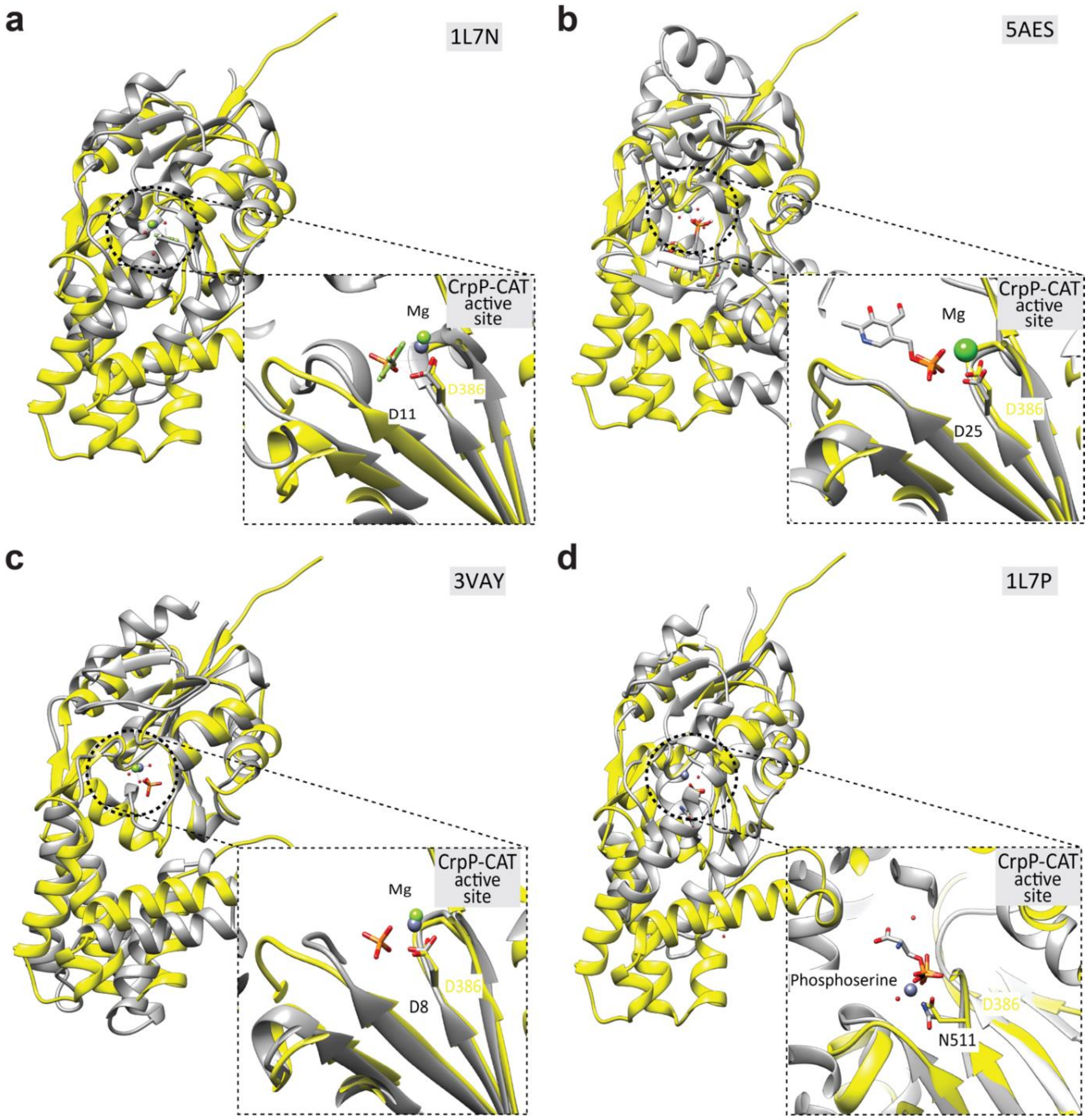

Supplementary Fig. 12 | Structural comparison of CrpP and HAD phosphatase homologs. Gallery of one-to-one structural superposition of CrpP (in yellow) with homologs (in grey) displaying the similarity between the catalytic Rossmann fold domains. The PDB codes of the homologs in complex with different ligands and divalent atoms are depicted in the top left corner of each panel: **a,** 1L7N[30], Phosphoserine Phosphatase (PSP) with substrate transition state analog; **b,** 5AES[25], the structure of murine Pyridoxal Phosphate Phosphatase (PDXP) in complex with a pyridoxal phosphate analog inhibitor; **c,** 3VAY[31], the structure of *P. syringae* 2-haloacid dehalogenase; and **d,** 1L7P[30], the structure of PSP mutant N511D in complex with substrate phosphoserine. A dashed circle highlights the active site and a dashed square displays a close-up showing the phosphate binding site with substrates/analogs, and the preserved position for the divalent metal ion, which is required for activity in HAD-like enzymes[22,30]. PDXP and PSP ligand conformation was used for guided docking by homology of the substrate in CrpP, using the phosphate and the Zn^{2+} (in Bcs3-CMP complex) or Mg^{2+} (in Bcs3-DP2 complex) found in the active site as reference.

Besides the structural motifs, HAD-like phosphatases are defined by the presence of four sequence motifs I-IV[26]. Motif I is a signature DXD located at the end of the first β-strand, right at the beginning of loop 1 (Fig. 4). Only the first aspartate (D386) of this motif is conserved in CrpP and was shown to form the aspartyl-phosphate intermediate in HAD-like enzymes[26]. D386 superimposes well with the corresponding residues in the homologs (**a-d**). Important residues from motifs II-IV are shown in Fig. 4d. Motif II is characterized by a highly conserved threonine or a serine (S513 in CrpP-CAT) that is located at the end of the second β-strand. Motif III corresponds to a conserved lysine (K548 in loop 3 of CrpP-CAT) and motif IV correspond to a conserved acidic residue located at the end of the fourth β-strand (D573, loop 4, β18, in CrpP). Both motifs II and III contribute to the stability of the reaction intermediates of the hydrolysis reaction in HAD-like enzymes[26].

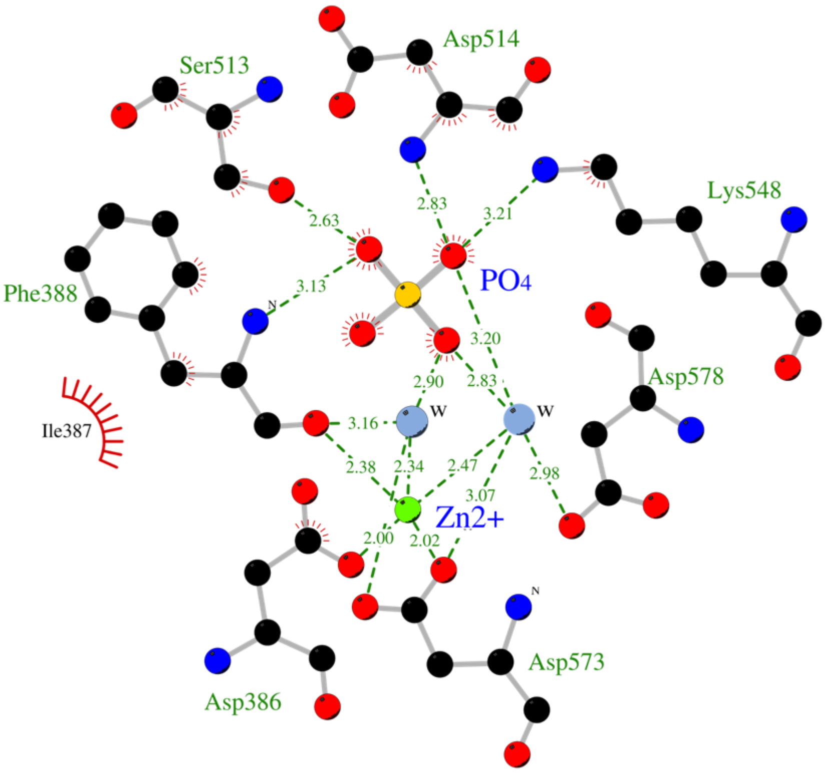

Supplementary Fig. 13 | CrpP active site environment and phosphate and metal binding site. 2D representation of the interaction of the phosphate and the metal (Zn^{2+}, coordinating waters marked as w) in the active site of CrpP. The figure was generated with Ligplot+[13]. Several residues are involved. Specifically, the $PO4_1$ interacts with the amide backbone from residue D514; makes a hydrogen bond with the side chain of S513 (motif II, see Supplementary Fig. 12, figure legend); makes hydrogen bonds with the side chain of D386 and the backbone amide group at position F388; makes a hydrogen bond with the side chain of K548 (motif III). Moreover, the $PO4_1$ is coordinated by the Zn^{2+}, which, in turn, is coordinated by the side chain of D573 (motif IV), D386 (motif I), and the backbone carbonyl group at position F388.

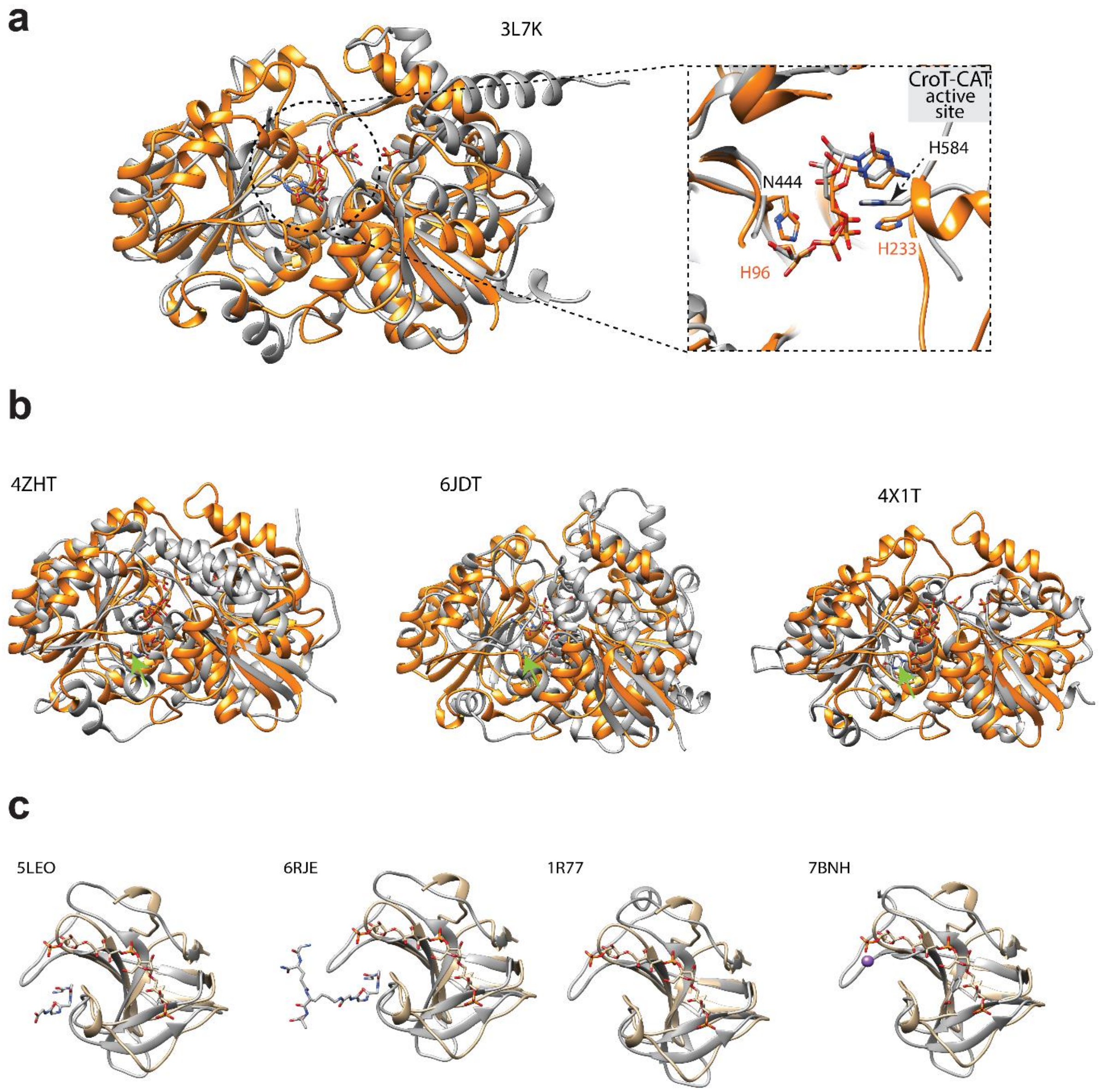

Supplementary Fig. 14 | Structural comparison of CroT and SH3b domains with homologs. a, Structural comparison of CroT and its closest structural homolog, the wall teichoic acid polymerase TagF[8]. Specifically, a superposition of CroT in complex with CMP and glycerol (in orange), and the TagF mutant H444N in complex with CDP-glycerol (in grey, PDB code: 3L7K[8]) is presented. Overall, the Rossmann fold domains of CroT and TagF superimpose well. Furthermore, the active sites (dashed circle and dashed square for the close-up) display the same overall orientation of CDP-glycerol in TagF and of CMP and glycerol in CroT, assisting and validating the docking of CDP-ribitol in Bcs3. Note the equivalent positioning of the pair of histidine residues at the active site (H96 and H233 of CroT labeled in orange, superimposing with N444 (H444 in the wildtype) and H584 of TagF labeled in grey, respectively). **b,** A gallery of one-to-one superpositions of CroT with other GT-B fold homologs (in grey) in complex with nucleotide substrates (position indicated by green arrows). From left to right: 4ZHT, the structure of *H. sapiens* UDP-GlcNAc 2-epimerase in complex with UDP[32]; 6JTD, *T. chinensis* C-glycosyltransferase in complex with CMP[33]; and 4X1T, *A. thaliana* galactolipid synthase in complex with UDP[34]. The above presented comparison supports that CroT display strong similarities with NDP-sugar-dependent GT-B fold enzymes, even though GT

generate glycosidic bonds, whereas CroT creates phosphodiester linkages. Thus, CroT likely functions through an open-to-close mechanism, which is commonly found during substrate binding and catalysis of GT-B fold family enzymes[35–37], including TagF[8], MurG[38], glycogen synthase[39], PimA[40–44], and MshA[45]. This hypothesis is supported by the inter-domain movement observed in CroT and shown in Extended Data Fig. 6b and Supplementary Fig. 16. In the case of GT-B enzymes, catalysis occurs through two mutually excluding mechanisms involving the anomeric carbon: (i) inverting GTs proceed via a S_N2 reaction in a single displacement mechanism that includes a residue acting as a general base catalyst, which activates the acceptor substrate and the formation of an oxocarbenium ion-like transition state, while (ii) retaining GTs follow a front-side substrate-assisted S_Ni-type reaction, a two-step reaction which involves the formation of an oxocarbenium ion transition state[41,46,47]. However, similar to TagF[8], we propose a simple displacement mechanism for CroT (Supplementary Fig. 11c). **c,** Gallery of one-to-one superpositions of the SH3b domain in complex with DP2 (in tan) and SH3b domain homologs (in grey), demonstrating a conserved fold. PDB code: 5LEO, the structure of the lysostaphin SH3b domain with peptidoglycan fragment; 6RJE, *S. aureus* lysostaphin SH3b in complex with peptidoglycan[48]; 1R77, the structure of the *S. capitis* cell wall targeting domain of peptidoglycan hydrolase ALE-1[49]; and 7BNH, *S. simulans* lysostaphin SH3b domain structure. SH3b domains are thought to bind the receptors of their target cells or utilize the SH3-like modulating pathways to promote pathogen survival in the invaded cells[50]. Besides the above presented structural comparison, a BLAST search of the SH3b domain of Bcs3 indicated that it belongs to the DUF5776 superfamily (Pfam 19087). Homologues with identities between 30-70 % were found in Gram-negative pathogens of genera *Pasteurella*, *Mannheimia*, *Actinobacillus*, *Acinetobacter*, *Castellaniella*, and *Klebsiella* and in Gram-positive pathogens of genera *Lactococcus*, *Enterococcus*, *Weissella*, and *Mammaliicoccus*. The amino acids $_{1094}$GXPRL$_{1098}$ and $_{1103}$GYITA$_{1107}$ are described to be especially conserved according to the conserved domains database[51]. Interestingly, the majority of homologues identified through BLAST searches using the entire Bcs3 sequence as query also contained the SH3b domain (Supplementary Data 1, conserved SH3b motifs highlighted in blue).

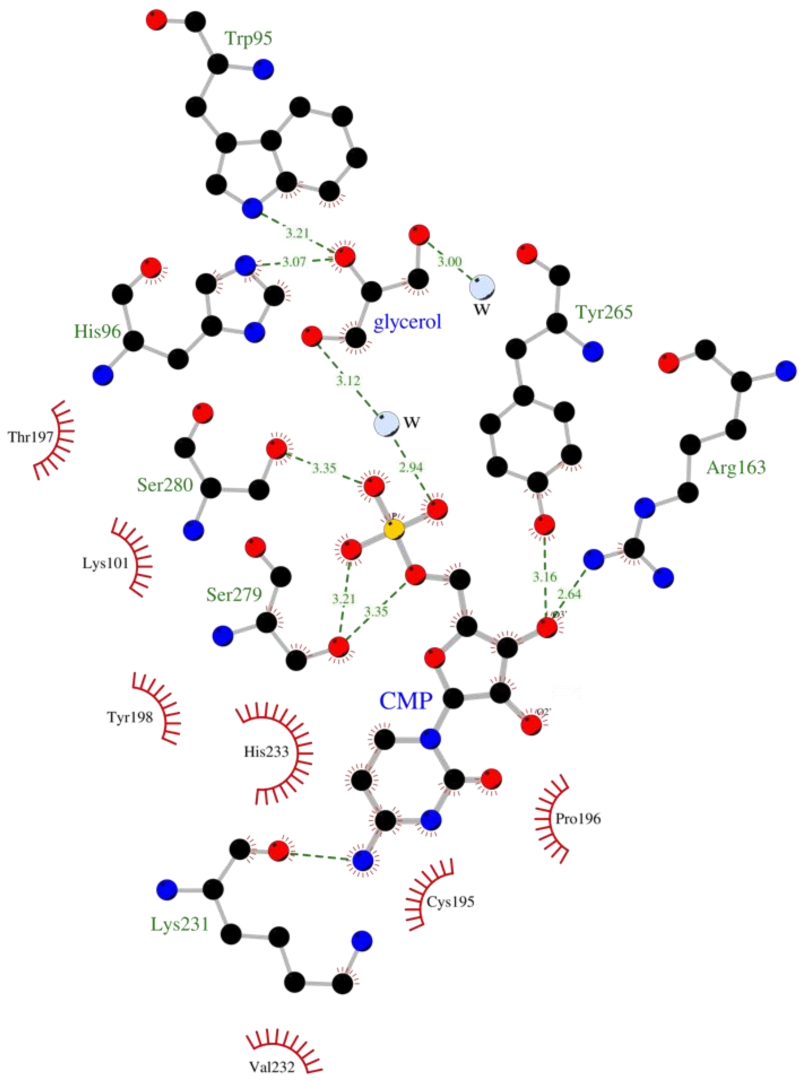

Supplementary Fig. 15 | CroT active site environment and CMP and glycerol binding sites. 2D representation of CMP and glycerol interacting with active site residues of CroT. The figure was generated with Ligplot+[13]. w: water. The following interactions of the CMP and the glycerol could be observed: The backbone carbonyl groups at positions P196 and V232 make hydrogen bonds with N4 of the cytosine heterocycle; K231 makes an interaction with the cytosine ring. The side chains of Y265 make hydrogen bonds with O2 of CMP; and the side chain of S280 with the αPO4 of CMP. The side chain of R163 makes electrostatic interactions with O3 of ribose. H96 makes a hydrogen bond with O2 of the glycerol. Finally, αPO4 interacts with the side chains of T197 and makes a hydrogen bond with S279.

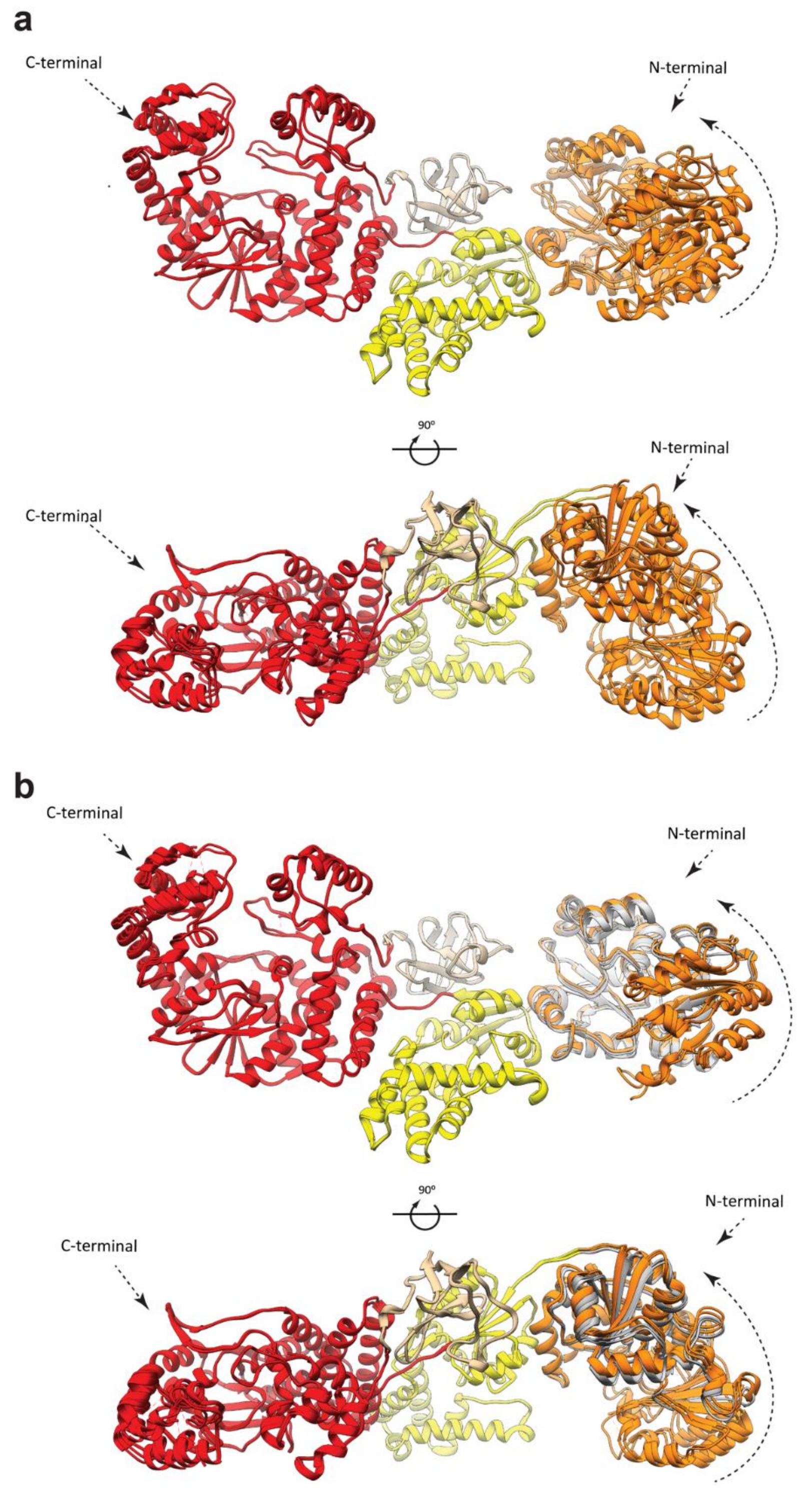

Supplementary Fig. 16 | Structural superposition of protomer chains found in the Bcs3-CMP and Bcs3-DP2 crystal structures. Protomer chains are depicted as ribbons representations using the above established color code: CroT (orange), CrpP (yellow), CriT (red), and SH3b domain (tan). **a,** *Top*: Structural superposition of two protomers of the dimer in the crystal structure of Bcs3-CMP, together with (*bottom*) a 90º rotation of the same superposition. **b,** *Top*: Structural superposition of four protomer chains, corresponding to two dimers found in the Bcs3-DP2 structure and two dimers found in the Bcs3-CMP structure. *Bottom*: A 90º rotation of the same superposition. Note that two chains belonging to the two different protomers of Bcs3-DP2 have partially modeled CroT domains (in

grey). The dashed arrow indicates displacements of the CroT domain between chains for both superpositions. Altogether, both crystal structures show that CroT displays higher mobility than CriT and CrpP in the dimeric ensemble, likely reflecting that CroT undergoes conformational changes during catalysis. A similar inter-domain movement has been proposed for other members of the GT-B superfamily during substrate binding and catalysis including TagF[8], MurG[38], glycogen synthase[39], PimA[40–44], and MshA[45].

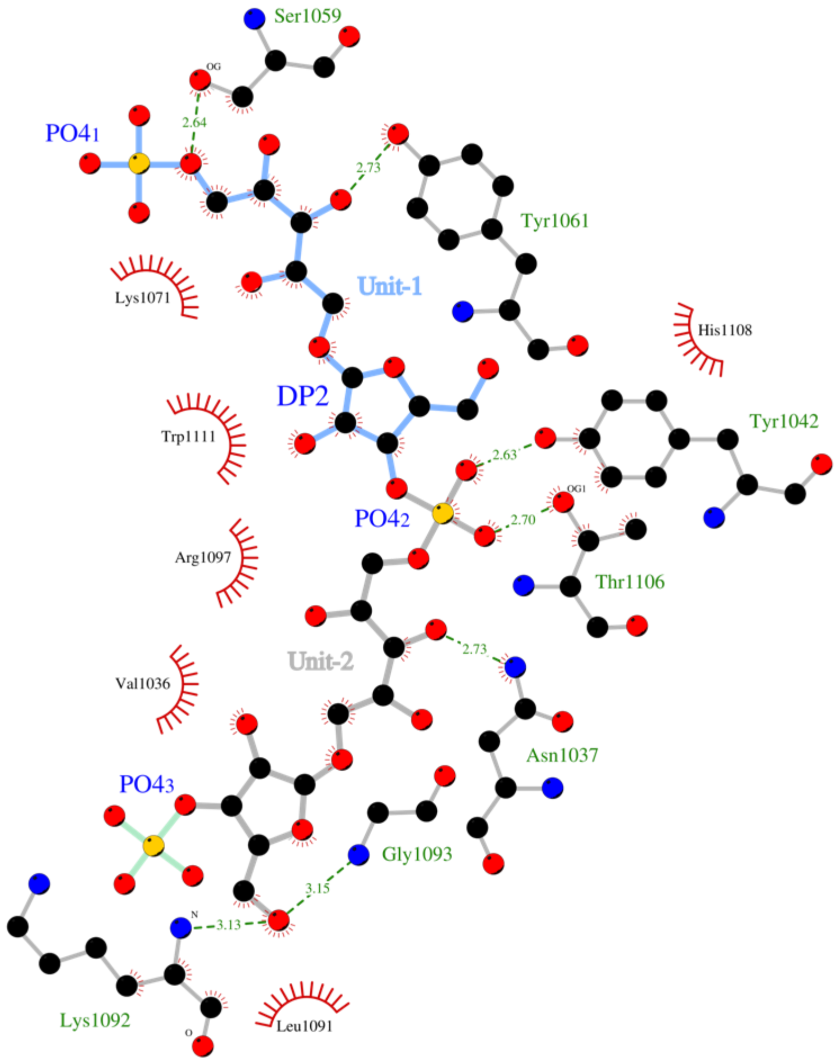

Supplementary Fig. 17 | DP2 binding site. 2D representation of interactions between the SH3b domain and DP2, generated with Ligplot+[13]. Several residues are involved. Specifically, the backbone at positions K1092 and G1093 interact with O5 of the ribose ring of the second repeating unit of DP2. Moreover, the side chain of N1037 (Fig. 6e, loop 4) makes a hydrogen bond with the C3 OH group of the ribitol, whereas the side chain of R1097 makes additional interactions with the ribitol (Fig. 6e). In addition, the $PO4_2$ group attached to the second repeating unit makes a hydrogen bond with the side chain of Y1042, interacts with the side chain of H1108, and also makes a hydrogen bond with the side chain of T1106. The side chain of W1111 stabilizes the ribose ring of the first repeating unit of the DP2 molecule. The side chain of Y1061 makes hydrogen bonds with the C3 OH groups of ribitol. Finally, the side chains of S1059 and K1071 anchor the $PO4_1$ group attached to position 5 of the ribitol moiety in the first repeating unit. Importantly, the non-reducing end of DP2 (repeating unit 2), where elongation takes place in ABC-transporter dependent (group 2) assembly systems, points towards the main grove of the Bcs3 dimer, facing the catalytic centers of the complex (Fig. 6a, b).

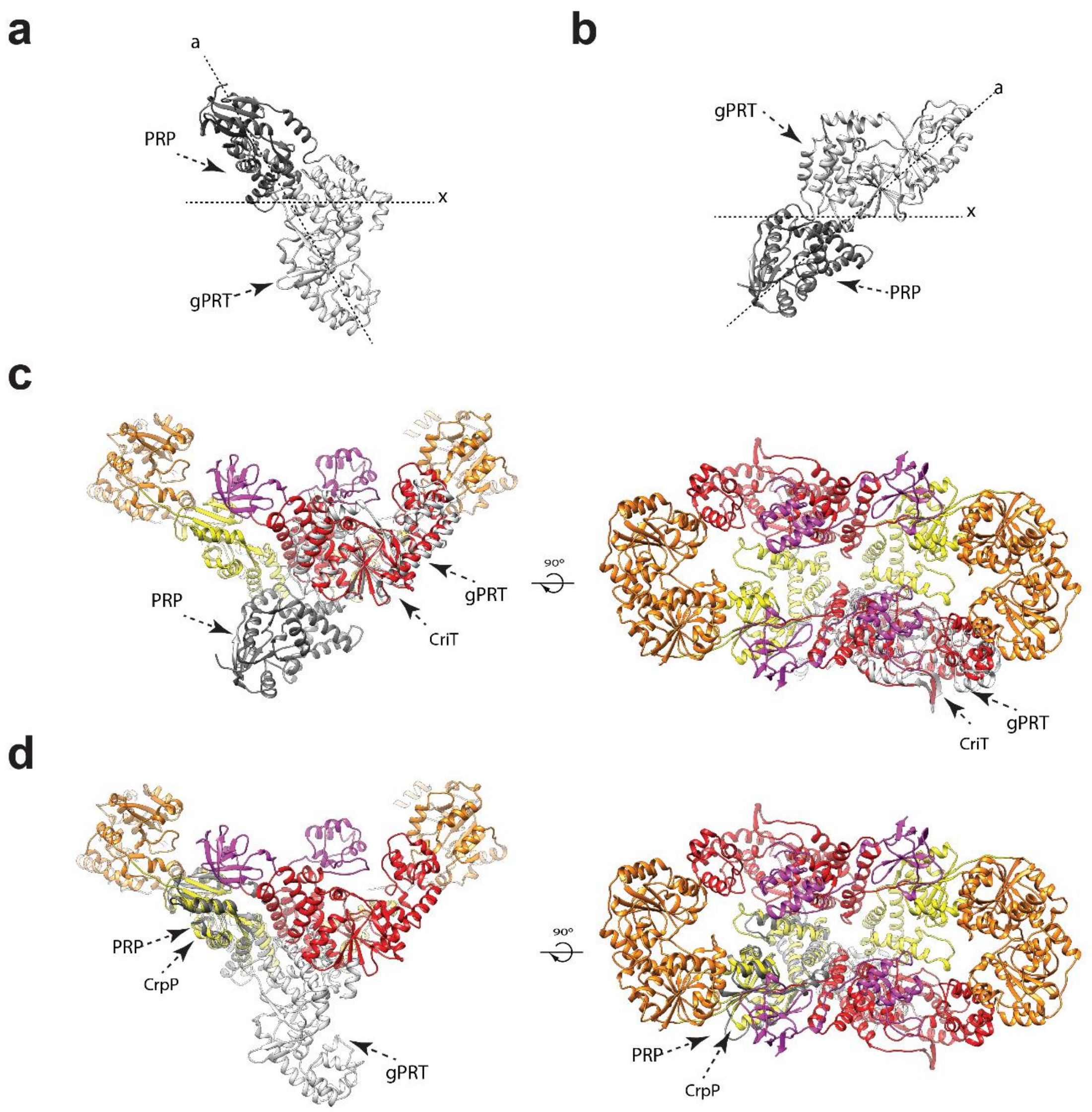

Supplementary Fig. 18 | Structural comparison of CriT and CrpP from Bcs3 with the HAD-like phosphatase and the putative ribofuranosyltransferase from *T. composti*. a, b, Structure of the monomeric *T. composti* enzyme (PDB code: 7SHG[52]) with the phosphoribosyl-phosphatase (PRP) domain (homolog to CrpP) colored in dark grey, and the putative phosphoribosyltransferase (gPRT) domain (homolog to CriT) colored in light grey. **c,** The *T. composti* enzyme has the same orientation as in **b**, but is shown as a superposition with CriT from one Bcs3 protomer. A 90º rotation of the same superposition is shown as well. **d,** The *T. composti* enzyme has the same orientation as in **a**, but is shown as a superposition with CrpP from one Bcs3 protomer. A 90º rotation of the same superposition is shown as well. The fact that only one domain of the *T. composti* enzyme can be aligned with CrpP and CriT of Bcs3 demonstrates that the PRP and gPRT domains of the *T. composti* enzyme have a different orientation to each other than CrpP and CriT in Bcs3. As a consequence, also the active sites in the PRP-gPRT are oriented differently. Interestingly, the *T. composti* enzyme only showed activity for the PRP domain and the structure was obtained using a C-terminally truncated enzyme (UNIPROT code: L0EJI9). A sequence alignment of Bcs3 and the *T. composti* enzyme indicates that the truncated region is similar in length and location to the amino acid sequence harboring the HB-3 of CriT and the SH3b domain (colored in magenta in **a** and **b**). This suggests that both HB-3 and SH3b might have a role in orienting CrpP and CriT in the Bcs3 dimer.

Supplementary Figure 2a

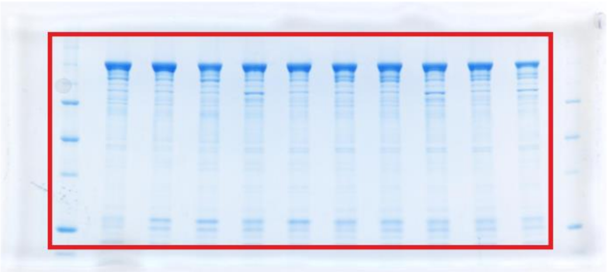

Supplementary Figure 2b (red) and 2d (green)

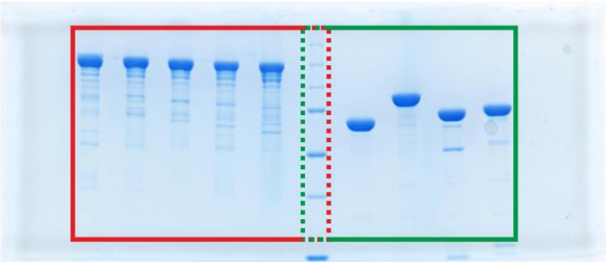

Supplementary Figure 2c

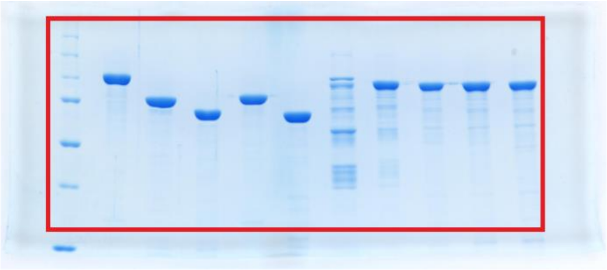

Supplementary Fig. 19 | Source Data for Supplementary Figure 2. Unprocessed gels.

Supplementary Figure 3d
colors were adjusted equally across the entire image
to improve the visualization of Alcian blue

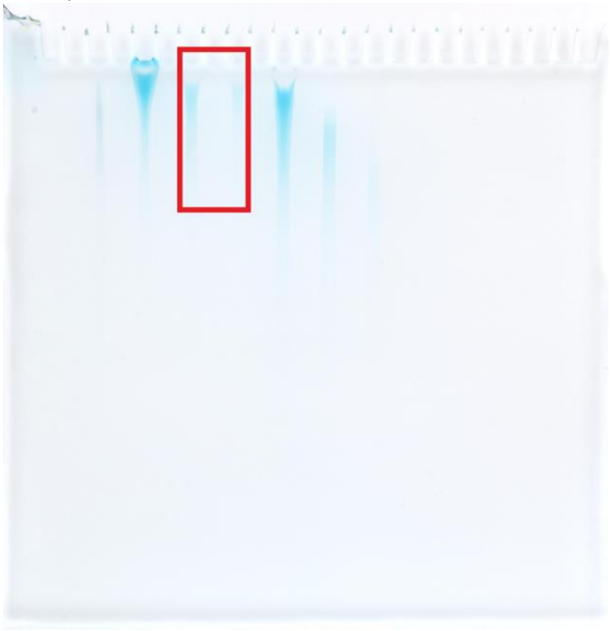

Supplementary Fig. 20 | Source Data for Supplementary Figure 3. Unprocessed gel.

Supplementary Figure 4g

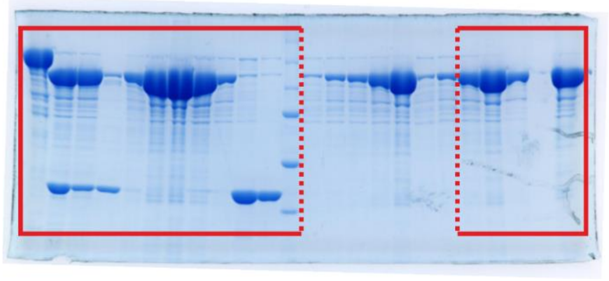

Supplementary Fig. 21 | Source Data for Supplementary Figure 4. Unprocessed gel.

Supplementary Figure 10a

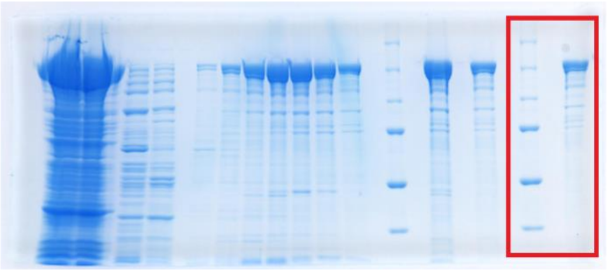

Supplementary Figure 10b
colors were adjusted equally across the entire image to improve the visualization of Alcian blue

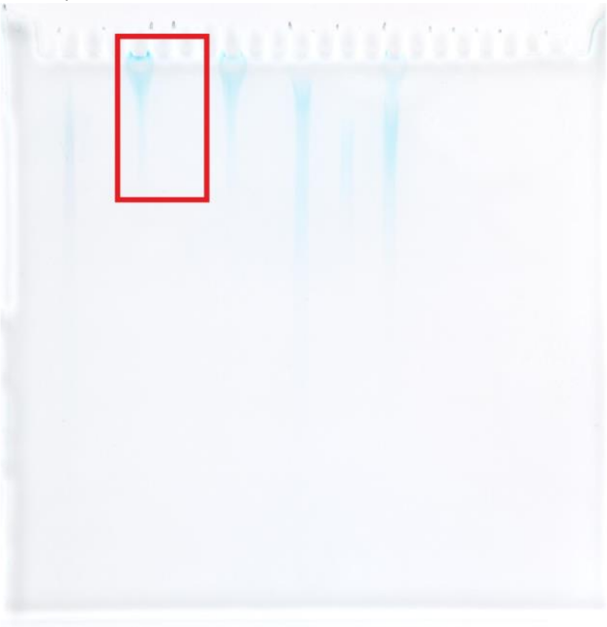

Supplementary Fig. 22 | Source Data for Supplementary Figure 10. Unprocessed gels.

SUPPLEMENTARY NOTES

Supplementary Note 1 | Development of a multi-enzyme cascade suitable for generating Hib polymer at mg-scale and production of oligomers.

Generating polymer by use of enzyme cascades can potentially omit biohazard and costs during glycoconjugate vaccine production and might even allow the generation of tailored antigens that can help to improve our understanding of these molecules[53–57]. In the reaction shown in Extended Data Fig. 2, commercially available PRPP was supplied and the synthesis of CDP-ribitol was started with ribose-5-phosphate (Rib-5P). Here, we wanted to investigate if the up-scaled synthesis of both substrates could start from inexpensive ribose (Rib) or Rib-5P. Two additional enzymes are required to achieve this goal: A ribokinase (RK) for the production of Rib-5P from Rib[58], and PrsA, the PRPP synthase[18] (Extended Data Fig. 7a). Both enzymes were cloned and purified from Hib lysates (Supplementary Fig. 2). Hib RK is likely able to utilize ATP, CTP, and UTP as phosphoryl donor, as indicated by the production of the corresponding nucleoside diphosphates (Extended Data Fig. 7b). In contrast, nucleoside monophosphate, the reaction product of the PrsA reaction (Extended Data Fig. 7a), was only produced in the presence of ATP, indicating that CTP and UTP are no suitable substrates for Hib PrsA (Extended Data Fig. 7c).

Polymer could be produced in a reaction, in which PRPP was synthesized *in situ* from Rib-5P using PrsA, demonstrating that PrsA is active in a one-pot reaction with RpiA, Bcs1, Bcs3, and their substrates (Extended Data Fig. 7g, lane 1). Additionally, CDP-ribitol could be produced from Rib in a reaction containing RK (with ATP as phosphoryl donor), RpiA, and Bcs1 (Extended Data Fig. 7e), proving RK to be active in the presence of RpiA, Bcs1, and their substrates. However, synthesizing polymer from Rib only yielded considerable amounts of product if UTP and not ATP was used as phosphoryl donor for RK, and if RK was heat-inactivated before adding PrsA/ATP required for the down-stream reaction (Extended Data Fig. 7g, compare lane 2 and 3 with lane 1). This is likely due to the fact that ADP produced from ATP by RK inhibits PrsA[18]. We could confirm an effect of ADP on Hib PrsA activity by monitoring the ATP consumption using HPLC-AEC: ATP could only be completely consumed in an overnight reaction in the absence of ADP (Extended Data Fig. 7d)

Since the use of UTP instead of ATP would - at milligram / laboratory-scale as shown herein - compensate the financial advantage of starting the reaction from ribose, we decided to only include PrsA in the cascade and start the reaction from Rib-5P. Conveniently, the concentration of both donor substrates (PRPP and CDP-ribitol) and thus the equal use of ribose-5-phosphate by both arms of the cascade, can be adjusted by controlling the supply of ATP (PrsA reaction) and CTP/NADPH (Bcs1 reaction) (Extended Data Fig. 7a). The reaction was upscaled to yield 50 mg of polymer. HPLC-AEC demonstrates that all donor substrates (ATP, CTP, CDP-ribitol, NADPH) were almost completely consumed (Extended Data Fig. 7f). The reaction was purified by preparative AEC using a MonoQ column. Due to the fact that Hib polymer is UV-inactive, polymer elution was monitored using the pre-column pressure (Extended Data Fig. 7h). Polymer-containing fractions were confirmed by Alcian blue / silver stained PAGE (Extended Data Fig. 7i), pooled, and dialyzed against water to remove sodium chloride. Hydrolysis conditions were determined at small-scale in 10 mM acetic acid (70°C) as previously described[59] and in 25 mM trifluoroacetic acid (TFA, 37°C) (Extended Data Fig. 7j). Based on these results, 50 mg of polymer were hydrolyzed in 25 mM TFA for

120 min and 37°C to obtain fragments of intermediate to small size. These fragments were again separated and purified by preparative AEC and fractions were combined into 12 pools, of which pools 2-12 contained detectable amounts of oligomers (Extended Data Fig. 7k). Despite eluting with increasing salt concentration from the MonoQ column, the fragments were not separated solely according to their molecular size in the PAGE. It is unclear if shifts towards lower molecular weights in fraction >5 might be due to the increasing salt concentration present in each fraction. To investigate if pools >5 contained polymers with different reducing or non-reducing ends, Pool 2 and 7 were characterized by 2D NMR and compared by 1D ^{1}H NMR, demonstrating that fragments in both pools carried Rib*f* at their non-reducing end and Rib*f* (both α and β) and Rib*p* (both α and β) at their reducing end. No other termini could be identified, indicating that the irregular migration behavior is not due to different termini and that both pools were comparatively homogeneous. Pool 3 was used for the biochemical characterization of Bcs3, pool 4 (avDP9-10) was used for co-crystallization.

SUPPLEMENTARY DISCUSSION

Supplementary Discussion | The elongation mechanism of group 2 capsule polymerases.

The product profile displayed by Bcs3 in reactions containing different donor to acceptor ratios (d/a ratio) is of high dispersity (Extended Data Fig. 8a,b), long chains are produced already at low d/a ratios (e.g. 10) while neglected acceptors are still visible. All of these observations are hallmarks of processive elongation[60]. In contrast, when single MBP-/His-tagged domains are used in *trans*, creating a situation that likely disrupts the concerted interplay of the enzymes and that should thus be inferior to the native optimized wildtype complex, chains are considerably shorter and product populations are less disperse (Extended Data Fig. 8c), suggesting a more distributive elongation and/or reduced reaction efficacy. The molecular basis for processivity of group 2 capsule polymerases has been investigated intensively: Glycosaminoglycan (GAGs) polymerases, like K4CP of *E. coli* K4, are generally considered distributive under *in vitro* conditions[61]. In agreement with that, the structure of K4CP demonstrates that the active sites of the two domains are on opposite sides of the enzyme and no evidence for a positively-charged binding site could be identified in the structure[62,63]. In contrast, all active sites of Bcs3 are facing towards the SH3b acceptor binding domain. Bacterial polysialyltransferases (PSTs) can exhibit some degree of processivity *in vitro*[64]. The structure of the *Mannheimia haemolytica* serotype A2 PST[65] (*Mh*PST) was solved in the presence of the negatively charged acceptor analogue fondaparinux and shows electropositive areas at the front of the N-terminal Rossmann domain. However, the structure could not support the experimental evidence obtained through *in vitro* studies with the *Neisseria meningitidis* serogroup B (*Nm*B) PST, which indicate increasing affinity towards the acceptor with increasing acceptor length, arguing for an extended polysialic acid binding site associated with residue K69[64]. In summary, structural evidence of binding modules or extended binding sites in group 2 capsule polymerases has so far been missing. Biochemical data supporting that such binding sites mediate processive elongation can be found for the capsule polymerases CsxA (*N. meningitidis* serogroup X) and Cps1B (*A. pleuropneumoniae* serotype 1). The elongation mechanism of CsxA could be switched to distributive by removing an extended C-terminal binding site of 98 residues[66], and Cps1B was rendered distributive by removing the C-terminal tetratricopeptide repeat domain (392 residues)[67]. However, no structural evidence for these binding sites exists so far. It is of note that the SH3b domain of Bcs3 could not be removed without loss of activity and is present even in the homologues of Bcs3 from Gram-positive sources (Supplementary Data 1), indicating that it contributes considerably to the enzyme's function. Nevertheless, in homologues of Bcs3 found in *E. coli* K18, *Staphylococcus epidermidis*, and *Clostridium botulinum*, the SH3b domain is absent. It is important to consider that an SH3b domain could be supplied by a separate polypeptide or substituted by another binding module with different fold but homologous function in the *in vivo* situation. Interestingly, the C-terminal binding site of the processive polymerase CsxA is not present in the distributive polymerase CsaB[66], even though both enzymes (i) are expressed in a group 2 context by *Neisseria meningitidis* (different serotypes), (ii) belong to the *stealth* protein family, and (iii) share considerably sequence identity[68].

SUPPLEMENTARY REFERENCES

1. Lemercinier, X. & Jones, C. An NMR Spectroscopic Identity Test for the Control of the Capsular Polysaccharide from Haemophilus influenzae Type b. *Biologicals* **28**, 175–183 (2000).
2. Markley, J. L. *et al.* Recommendations for the presentation of NMR structures of proteins and nucleic acids. IUPAC-IUBMB-IUPAB Inter-Union Task Group on the Standardization of Data Bases of Protein and Nucleic Acid Structures Determined by NMR Spectroscopy. *J Biomol NMR* **12**, 1–23 (1998).
3. Maurer & Kalbitzer. Indirect Referencing of 31P and 19F NMR Spectra. *J Magn Reson B* **113**, 177–8 (1996).
4. Garegg, P. J., Johansson, R., Lindh, I. & Samuelsson, B. Synthesis of p-aminophenyl beta-D-ribofuranoside 3-(D-ribit-5-yl phosphate). *Carbohydr Res* **150**, 285–9 (1986).
5. Ravenscroft, N. Size determination of bacterial capsular oligosaccharides used to prepare conjugate vaccines. *Vaccine* **17**, 2802–2816 (1999).
6. Napolitano, J. G. *et al.* On the configuration of five-membered rings: a spin-spin coupling constant approach. *Chemistry* **17**, 6338–47 (2011).
7. King-Morris, M. J. & Serianni, A. S. Carbon-13 NMR studies of [1-13C]aldoses: empirical rules correlating pyranose ring configuration and conformation with carbon-13 chemical shifts and carbon-13/carbon-13 spin couplings. *J Am Chem Soc* **109**, 3501–3508 (1987).
8. Lovering, A. L. *et al.* Structure of the bacterial teichoic acid polymerase TagF provides insights into membrane association and catalysis. *Nat Struct Mol Biol* **17**, 582–9 (2010).
9. Litschko, C. *et al.* A New Family of Capsule Polymerases Generates Teichoic Acid-Like Capsule Polymers in Gram-Negative Pathogens. *mBio* **9**, 16017 (2018).
10. Mirdita, M. *et al.* ColabFold: making protein folding accessible to all. *Nat Methods* **19**, 679–682 (2022).
11. Jumper, J. *et al.* Highly accurate protein structure prediction with AlphaFold. *Nature* **596**, 583–589 (2021).
12. Gautier, R., Douguet, D., Antonny, B. & Drin, G. HELIQUEST: a web server to screen sequences with specific -helical properties. *Bioinformatics* **24**, 2101–2102 (2008).
13. Laskowski, R. A. & Swindells, M. B. LigPlot+: Multiple ligand-protein interaction diagrams for drug discovery. *J Chem Inf Model* **51**, 2778–2786 (2011).
14. Héroux, A., White, E. L., Ross, L. J., Kuzin, A. P. & Borhani, D. W. Substrate deformation in a hypoxanthine-guanine phosphoribosyltransferase ternary complex: The structural basis for catalysis. *Structure* **8**, 1309–1318 (2000).
15. Silva, C. H. T. P., Silva, M., Iulek, J. & Thiemann, O. H. Structural complexes of human adenine phosphoribosyltransferase reveal novel features of the aprt catalytic mechanism. *J Biomol Struct Dyn* **25**, 589–597 (2008).
16. Grubmeyer, C., Hansen, M. R., Fedorov, A. A. & Almo, S. C. Structure of Salmonella typhimurium OMP synthase in a complete substrate complex. *Biochemistry* **51**, 4397–4405 (2012).
17. Schumacher, M. A. *et al.* The structural mechanism of GTP stabilized oligomerization and catalytic activation of the Toxoplasma gondii uracil phosphoribosyltransferase. *Proc Natl Acad Sci U S A* **99**, 78–83 (2002).
18. Hove-Jensen, B. *et al.* Phosphoribosyl Diphosphate (PRPP): Biosynthesis, Enzymology, Utilization, and Metabolic Significance. *Microbiol Mol Biol Rev* **81**, e00040-16 (2017).
19. González-Segura, L., Witte, J. F., McClard, R. W. & Hurley, T. D. Ternary complex formation and induced asymmetry in orotate phosphoribosyltransferase. *Biochemistry* **46**, 14075–14086 (2007).
20. Scapin, G., Grubmeyer, C. & Sacchettini, J. C. Crystal Structure of Orotate Phosphoribosyltransferase. *Biochemistry* **33**, 1287–1294 (1994).
21. Roca, M. *et al.* Elucidating the Catalytic Reaction Mechanism of Orotate Phosphoribosyltransferase by Means of X-ray Crystallography and Computational Simulations. *ACS Catal* **10**, 1871–1885 (2020).

22. Wang, W., Kim, R., Jancarik, J., Yokota, H. & Kim, S. H. Crystal structure of phosphoserine phosphatase from Methanococcus jannaschii, a hyperthermophile, at 1.8 Å resolution. *Structure* **9**, 65–71 (2001).
23. Seifried, A., Schultz, J. & Gohla, A. Human HAD phosphatases: structure, mechanism, and roles in health and disease. *FEBS Journal* **280**, 549–571 (2013).
24. Fonda, M. L. & Zhang, Y. na. Kinetic mechanism and divalent metal activation of human erythrocyte pyridoxal phosphatase. *Arch Biochem Biophys* **320**, 345–352 (1995).
25. Knobloch, G. *et al.* Synthesis of hydrolysis-resistant pyridoxal 5′-phosphate analogs and their biochemical and X-ray crystallographic characterization with the pyridoxal phosphatase chronophin. *Bioorg Med Chem* **23**, 2819–2827 (2015).
26. Burroughs, A. M., Allen, K. N., Dunaway-Mariano, D. & Aravind, L. Evolutionary Genomics of the HAD Superfamily: Understanding the Structural Adaptations and Catalytic Diversity in a Superfamily of Phosphoesterases and Allied Enzymes. *J Mol Biol* **361**, 1003–1034 (2006).
27. Kuznetsova, E. *et al.* Functional diversity of haloacid dehalogenase superfamily phosphatases from Saccharomyces cerevisiae: Biochemical, structural, and evolutionary insights. *Journal of Biological Chemistry* **290**, 18678–18698 (2015).
28. Schertzer, J. W., Bhavsar, A. P. & Brown, E. D. Two conserved histidine residues are critical to the function of the TagF-like family of enzymes. *Journal of Biological Chemistry* **280**, 36683–36690 (2005).
29. Sewell, E. W. C., Pereira, M. P. & Brown, E. D. The wall teichoic acid polymerase TagF is non-processive in Vitro and amenable to study using steady state kinetic analysis. *Journal of Biological Chemistry* **284**, 21132–21138 (2009).
30. Wang, W. *et al.* Structural Characterization of the Reaction Pathway in Phosphoserine Phosphatase: Crystallographic "snapshots" of Intermediate States. *J Mol Biol* **319**, 421–431 (2002).
31. Hou, Z., Zhang, H., Li, M. & Chang, W. Structure of 2-haloacid dehalogenase from Pseudomonas syringae pv. tomato DC3000. *Acta Crystallogr D Biol Crystallogr* **69**, 1108–1114 (2013).
32. Chen, S. C. *et al.* Mechanism and inhibition of human UDP-GlcNAc 2-epimerase, the key enzyme in sialic acid biosynthesis. *Sci Rep* **6**, (2016).
33. He, J. bin *et al.* Molecular and Structural Characterization of a Promiscuous C-Glycosyltransferase from Trollius chinensis. *Angewandte Chemie - International Edition* vol. 58 11513–11520 Preprint at https://doi.org/10.1002/anie.201905505 (2019).
34. Rocha, J. *et al.* Structural insights and membrane binding properties of MGD1, the major galactolipid synthase in plants. *Plant Journal* **85**, 622–633 (2016).
35. Lairson, L. L., Henrissat, B., Davies, G. J. & Withers, S. G. Glycosyltransferases: structures, functions, and mechanisms. *Annu Rev Biochem* **77**, 521–555 (2008).
36. Albesa-Jové, D. & Guerin, M. E. The conformational plasticity of glycosyltransferases. *Curr Opin Struct Biol* **40**, 23–32 (2016).
37. Albesa-Jové, D., Giganti, D., Jackson, M., Alzari, P. M. & Guerin, M. E. Structure-function relationships of membrane-associated GT-B glycosyltransferases. *Glycobiology* **24**, 108–124 (2014).
38. Hu, Y. *et al.* Crystal structure of the MurG:UDP-GlcNAc complex reveals common structural principles of a superfamily of glycosyltransferases. *Proc Natl Acad Sci U S A* **100**, 845–849 (2003).
39. Buschiazzo, A. *et al.* Crystal structure of glycogen synthase: homologous enzymes catalyze glycogen synthesis and degradation. *EMBO J* **23**, 3196–3205 (2004).
40. Giganti, D. *et al.* Conformational plasticity of the essential membrane-associated mannosyltransferase PimA from mycobacteria. *Journal of Biological Chemistry* **288**, 29797–29808 (2013).
41. Giganti, D. *et al.* Secondary structure reshuffling modulates glycosyltransferase function at the membrane. *Nat Chem Biol* **11**, 16–18 (2015).

42. Rodrigo-Unzueta, A. *et al.* Dissecting the Structural and Chemical Determinants of the 'Open-to-Closed' Motion in the Mannosyltransferase PimA from Mycobacteria. *Biochemistry* **59**, 2934–2945 (2020).
43. Guerin, M. E. *et al.* Substrate-induced conformational changes in the essential peripheral membrane-associated mannosyltransferase PimA from mycobacteria: implications for catalysis. *J Biol Chem* **284**, 21613–21625 (2009).
44. Liebau, J. *et al.* Unveiling the activation dynamics of a fold-switch bacterial glycosyltransferase by 19 F NMR. *J Biol Chem* **295**, 9868–9878 (2020).
45. Vetting, M. W., Frantom, P. A. & Blanchard, J. S. Structural and enzymatic analysis of MshA from Corynebacterium glutamicum: substrate-assisted catalysis. *J Biol Chem* **283**, 15834–15844 (2008).
46. Albesa-Jové, D., Cifuente, J. O., Trastoy, B. & Guerin, M. E. Quick-soaking of crystals reveals unprecedented insights into the catalytic mechanism of glycosyltransferases. *Methods Enzymol* **621**, 261–279 (2019).
47. Albesa-Jové, D., Sainz-Polo, M. Á., Marina, A. & Guerin, M. E. Structural Snapshots of α-1,3-Galactosyltransferase with Native Substrates: Insight into the Catalytic Mechanism of Retaining Glycosyltransferases. *Angew Chem Int Ed Engl* **56**, 14853–14857 (2017).
48. Gonzalez-Delgado, L. S. *et al.* Two-site recognition of Staphylococcus aureus peptidoglycan by lysostaphin SH3b. *Nat Chem Biol* **16**, 24–30 (2020).
49. Lu, J. Z., Fujiwara, T., Komatsuzawa, H., Sugai, M. & Sakon, J. Cell wall-targeting domain of glycylglycine endopeptidase distinguishes among peptidoglycan cross-bridges. *Journal of Biological Chemistry* **281**, 549–558 (2006).
50. Whisstock, J. C., Lesk, A. M., Whisstock, J. C. & Lesk, A. M. SH3 domains in prokaryotes. *Trends Biochem Sci* **24**, 132–133 (1999).
51. Marchler-Bauer, A. *et al.* CDD/SPARCLE: Functional classification of proteins via subfamily domain architectures. *Nucleic Acids Res* **45**, D200–D203 (2017).
52. Kelly, S. D. *et al.* The biosynthetic origin of ribofuranose in bacterial polysaccharides. *Nat Chem Biol* 413–454 (2022) doi:10.1038/s41589-022-01006-6.
53. Adamo, R. Advancing Homogeneous Antimicrobial Glycoconjugate Vaccines. *Acc Chem Res* **50**, 1270–1279 (2017).
54. Micoli, F. *et al.* Glycoconjugate vaccines: current approaches towards faster vaccine design. *Expert Rev Vaccines* **18**, 881–895 (2019).
55. Oldrini, D. *et al.* Combined Chemical Synthesis and Tailored Enzymatic Elongation Provide Fully Synthetic and Conjugation-Ready Neisseria meningitidis Serogroup X Vaccine Antigens. *ACS Chem Biol* **13**, 984–994 (2018).
56. Fiebig, T. *et al.* An efficient cell free enzyme-based total synthesis of a meningococcal vaccine candidate. *NPJ Vaccines* **1**, 16017 (2016).
57. del Bino, L. *et al.* Synthetic Glycans to Improve Current Glycoconjugate Vaccines and Fight Antimicrobial Resistance. *Chemical Reviews* Preprint at https://doi.org/10.1021/acs.chemrev.2c00021 (2022).
58. Chuvikovsky, D. V. *et al.* Ribokinase from E. coli: Expression, purification, and substrate specificity. *Bioorg Med Chem* **14**, 6327–6332 (2006).
59. Costantino, P. *et al.* Size fractionation of bacterial capsular polysaccharides for their use in conjugate vaccines. *Vaccine* **17**, 1251–1263 (1999).
60. Yakovlieva, L. & Walvoort, M. T. C. Processivity in Bacterial Glycosyltransferases. *ACS Chem Biol* **15**, 3–16 (2020).
61. Jing, W. & DeAngelis, P. L. Synchronized chemoenzymatic synthesis of monodisperse hyaluronan polymers. *J Biol Chem* **279**, 42345–42349 (2004).
62. Osawa, T. *et al.* Crystal structure of chondroitin polymerase from Escherichia coli K4. *Biochem Biophys Res Commun* **378**, 10–14 (2009).

63. Willis, L. M. & Whitfield, C. Structure, biosynthesis, and function of bacterial capsular polysaccharides synthesized by ABC transporter-dependent pathways. *Carbohydr Res* **378**, 35–44 (2013).
64. Keys, T. G. *et al.* Engineering the product profile of a polysialyltransferase. *Nat Chem Biol* **10**, 437–442 (2014).
65. Lizak, C. *et al.* X-ray crystallographic structure of a bacterial polysialyltransferase provides insight into the biosynthesis of capsular polysialic acid. *Sci Rep* **7**, 5842 (2017).
66. Fiebig, T. *et al.* Efficient solid-phase synthesis of meningococcal capsular oligosaccharides enables simple and fast chemoenzymatic vaccine production. *J Biol Chem* **293**, 953–962 (2018).
67. Budde, I. *et al.* An enzyme-based protocol for cell-free synthesis of nature-identical capsular oligosaccharides from Actinobacillus pleuropneumoniae serotype 1. *J Biol Chem* **295**, 5771–5784 (2020).
68. Sperisen, P., Schmid, C. D., Bucher, P. & Zilian, O. Stealth proteins: in silico identification of a novel protein family rendering bacterial pathogens invisible to host immune defense. *PLoS Comput Biol* **1**, 0492–0499 (2005).
69. Boratyn, G. M. *et al.* BLAST: a more efficient report with usability improvements. *Nucleic Acids Res* **41**, W29-33 (2013).
70. Papadopoulos, J. S. & Agarwala, R. COBALT: constraint-based alignment tool for multiple protein sequences. *Bioinformatics* **23**, 1073–1079 (2007).
71. Waterhouse, A. M., Procter, J. B., Martin, D. M. A., Clamp, M. & Barton, G. J. Jalview Version 2--a multiple sequence alignment editor and analysis workbench. *Bioinformatics* **25**, 1189–91 (2009).
72. Madeira, F. *et al.* Search and sequence analysis tools services from EMBL-EBI in 2022. *Nucleic Acids Res* (2022) doi:10.1093/nar/gkac240.
73. Swoboda, J. G., Campbell, J., Meredith, T. C. & Walker, S. Wall Teichoic Acid Function, Biosynthesis, and Inhibition. *ChemBioChem* **11**, 35–45 (2010).
74. Brown, S., Santa Maria, J. P. & Walker, S. Wall Teichoic Acids of Gram-Positive Bacteria. *Annu Rev Microbiol* **67**, 313–336 (2013).
75. Schertzer, J. W. & Brown, E. D. Purified, Recombinant TagF Protein from Bacillus subtilis 168 Catalyzes the Polymerization of Glycerol Phosphate onto a Membrane Acceptor in Vitro. *Journal of Biological Chemistry* **278**, 18002–18007 (2003).
76. Litschko, C. *et al.* Mix-and-Match System for the Enzymatic Synthesis of Enantiopure Glycerol-3-Phosphate-Containing Capsule Polymer Backbones from Actinobacillus pleuropneumoniae , Neisseria meningitidis , and Bibersteinia trehalosi. *mBio* **12**, e0089721 (2021).
