# Supplementary material for: A multi-enzyme machine polymerizes the Haemophilus influenzae type b capsule
Source: Nat Chem Biol. 2023 Jun 5;19(7):865–77. doi: 10.1038/s41589-023-01324-3 (PMC10299916; doi:10.1038/s41589-023-01324-3)
Supplement: Supplementary file 2 — Reporting Summary [file 41589_2023_1324_MOESM2_ESM.pdf]

nature portfolio

Corresponding author(s): Timm Fiebig
Marcelo E. Guerin

Last updated by author(s): Dec 8, 2022

Reporting Summary

Nature Portfolio wishes to improve the reproducibility of the work that we publish. This form provides structure for consistency and transparency in reporting. For further information on Nature Portfolio policies, see our Editorial Policies and the Editorial Policy Checklist.

Statistics

For all statistical analyses, confirm that the following items are present in the figure legend, table legend, main text, or Methods section.

n/a	Confirmed	
☒	☐	The exact sample size (n) for each experimental group/condition, given as a discrete number and unit of measurement
☒	☐	A statement on whether measurements were taken from distinct samples or whether the same sample was measured repeatedly
☒	☐	The statistical test(s) used AND whether they are one- or two-sided<br>*Only common tests should be described solely by name; describe more complex techniques in the Methods section.*
☒	☐	A description of all covariates tested
☒	☐	A description of any assumptions or corrections, such as tests of normality and adjustment for multiple comparisons
☒	☐	A full description of the statistical parameters including central tendency (e.g. means) or other basic estimates (e.g. regression coefficient) AND variation (e.g. standard deviation) or associated estimates of uncertainty (e.g. confidence intervals)
☒	☐	For null hypothesis testing, the test statistic (e.g. F, t, r) with confidence intervals, effect sizes, degrees of freedom and P value noted<br>*Give P values as exact values whenever suitable.*
☒	☐	For Bayesian analysis, information on the choice of priors and Markov chain Monte Carlo settings
☒	☐	For hierarchical and complex designs, identification of the appropriate level for tests and full reporting of outcomes
☒	☐	Estimates of effect sizes (e.g. Cohen's d, Pearson's r), indicating how they were calculated

Our web collection on statistics for biologists contains articles on many of the points above.

Software and code

Policy information about availability of computer code

Data collection

We did not generate any new software for our work. Data collection software includes:
- NMR: Data were collected with Topspin 3.6.1 (Bruker Biospin, Germany)
- HPLC: Data were collected with LCsolution version 1.25 SP4 (Shimadzu)
- Gels were scanned using Amersham Imager 680, Software: AI600_66830510 Version2.0.0

Data analysis

We did not generate any new software for our work. Data analysis software includes:
- NMR: Spectra were processed with Topspin 3.6.1 (Bruker Biospin, Germany) and analyzed with Sparky 3.115 and 3.111 (Goddard T.D., Kneller D.G. (2008) SPARKY 3. University of California, San Francisco, CA).
HPLC: Data analysis was performed with LCsolution version 1.25 SP4 (Shimadzu).
Nucleotide and protein sequences were identified with the BLAST suite of programs from the National Center for Biotechnology Information (NCBI).
phenix-1.18.2-3874
coot 0.8.9.2
UCSF Chimera 1.14
USCF ChimeraX 1.3
Phaser 2.5
Buccaneer 1.0
CCP4 1.18.2
Refmac5
MolProbity Online Version 4.4 available at http://molprobity.biochem.duke.edu/
Hollow 1.1

PISA, Web service available at https://www.ebi.ac.uk/pdbe/pisa/
MUSCLE version 3.8.31
Cobalt, a constraint-based multiple sequence alignment tool from the National Center for Biotechnology Information (NCBI), National Institutes of Health (NIH) available at https://www.ncbi.nlm.nih.gov/tools/cobalt/cobalt.cgi?CMD=Web
Clustal Omega, Online Version, available at https://www.ebi.ac.uk/Tools/msa/clustalo/
DALI server, a network service for comparing protein structures in 3D available at http://ekhidna2.biocenter.helsinki.fi/dali.
Alphafold, web service available at https://colab.research.google.com/github/sokrypton/ColabFold/blob/main/AlphaFold2.ipynb

For manuscripts utilizing custom algorithms or software that are central to the research but not yet described in published literature, software must be made available to editors and reviewers. We strongly encourage code deposition in a community repository (e.g. GitHub). See the Nature Portfolio guidelines for submitting code & software for further information.

Data

Policy information about availability of data

All manuscripts must include a data availability statement. This statement should provide the following information, where applicable:

- Accession codes, unique identifiers, or web links for publicly available datasets
- A description of any restrictions on data availability
- For clinical datasets or third party data, please ensure that the statement adheres to our policy

The atomic coordinates and structure factors have been deposited in the Protein Data Bank, accession codes 8A0C for the Bcs3-CMP complex and 8A0M for the Bcs3-DP2 complex. Data collection and refinement statistics are presented in Supplementary Table 3. PDB IDs used in the analysis of this work include 1ZN7, 1LH0, 1FSG, 1JLS, 1L7N, 5AES, 3VAY, 1L7P, 3L7K, 4ZHT, 6JDT, 4X1T, 5LEO, 6RJE, 1R77, 7BNH and 7SHG. Accession codes for sequences used in this study are available in Supplementary Table 4 and Extended Data Figure 1. NMR chemical shifts are presented in Supplementary Tables 1 and 2. Source Data is provided in separate files.

Human research participants

Policy information about studies involving human research participants and Sex and Gender in Research.

Reporting on sex and gender	This is not relevant for this study.
Population characteristics	This is not relevant for this study.
Recruitment	This is not relevant for this study.
Ethics oversight	This is not relevant for this study.

Note that full information on the approval of the study protocol must also be provided in the manuscript.

Field-specific reporting

Please select the one below that is the best fit for your research. If you are not sure, read the appropriate sections before making your selection.

☒ Life sciences ☐ Behavioural & social sciences ☐ Ecological, evolutionary & environmental sciences

For a reference copy of the document with all sections, see nature.com/documents/nr-reporting-summary-flat.pdf

Life sciences study design

All studies must disclose on these points even when the disclosure is negative.

Sample size	Alignments were performed once with each algorithm and separate alogrithms, e.g. MUSCLE, Cobalt and Clustal omega, yielded highly similar results regarding the critical amino acids targeted in this study. Phyre2 homology modeling was performed in 2015, 2018, 2019, 2020 and 2021 to reevaluate the results and include potentially new entries to the PDB. All submissions yielded highly similar results. Sample sizes for enzymatic reactions were not predetermined on statistical methods, but were chosen according to common practices in enzyme research. Biochemical experiments producing qualitative results e.g. by visualization of polymer on a gel by Alcian blue / sivler staining, were performed at least three times with similar results. Polymer was synthesized four times at various scales with similar results. Constructs were purified once, the crystallization construct was purified twice. Test hydrolysis and preparative hydrolysis of polymer was performed once. To analyze the elongation mechanism of polymerase constructs, at least five reactions were performed at different donor to acceptor ratios with highly consistent results. These sample sizes are standard for investigations of this kind and were chosen as sufficient to represent any variance present in the samples but also to be within the technically practical limits for performing the experiment. There is no quantitative data that would require additional data points for statistical analysis. A detailed description on "statistics and reproducibility" is provided in the methods section.
Data exclusions	No data were excluded from the analyses.
Replication	see "samples size"

Randomization	Randomization was not necessary as no allocation of samples into experimental groups was required. In our experimental set up, defined enzyme variants were compared under well controlled conditions. Accordingly, the assays performed in this study did not depend on statistical analyses of an unknown relationship, but required a rational approach for activity comparison.
Blinding	Blinding was not relevant because results did not require subjective judgment or interpretation.

Reporting for specific materials, systems and methods

We require information from authors about some types of materials, experimental systems and methods used in many studies. Here, indicate whether each material, system or method listed is relevant to your study. If you are not sure if a list item applies to your research, read the appropriate section before selecting a response.

Materials & experimental systems

n/a	Involved in the study	
☐	☒	Antibodies
☒	☐	Eukaryotic cell lines
☒	☐	Palaeontology and archaeology
☒	☐	Animals and other organisms
☒	☐	Clinical data
☒	☐	Dual use research of concern

Methods

n/a	Involved in the study	
☒	☐	ChIP-seq
☒	☐	Flow cytometry
☒	☐	MRI-based neuroimaging

Antibodies

Antibodies used	rabbit anti-Hib agglutinating serum (Remel™/Thermo Fisher Scientific; in 1:1,000 or 1:2,000 dilution)
Validation	The agglutinating serum was used according to the manufacturer's guidlines. Specificity to Hib polymer was investigated by performing appropriate controls. NMR characterization unambiguously confirmed the identity of the polymer.
