# Supplementary material for: A multi-enzyme machine polymerizes the Haemophilus influenzae type b capsule
Source: Nat Chem Biol. 2023 Jun 5;19(7):865–77. doi: 10.1038/s41589-023-01324-3 (PMC10299916; doi:10.1038/s41589-023-01324-3)
Supplement: Supplementary file 3 — Sequence alignment of Bcs3 homologs—A BLAST69 search was performed using the amino acid sequence of full-length Bcs3 as query and hits with high sequence coverage were manually chosen, aligned using Cobalt70 and annotated using Jalview71. Homologs from Gram-negative and Gram-positive pathogens are highlighted in dark gray (top half of the alignment) or light gray (bottom half), respectively. Database references are provided. The secondary structural elements extracted from a selected protomer of Bcs3-CMP are shown above the alignment. Amino acid residues with at least 70 % of identity are highlighted in black. A sequence logo plot is depicted below the alignment to provide a detailed overview of the general consensus at every position. Amino acids H96, H233, D386, D680 and E818, which were targeted in this study by site-directed mutagenesis, are shown in red. The consensus of the DUF5776 according to the conserved domain database51 for the SH3b domain is shown in blue, highlighting that this domain is present in the majority of identified homologs. Interestingly, the C-terminal alpha-helical bundle (α56-α59) is only present in homologs having a Gram-negative background. The C-terminus of the E. coli sequence was manually aligned to allow a gap between α53 and α56. [file 41589_2023_1324_MOESM3_ESM.pdf]

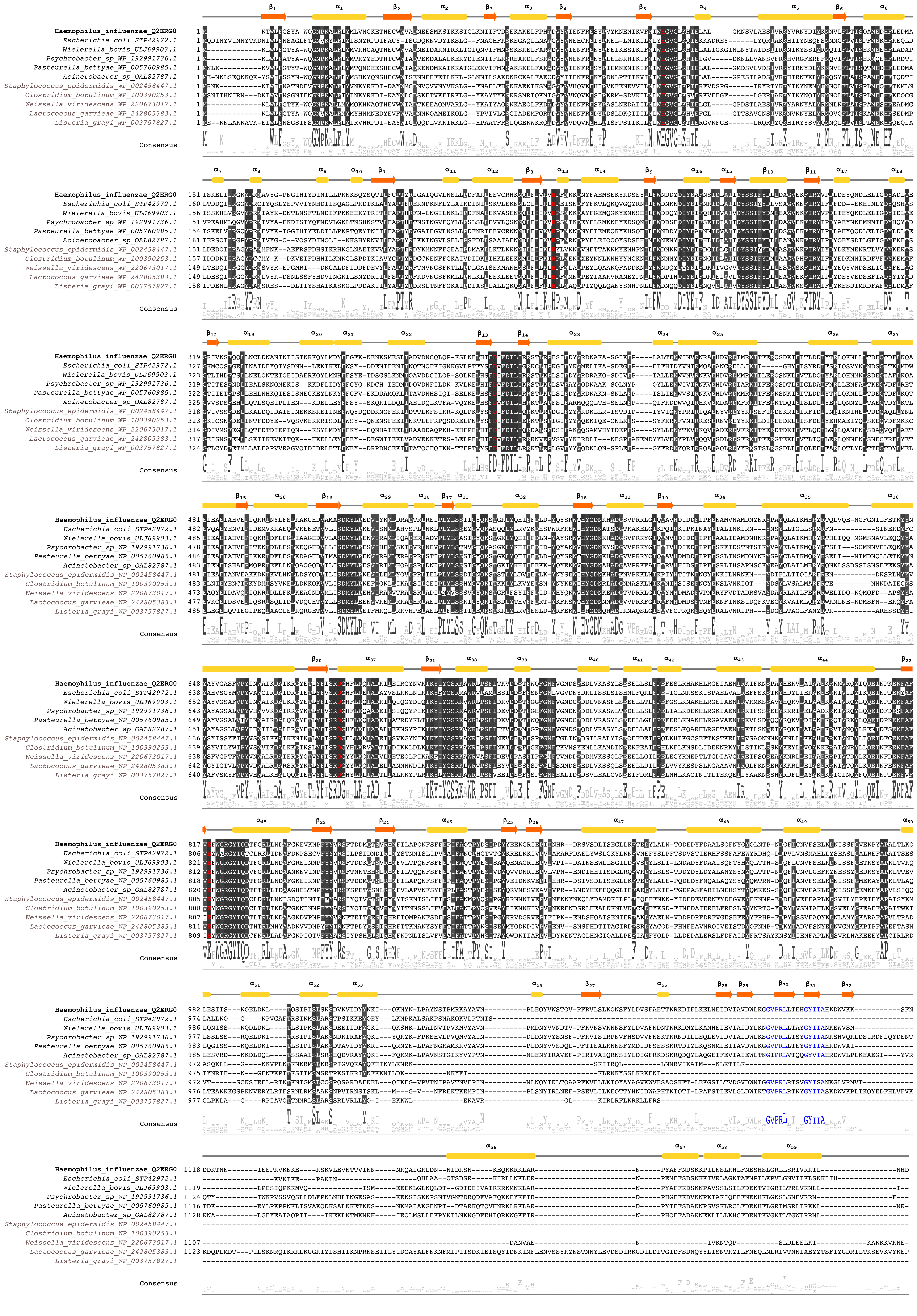

Haemophilus_influenzae_Q2ERG0
Escherichia_coli_STP42972.1
Wielerella_bovis_ULJ69903.1
Psychrobacter_sp_WP_192991736.1
Pasteurella_bettyae_WP_005760985.1
Acinetobacter_sp_OAL82787.1
Staphylococcus_epidermidis_WP_002458447.1
Clostridium_botulinum_WP_100390253.1
Weissella_viridescens_WP_220673017.1
Lactococcus_garvieae_WP_242805383.1
Listeria_grayi_WP_003757827.1
Consensus
