# Supplementary material for: A multi-enzyme machine polymerizes the Haemophilus influenzae type b capsule
Source: Nat Chem Biol. 2023 Jun 5;19(7):865–77. doi: 10.1038/s41589-023-01324-3 (PMC10299916; doi:10.1038/s41589-023-01324-3)
Supplement: Supplementary file 5 — Sequence alignment of Bcs3 with homologs from E. coli—Bcs3 was aligned using MUSCLE72 with the putative CroT-K18 (STI11141.1) and CrpP-CriT-K18 (STI11142.1) from E. coli K18 (see Extended Data Fig. 1), and with a homolog (STP42972.1) identified by BLAST (see Supplementary Data 1) in a non-serotyped E. coli strain, which expresses all three domains as single polypeptide and has high sequence identity with CroT-K18 and CrpP-CriT-K18. The secondary structural elements extracted from a selected protomer from Bcs3-CMP are shown above the alignment. The color code introduced for CroT, CrpP, CriT and SH3b in Figs. 1 and 3 was used to color the Bcs3 sequence. Amino acids targeted to separate the Bcs3 enzymes are highlighted in gray. [file 41589_2023_1324_MOESM5_ESM.pdf]

```
β1 α1 β2 α2 β3 α3 β4 β5 α4 α5
Haemophilus_influenzae_Q2ERG0     1 -------------MKTWLFGSY-AWQGNPKALFLYMLVNCKETHECWWVADNEESMKSIKKSTGLKNITFTDSEKAKELFPHADVYVTENFRESYPVYMNENIKVFNTWHGVGLKHIELALGMNSVLAESIVRKYVRN
Escherichia_coli_STP42972.1       1 MQDINYVINNYTKDNIWLFNSAGLFTGNVKYLFIYISNYRPDIFACYISGDQKNV--DYIKSLGFNACHFKS-KEGAQLLNAAGVYVNEHCKEIYPKELL-NTKILNLYHGVGLKQIEKKADREY-LYQRIAKKHITY
Escherichia_coli_STI11141.1       1 MQDINYVINNYTKDNIWLFNSAGLFTGNVKYLFIYISNYRPDIFACYISGDQKNV--DYIKSLGFNACHFKS-KEGAQLLNAAGVYVNEHCKEIYPKELL-NTKILNLYHGVGLKQIEKKADREY-LYQRIAKKHITY
Escherichia_coli_STI11142.1         ------------------------------------------------------------------------------------------------------------------------------------------

β6 α6 α7 α8 α9 α10 β7 α11 α12 β8 α13 α14 β9
Haemophilus_influenzae_Q2ERG0   125 YDIYKNNVLFLTTSQAMEDHFLEDMAISKELIIRGKYPRNAVYGPN-GIHTYDINTLLPKNKSQYSQTILFCPTYRIGAIQGVLNSLLPDFAKLEEVCRHKNQLFIVKVHPFMKKDNYFAEMSEKYKDSEYILFWNDD
Escherichia_coli_STP42972.1     134 NEQFSNNMCFLVTSPFMEKHFKEQLDLTDDQIIRGGYPRCIYQSLYEPVVTYNHDIISQAGLPKDTKLALYAPTFREKNPKNFLYLAIKDINILSKTLEKNNLCLIIKLHPEISNDFYFKTLQKQVGQYRNIIFWDNK
Escherichia_coli_STI11141.1     134 NEQFSNNMCFLVTSPFMEKHFKEQLDLTDDQIIRGGYPRCIYQSLYEPVVTYNHDIISQAGLPKDTKLALYAPTFREKNPKNFLYLAIKDINILSKTLEKNNLCLIIKLHPEISNDFYFKTLQKQVGQYRNIIFWDNK
Escherichia_coli_STI11142.1         ------------------------------------------------------------------------------------------------------------------------------------------

α15 β10 α16 β11 α17 α18 β12 α19 α20 α21 α22 β13 β14
Haemophilus_influenzae_Q2ERG0   262 YDIYEAFNSIDLAIIDYSSIFYDLLDAGVEKFIRYVPDLDEYQNDLELIGDYADLTEGRIVKSFQQLLNCLDNANIKIISTKRKQYLMDYFFGFKKENKSMESLIADVDNCQLQP-KSLKELHTFDIFDTLIRRSTLR
Escherichia_coli_STP42972.1     272 NDIYEIFNKIDLAIIDYSSIYYDLVSAGVKNFIRYIFDFDDEKH--IMLYDYQSHTSGKMCQSFGELINAIDEYQTY--SDNNLEKIKELFWSYDDEN-TFENIINQTNQFKIGNKGVLPTFYSFDVFDTLIGRKCLI
Escherichia_coli_STI11141.1     272 NDIYEIFNKIDLAIIDYSSIYYDLVSAGVKKFIRYIFDFDDEKH--IMLYDYQSHTSGKMCQSFGELINAIDEYQTY--SDNNLEK-------------------------------------------------------
Escherichia_coli_STI11142.1       1 -------------------------------------------------------------------------------------------------------------------------MPTFYSFDVFDTLIGRKCLI

α23 α24 α25 α26 α27 β15 α28 β16 α29 α30
Haemophilus_influenzae_Q2ERG0   399 PFSIFDYVRDKAKASGIKFPLALTENWINVRNRAEHDVRDIMRKTTFERQSDKIEITLDDIYTRLQKNLLLTDEQTDFLKQAEIEAEIAHVEPIQKRINYLFSLKAKGHDVAMASDMYLPEDVIYKMLDRADTRLREI
Escherichia_coli_STP42972.1     405 PKGIFYKVMELIKASDEGFPEIFHTDYVNIRMQAEANCRELLIKT-----IGHYEISYRDIFSRIQSVYNLNDSQITKLMDWEVQAEYENVIPIDEMVKKAEQLVKENETVVLISDMYLPKEVISNMLAHVSPLLNKL
Escherichia_coli_STI11141.1         ------------------------------------------------------------------------------------------------------------------------------------------
Escherichia_coli_STI11142.1      21 PKGIFYKVMELIKASDEGFPEIFHTDYVNIRMQAEANCRELLIKT-----IGHYEISYRDIFSRIQSVYNLNDSQITKLMDWEVQAEYENVIPIDEMVKKAEQLVKENETVVLISDMYLPKEVISNMLAHVSPLLNKL

β17 α31 α32 β18 α33 β19 α34 α35 α36
Haemophilus_influenzae_Q2ERG0   537 PLYLSSTIGYQKSTGKLYQHIFFD-LDYQYSRWTHYGDNKHADGSVPRRLGIQTAVHDIDDFIPFENAMVNAMDNYNRYPAYQLATKMHRYRTQLVQENGFGNTLFETKYYNYAYVGASFVPYINWAIKDAIKRGYET
Escherichia_coli_STP42972.1     538 PLYLSSEYGVQKASQQLYLRVYKDHSPWRFKEWIHYGDNATSDGTKAKELGIKPQIHKIPKINAYETALINKIRNYNSYLLTGLLSRMR------------FNSINEKDYFCYAHVSGYMVPYVAWCIRDAIDRGIET
Escherichia_coli_STI11141.1         ------------------------------------------------------------------------------------------------------------------------------------------
Escherichia_coli_STI11142.1     154 PLYLSSEYGVQKASQQLYLRVYKDHSPWRFKEWIHYGDNATSDGTKAKELGIKPQIHKIPKINAYETALINKIRNYNSYLLTGLLSRMR------------FNSINEKDYFCYAHVSGYMVPYVAWCIRDAIDRGIET

β20 α37 β21 α38 α39 α40 α41 α42 α43 α44
Haemophilus_influenzae_Q2ERG0   674 IYFISRDGHFLKQIADKIIEIRGYNVKTKYIYGSRKAWRLPSFITKVDDETFWQFGNFVGMDSFEDLVKASYLSESELLSLFPEFESLRHAKHLRGEIAENIRKIFKNSPAYHEKVLAIAAEKRKMVRQYIQQEINPK
Escherichia_coli_STP42972.1     664 LYFVSRDGHFLKEIADAYVSLKKLNIKTKYFYGSRRAWRVPAMIESIDDEFFSNFGNLVGVDNYDKLISSLSISHNLFQKLFPETGLNKSSK-ISPAELVALREFFSKSTKYHDYLIEKAKEERKIVIDYIKQEINPD
Escherichia_coli_STI11141.1         ------------------------------------------------------------------------------------------------------------------------------------------
Escherichia_coli_STI11142.1     280 LYFVSRDGHFLKEIADAYISLKKLNIKTKYFYGSRRAWRVPAMIESIDDEFFSNFGNLVGVDNYDKLISSLSISHNLFQKLFPETGLNKSSK-ISPAELVALREFFSKSTKYHDYLIEKAKEERKIVIDYIKQEINPD

β22 α45 β23 β24 α46 β25 β26 α47 α48
Haemophilus_influenzae_Q2ERG0   812 EKFAFVEFWGRGYTQDTFGRLLNDAFGKEVKNPFYYVRSFTDDMGTSVRHNFILAPQNFSFFEPIFAQTPYDSIPDYYEEKGRIEPIINHRDRSVSDLISEG--LLKFTEDYLALN-TQDEDYFDAALSQFNYQYQLN
Escherichia_coli_STP42972.1     801 EKYAFVEYWARGYTQTCLRKLIDNAFDKPSECVFYYYRSILPSIDNDIRINYSTNNISLIPVEAIFANCPHGTVTGYKRELKKIVPVIARARFDAELYWSLGKYLKNFVNEFYSLPFTSDVSTIERHFSSFAFEWYRD
Escherichia_coli_STI11141.1         ------------------------------------------------------------------------------------------------------------------------------------------
Escherichia_coli_STI11142.1     417 EKYAFVEYWARGYTQTCLRKLIDNAFDKPSECVFYYYRSILPSIDNDIRINYSTNNISLIPVEAIFANCPHGTVTGYKRELKKIVPVIARARFDAELYWSLGKYLKNFVNEFYSLPFTSDVSTIERHFSSFAFEWYRD

α49 α50 α51 α52 α53 α54 β27 α55 β28
Haemophilus_influenzae_Q2ERG0   947 TPNDQFICNVFSELKDNISSFGVEKPYAPALTLKQLESITSKQELDKLTQSIPISLSKSDVKVIDYYNKIQKNYNLPAYNSTPMRKAYAVNPLEQYVWSTQVPFRVLSLKQNSFYLDVSFAETTKRKDIFLKELNEID
Escherichia_coli_STP42972.1     939 HQDDPVLVNSMAHLLYSEASLGALREFAPAITSESLALLKQGKPVGAFTRSIKMSLARSTPSIKKEYQEYLKNPKA--LSAKPSNAKQKVLPTNTS-----------------------------------------
Escherichia_coli_STI11141.1         ------------------------------------------------------------------------------------------------------------------------------------------
Escherichia_coli_STI11142.1     555 HQDDPVLVNSMAHLLYSEASLGALREFAPAITSESLALLKQGKPVGAFTRSIKMSLARSTPSIKKEYQEYLKNPKA--LSAKPSNAKQKVLPTNTS-----------------------------------------

β29 β30 β31 β32 α56 α57 α58 α59
Haemophilus_influenzae_Q2ERG0  1085 VIAVDWLKGGVPRLLTEHGYITAHKDWVKKSFNDDKTNNIEEPKVKNKEKSKVLEVNTTVTNNNKQAIGKLDNNIDKSNKEQKKRKLARNPYAFFNDSKKPILNSLKHL-FNE--SHSLGRLLSRIVRKTLNHD
Escherichia_coli_STP42972.1    1033 ------------------------------------------KVKIKEPAKIN--------------QHLAAQTSDSRKIRLLNKLERNPEMFFSDSNKKIVRLAGKTAFNPILKPVLGNVIIKLSKKIIH--
Escherichia_coli_STI11141.1         -----------------------------------------------------------------------------------------------------------------------------------
Escherichia_coli_STI11142.1     649 ------------------------------------------KVKIKEPAKIN--------------QHLAAQSSDSRKIRLLNKLERNPEMFFSDSNKKIVRLAGKTAFNPILKPVLGNVIIKLSKKIIH--
```
