# Supplementary material for: A multi-enzyme machine polymerizes the Haemophilus influenzae type b capsule
Source: Nat Chem Biol. 2023 Jun 5;19(7):865–77. doi: 10.1038/s41589-023-01324-3 (PMC10299916; doi:10.1038/s41589-023-01324-3)

Figure 1f
colors were adjusted equally across the entire image to improve the visualization of Alcian blue

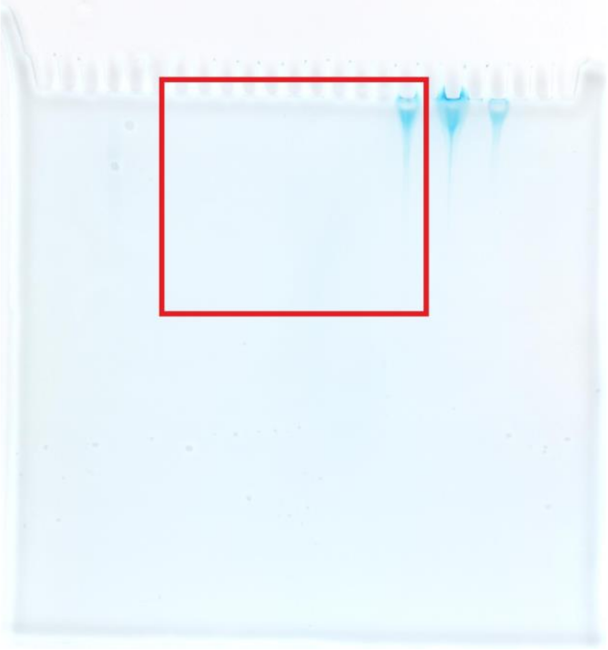

Figure 1g
rotated and flipped in the main figure

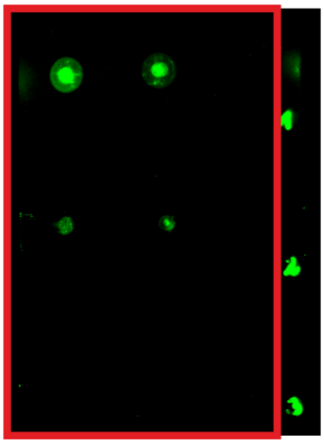

Supplement: Source Data Fig. 1 — Unprocessed gel and blot. [file 41589_2023_1324_MOESM6_ESM.pdf]
