# Supplementary material for: A multi-enzyme machine polymerizes the Haemophilus influenzae type b capsule
Source: Nat Chem Biol. 2023 Jun 5;19(7):865–77. doi: 10.1038/s41589-023-01324-3 (PMC10299916; doi:10.1038/s41589-023-01324-3)

Figure 3c
colors were adjusted equally across the entire image to improve the visualization of Alcian blue

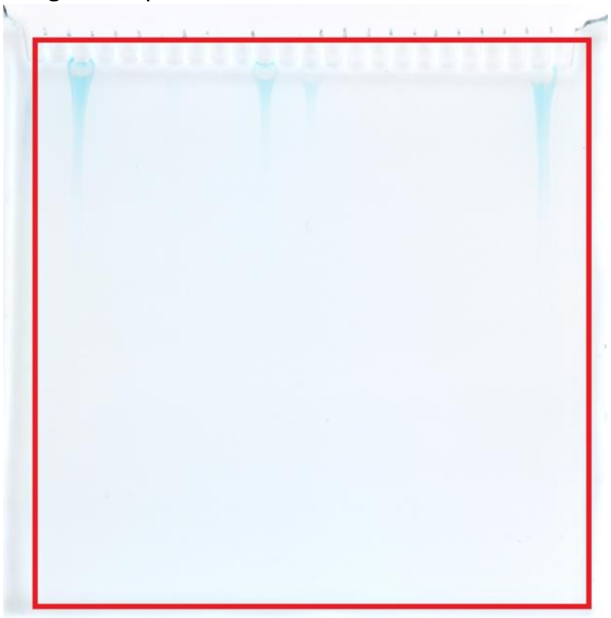

Figure 3d
colors were adjusted equally across the entire image to improve the visualization of Alcian blue

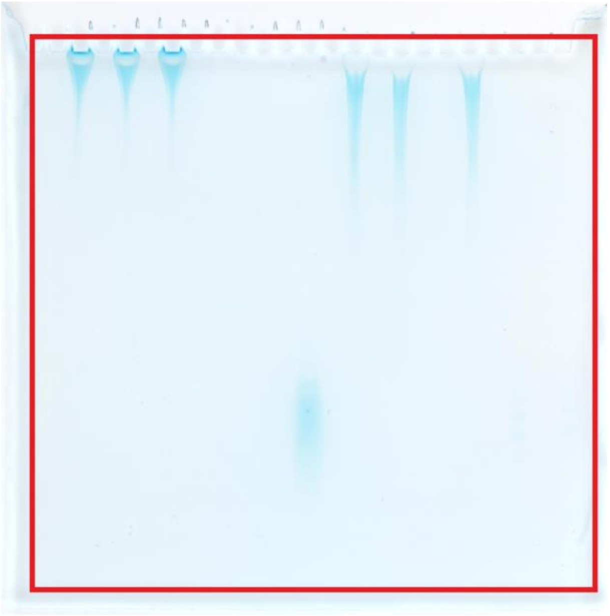

Figure 3e
colors were adjusted equally across the entire image to improve the visualization of Alcian blue

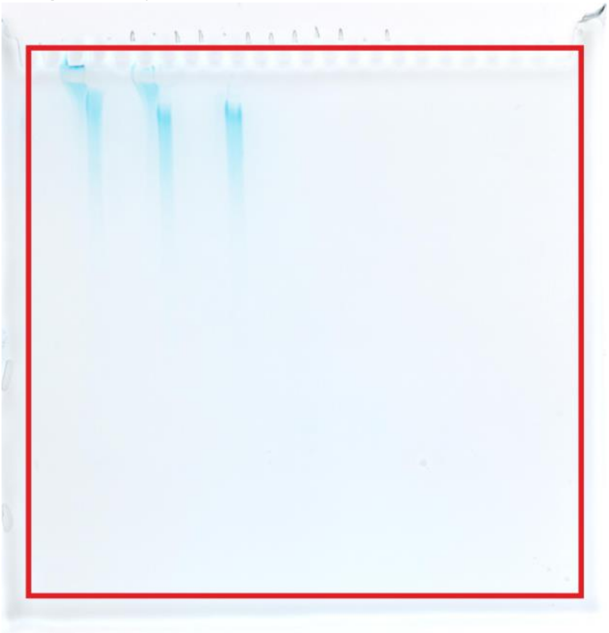

Figure 3f
colors were adjusted equally across the entire image to improve the visualization of Alcian blue

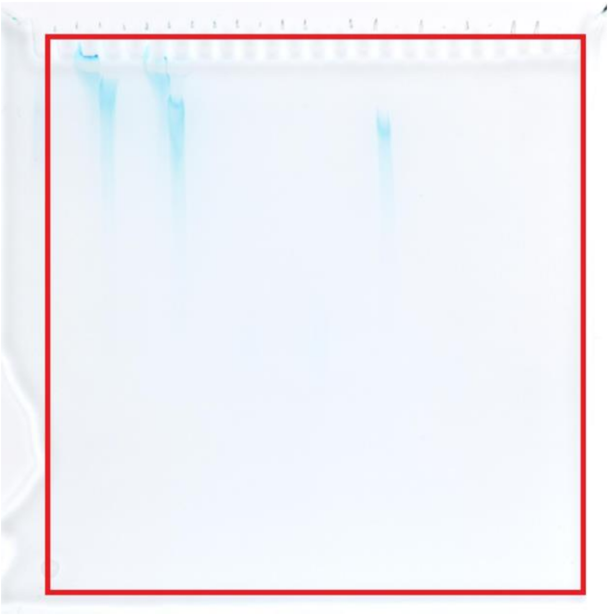

Supplement: Source Data Fig. 3 — Unprocessed gels. [file 41589_2023_1324_MOESM7_ESM.pdf]
