# Supplementary material for: A multi-enzyme machine polymerizes the Haemophilus influenzae type b capsule
Source: Nat Chem Biol. 2023 Jun 5;19(7):865–77. doi: 10.1038/s41589-023-01324-3 (PMC10299916; doi:10.1038/s41589-023-01324-3)

Extended Data Figure 2b

colors were adjusted equally across the entire image to improve the visualization of Alcian blue

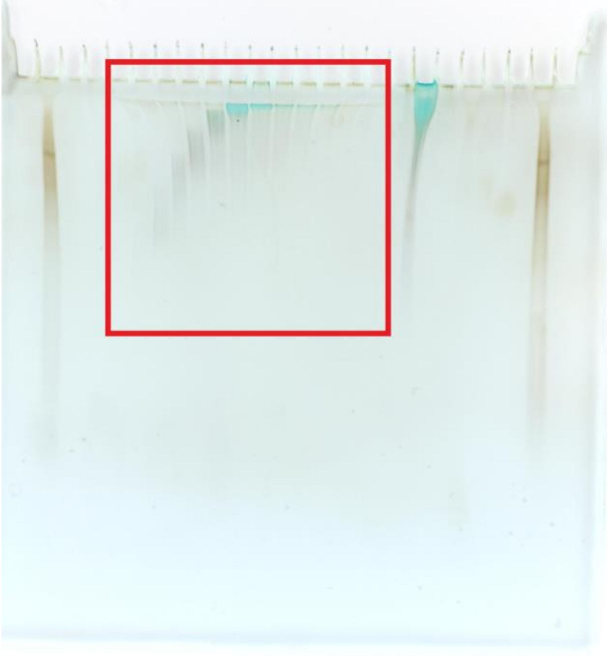

Supplement: Source Data Extended Data Fig. 2 — Unprocessed gel. [file 41589_2023_1324_MOESM8_ESM.pdf]
