# Supplementary material for: A multi-enzyme machine polymerizes the Haemophilus influenzae type b capsule
Source: Nat Chem Biol. 2023 Jun 5;19(7):865–77. doi: 10.1038/s41589-023-01324-3 (PMC10299916; doi:10.1038/s41589-023-01324-3)

Extended Data Figure 7g
flipped in the main figure
colors were adjusted equally across the entire image to improve the visualization of Alcian blue

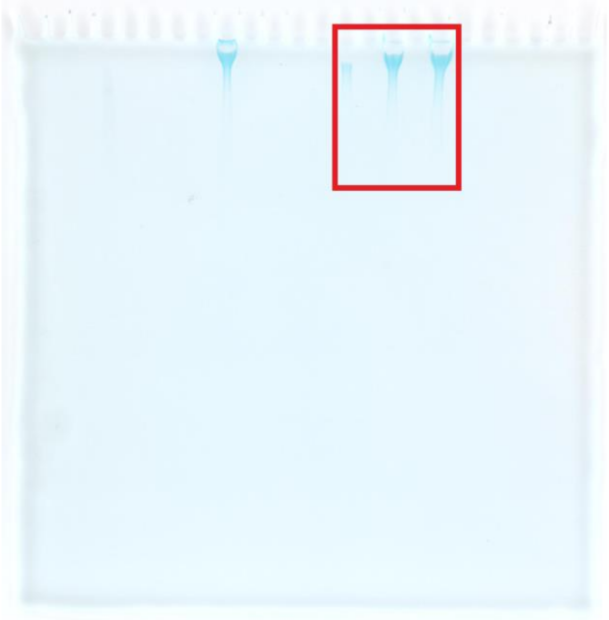

Extended Data Figure 7i
colors were adjusted equally across the entire image to improve the visualization of Alcian blue

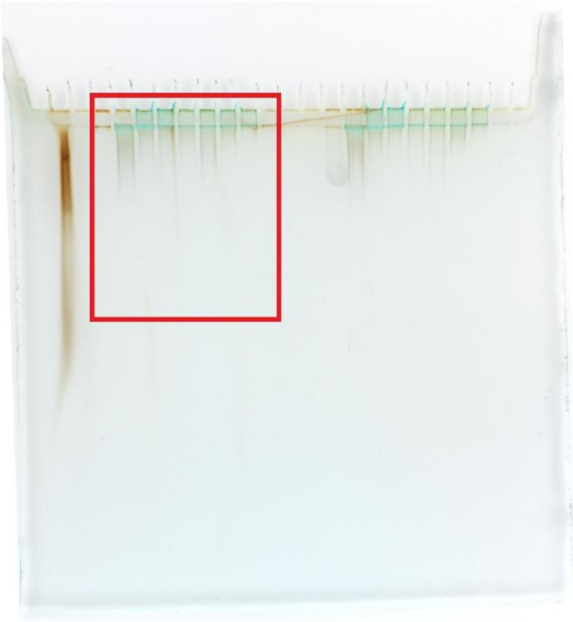

Extended Data Figure 7j
colors were adjusted equally across the entire image to improve the visualization of Alcian blue

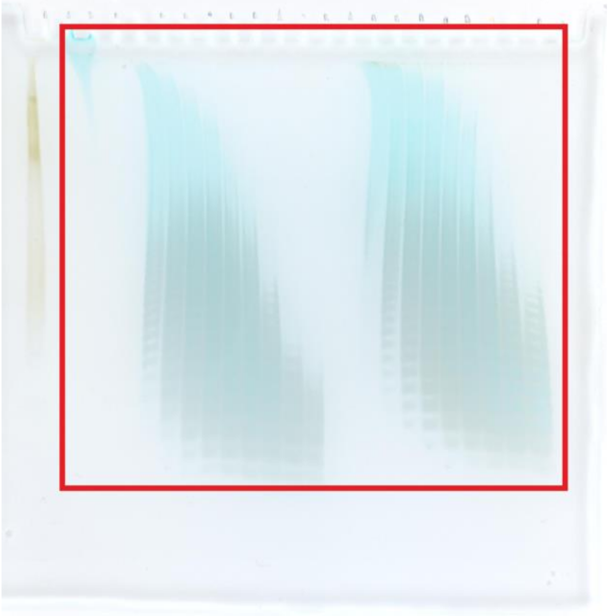

Extended Data Figure 7k
colors were adjusted equally across the entire image to improve the visualization of Alcian blue

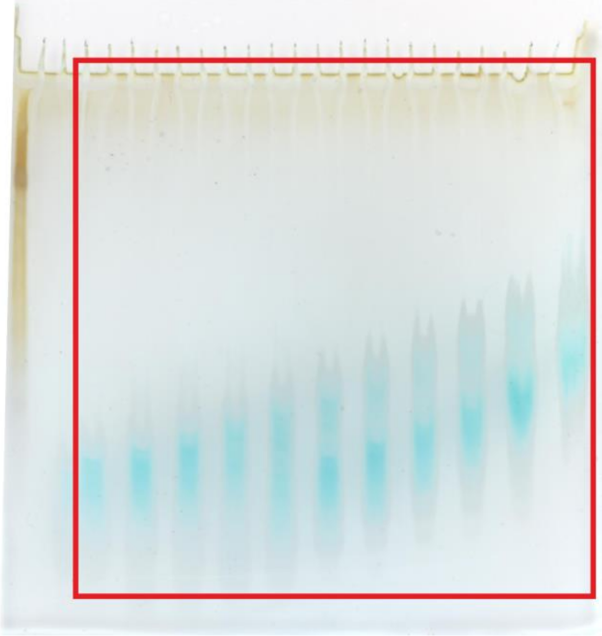

Supplement: Source Data Extended Data Fig. 7 — Unprocessed gels. [file 41589_2023_1324_MOESM9_ESM.pdf]
