# Supplementary material for: A multi-enzyme machine polymerizes the Haemophilus influenzae type b capsule
Source: Nat Chem Biol. 2023 Jun 5;19(7):865–77. doi: 10.1038/s41589-023-01324-3 (PMC10299916; doi:10.1038/s41589-023-01324-3)

Extended Data Figure 8a (red) and 8b (green)
colors were adjusted equally across the entire image to improve the visualization of Alcian blue

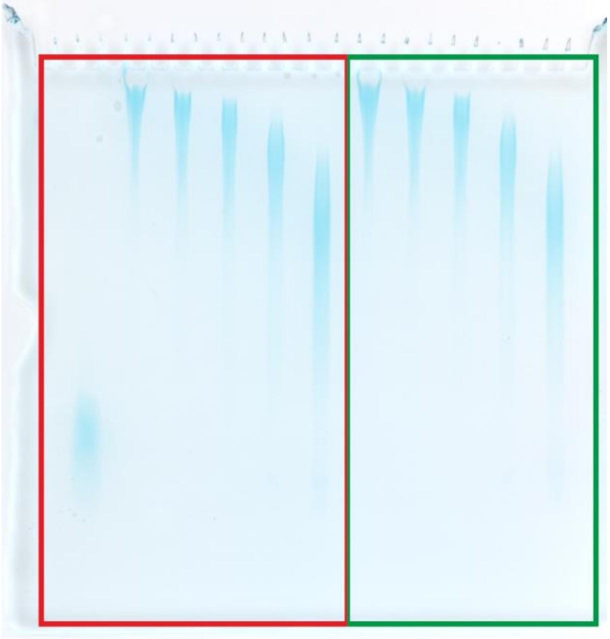

Extended Data Figure 8c
colors were adjusted equally across the entire image to improve the visualization of Alcian blue

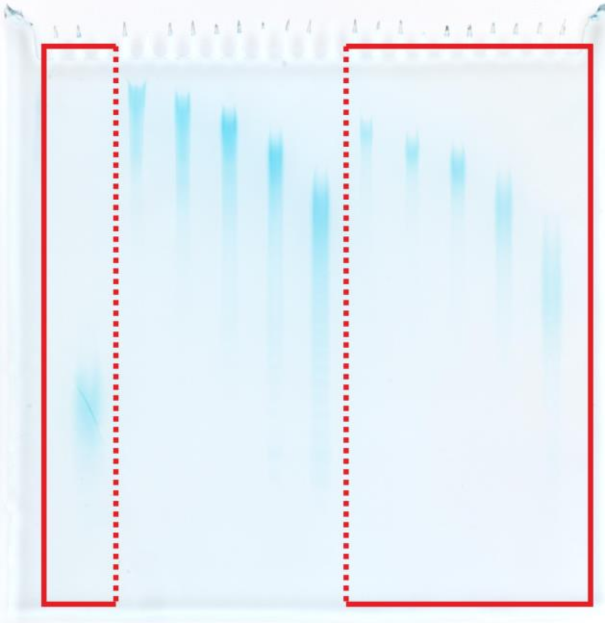

Extended Data Figure 8d
colors were adjusted equally across the entire image to improve the visualization of Alcian blue

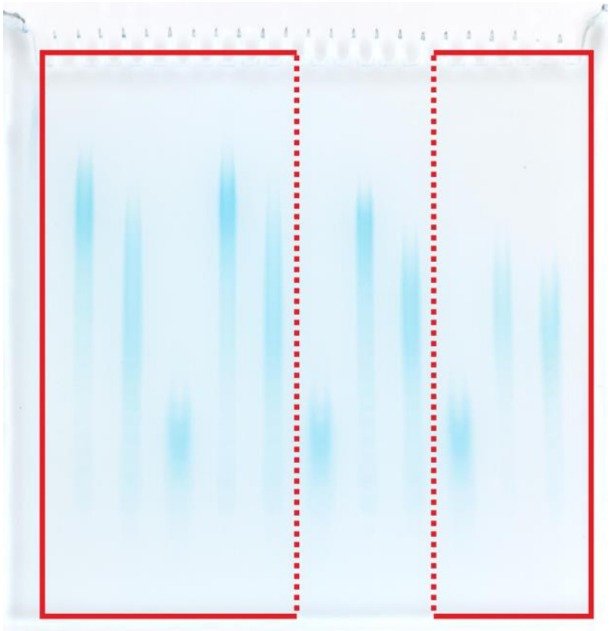

Supplement: Source Data Extended Data Fig. 8 — Unprocessed gels. [file 41589_2023_1324_MOESM10_ESM.pdf]
