# Supplementary material for: A multi-enzyme machine polymerizes the Haemophilus influenzae type b capsule
Source: Nat Chem Biol. 2023 Jun 5;19(7):865–77. doi: 10.1038/s41589-023-01324-3 (PMC10299916; doi:10.1038/s41589-023-01324-3)
Supplement: Supplementary file 4 — Sequence alignment of CroT homologs—Biochemically characterized CroT homolgoues from Gram-negative (gray) and Gram-positive (light gray) sources were aligned using MUSCLE72 and the alignment was annotated using Jalview71. Database references are provided. Homologs transferring ribitol-phosphate and glycerol-phosphate are indicated by full and empty circles, respectively9,73–76. The secondary structural elements extracted from a selected CriT from Bcs3-CMP are shown above the alignment. Amino acid residues with >70 % of identity are highlighted in black. A sequence logo plot is depicted below the alignment to provide a detailed overview of the general consensus at every position. Amino acids H96 and H233, which were targeted in this study by site-directed mutagenesis, are shown in red. Glycine and proline residues putatively determining the enzymes’ preference for ribitol and glycerol, respectively, are highlighted in yellow. [file 41589_2023_1324_MOESM4_ESM.pdf]

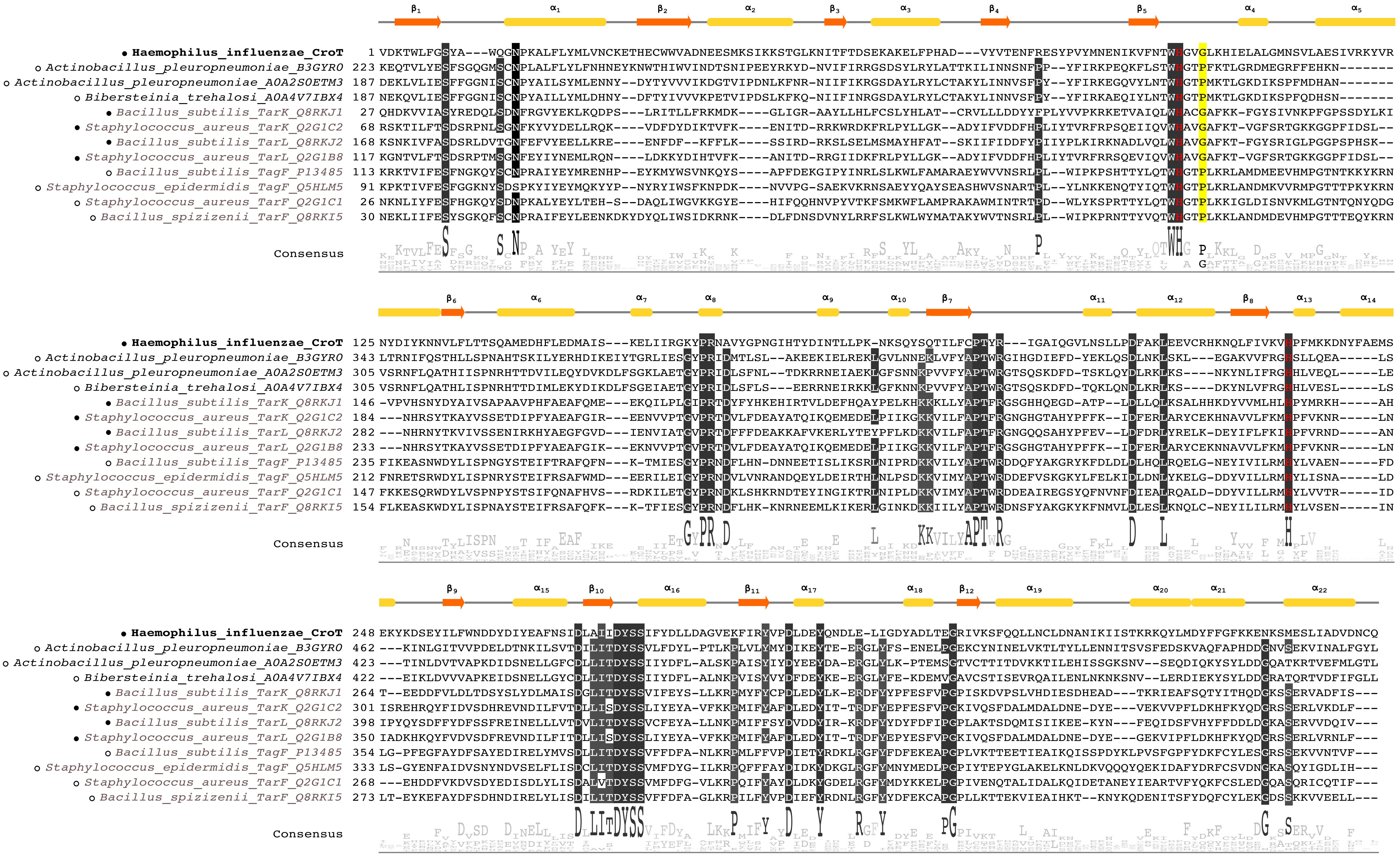
β1 α1 β2 α2 β3 α3 β4 β5 α4 α5
• Haemophilus_influenzae_CroT 1 VDKTWLFGSYA---WQGNPKALFLYMLVNCKETHECWWVADNEESMKSIKKSTGLKNITFTDSEKAKELFPHAD---VYVTENFRESYPVYMNENIKVFNTWHGVGLKHIELALGMNSVLAESIVRKYVR
o Actinobacillus_pleuropneumoniae_B3GYR0 223 KEQTVLYESFSGQGMSCNPLAFLYLFNHNEYKNWTHIWVINDTSNIPEEYRKYD-NVIFIRRGSDSYLRYLATTKILINNSNFPP--YFIRKPEQKFLSTWHGTPFKTLGRDMEGRFFEHKN-------
o Actinobacillus_pleuropneumoniae_A0A2S0ETM3 187 DEKLVLIESFFGGNISCNPYAILSYMLENNY--DYTYVVVIKDGTVIPDNLKFNR-NIIFIKRGSDAYLRYLCTAKYLINNVSFPY--YFIRKEGQVYLNTWHGTPMKTLGKDIKSPFMDHAN-------
o Bibersteinia_trehalosi_A0A4V7IBX4 187 NEKQVLIESFFGGNISCNPYAILLYMLDHNY--DFTYIVVVKPETVIPDSLKFKQ-NIIFINRGSDAYLRYLCTAKYLINNVSFPY--YFIRKAEQIYLNTWHGTPMKTLGKDIKSPFQDHSN-------
• Bacillus_subtilis_TarK_Q8RKJ1 27 QHDKVVIASYREDQLSDNFRGVYEKLKQDPS---LRITLLFRKMDK----GLIGR-AAYLLHLFCSLYHLAT--CRVLLLDDYYFPLYVVPKRKETVAIQLWHACGAFKK-FGYSIVNKPFGPSSDYLKI
• Staphylococcus_aureus_TarK_Q2G1C2 68 RSKTILFTSDSRPNLSGNFKYVYDELLRQK----VDFDYDIKTVFK----ENITD-RRKWRDKFRLPYLLGK--ADYIFVDDFHPLIYTVRFRPSQEIIQVWHAVGAFKT-VGFSRTGKKGGPFIDSL--
• Bacillus_subtilis_TarL_Q8RKJ2 168 KSNKIVFASDSRLDVTGNFEFVYEELLKRE----ENFDF--KFFLK----SSIRD-RKSLSELMSMAYHFAT--SKIIFIDDFYPIIYPLKIRKNADLVQLWHAVGAFKT-FGYSRIGLPGGPSPHSK--
• Staphylococcus_aureus_TarL_Q2G1B8 117 KGNTVLFTSDSRPTMSGNFEYIYNEMLRQN----LDKKYDIHTVFK----ANITD-RRGIIDKFRLPYLLGK--ADYIFVDDFHPLIYTVRFRRSQEVIQVWHAVGAFKT-VGFSRTGKKGGPFIDSL--
o Bacillus_subtilis_TagF_P13485 113 KRKTVIFESFNGKQYSCNPRAIYEYMRENHP-EYKMYWSVNKQYS----APFDEKGIPYINRLSLKWLFAMARAEYWVVNSRLPL--WIPKPSHTTYLQTWHGTPLKRLAMDMEEVHMPGTNTKKYKRN
o Staphylococcus_epidermidis_TagF_Q5HLM5 91 KPKTIVFESFGGKNYSDSPKYIYEYMQKYYP--NYRYIWSFKNPDK----NVVPG-SAEKVKRNSAEYYQAYSEASHWVSNARTPL--YLNKKENQTYIQTWHGTPLKRLANDMKVVRMPGTTTPKYKRN
o Staphylococcus_aureus_TarF_Q2G1C1 26 NKNLIYFESFHGKQYSDNPKALYEYLTEH-S--DAQLIWGVKKGYE----HIFQQHNVPYVTKFSMKWFLAMPRAKAWMINTRTPD--WLYKSPRTTYLQTWHGTPLKKIGLDISNVKMLGTNTQNYQDG
o Bacillus_spizizenii_TarF_Q8RKI5 30 NEKLIIFESYSGKQFSCNPRAIFEYLLEENKDYDYQLIWSIDKRNK----DLFDNSDVNYLRRFSLKWLWYMATAKYWVTNSRLPL--WIPKPRNTTYVQTWHGTPLKKLANDMDEVHMPGTTTEQYKRN
Consensus
β6 α6 α7 α8 α9 α10 β7 α11 α12 β8 α13 α14
• Haemophilus_influenzae_CroT 125 NYDIYKNNVLFLTTSQAMEDHFLEDMAIS---KELIIRGKYPRNAVYGPNGIHTYDINTLLPK-NKSQYSQTILFCPTYR---IGAIQGVLNSLLPDFAKLEEVCRHKNQLFIVKVHPFMKKDNYFAEMS
o Actinobacillus_pleuropneumoniae_B3GYR0 343 LTRNIFQSTHLLSPNAHTSKILYERHDIKEIYTGRLIESGPRIDMTLSL--AKEEKIELREKLGVLNNEKLVFYAPTWRGIHGDIEFD-YEKLQSDLNKLSKL---EGAKVVFRGHSLLQEA-----LS
o Actinobacillus_pleuropneumoniae_A0A2S0ETM3 305 VSRNFLQATHIISPNRHTTDVILEQYDVKDLFSGKLAETGYPRIDLSFNL--TDKRRNEIAEKLGFSNNKPVVFYAPTWRGTSQSKDFD-TSKLQYDLRKLKS----NKYNLIFRGHHLVEQL-----LE
o Bibersteinia_trehalosi_A0A4V7IBX4 305 VSRNFLQATHLISPNRHTTDIMLEKYDIKDLFSGEIAETGYPRIDLSFLS---EERRNEIRKKLGFKNNKPVVFYAPTWRGTSQSKDFD-TQKLQNDLKRLKS----DKYNLVFRGHHLVESL-----LS
• Bacillus_subtilis_TarK_Q8RKJ1 146 -VPVHSNYDYAIVSAPAAVPHFAEAFQME---EKQILPLGIPRTDYFYHKEHIRTVLDEFHQAYPELKHKKKLLYAPTFRGSGHHQEGD-ATP--LDLLQLKSALHHKDYVVMLHLHPYMRKH-----AH
• Staphylococcus_aureus_TarK_Q2G1C2 184 ---NHRSYTKAYVSSETDIPFYAEAFGIR---EENVVPTGVPRTDVLFDEAYATQIKQEMEDELPIIKGKKVILFAPTFRGNGHGTAHYPFFK--IDFERLARYCEKHNAVVLFKMHPFVKNR-----LN
• Bacillus_subtilis_TarL_Q8RKJ2 282 ---NHRNYTKVIVSSENIRKHYAEGFGVD---IENVIATGVPRTDFFFDEAKKAFVKERLYTEYPFLKDKKVILFAPTFRGNGQQSAHYPFEV--LDFDRLYRELK-DEYIFLFKIHPFVRND-----AN
• Staphylococcus_aureus_TarL_Q2G1B8 233 ---NHRSYTKAYVSSETDIPFYAEAFGIK---EKNVVPTGVPRTDVLFDEAYATQIKQEMEDELPIIKGKKVILFAPTFRGSGHGTAHYPFFK--IDFERLARYCEKNNAVVLFKMHPFVKNR-----LN
o Bacillus_subtilis_TagF_P13485 235 FIKEASNWDYLISPNGYSTEIFTRAFQFN---K-TMIESGYPRNDFLHN-DNNEETISLIKSRLNIPRDKKVILYAPTWRDDQFYAKGRYKFDLDLDLHQLRQELG-NEYIVILRMHYLVAEN-----FD
o Staphylococcus_epidermidis_TagF_Q5HLM5 212 FNRETSRWDYLISPNRYSTEIFRSAFWMD---EERILEIGYPRNDVLVNRANDQEYLDEIRTHLNLPSDKKVIMYAPTWRDDEFVSKGKYLFELKIDLDNLYKELG-DDYVILLRMHYLISNA-----LD
o Staphylococcus_aureus_TarF_Q2G1C1 147 FKKESQRWDYLVSPNPYSTSIFQNAFHVS---RDKILETGYPRNDKLSHKRNDTEYINGIKTRLNIPLDKKVIMYAPTWRDDEAIREGSYQFNVNFDIEALRQALD-DDYVILLRMHYLVVTR-----ID
o Bacillus_spizizenii_TarF_Q8RKI5 154 FLKEASKWDYLISPNAYSTEIFRSAFQFK---K-TFIESGYPRNDFLHK-KNRNEEMLKIKERLGINKDKKIILYAPTWRDNSFYAKGKYKFNMVLDLESLKNQLC-NEYILILRMHYLVSEN-----IN
Consensus
β9 α15 β10 α16 β11 α17 α18 β12 α19 α20 α21 α22
• Haemophilus_influenzae_CroT 248 EKYKDSEYILFWNDDYDIYEAFNSIDLAIIDYSSIFYDLLDAGVEKFIRYVPDLDEYQNDLE-LIGDYADLTEGRIVKSFQQLLNCLDNANIKIISTKRKQYLMDYFFGFKKENKSMESLIADVDNCQ
o Actinobacillus_pleuropneumoniae_B3GYR0 462 ---KINLGITVVPDELDTNKILSVTDILITDYSSVLFDYL-PTLKPLVLYMYDIKEYTE-ERGLYFS-ENELPGEKCYNINELVKTLTYLLENNITSVSFEDSKVAQFAPHDDGNVSEKVINALFGYL
o Actinobacillus_pleuropneumoniae_A0A2S0ETM3 423 ---TINLDVTVAPKDIDSNELLGFCDLLITDYSSIIYDFL-ALSKPAISYIYDFDEYDA-ERGLYLK-PTEMSGTVCTTITDVKKTILEHISSGKSNV--SEQDIQKYSYLDDGQATKRTVEFMLGTL
o Bibersteinia_trehalosi_A0A4V7IBX4 422 ---EIKLDVVVAPKEIDSNELLGYCDLLITDYSSIIYDFL-ALNKPVISYVYDFDEYKE-ERGLYFE-KDEMVGAVCSTISEVRQAILENLNKNKSNV--LERDIEKYSYLDDGRATQRTVDFIFGLL
• Bacillus_subtilis_TarK_Q8RKJ1 264 T--EEDDFVLDLTDSYSLYDLMAISDGLITDYSSVIFEYS-LLKRPMYFYCPDLEDYLK-ERDFYYPFESFVPGPISKDVPSLVHDIESDHEAD---TKRIEAFSQTYITHQDGKSSERVADFIS---
• Staphylococcus_aureus_TarK_Q2G1C2 301 ISREHRQYFIDVSDHREVNDILFVTDLLISDYSSLIYEYA-VFKKPMIFYAFDLEDYIT-TRDFYEPFESFVPGKIVQSFDALMDALDNE-DYE---VEKVVPFLDKHFKYQDGRSSERLVKDLF---
• Bacillus_subtilis_TarL_Q8RKJ2 398 IPYQYSDFFYDFSSFREINELLLVTDVLITDYSSVCFEYA-LLNKPMIFFSYDVDDYIR-KRDFYYDYFDFIPGPLAKTSDQMISIIKEE-KYN---FEQIDSFVHYFFDDLDGKASERVVDQIV---
• Staphylococcus_aureus_TarL_Q2G1B8 350 IADKHKQYFVDVSDFREVNDILFITDLLISDYSSLIYEYA-VFKKPMIFYAFDLEDYIT-TRDFYEPYESFVPGKIVQSFDALMDALDNE-DYE---GEKVIPFLDKHFKYQDGRSSERLVRNLF---
o Bacillus_subtilis_TagF_P13485 354 LG-PFEGFAYDFSAYEDIRELYMVSDLLITDYSSVFFDFA-NLKRPMLFFVPDIETYRDKLRGFYFDFEKEAPGPLVKTTEETIEAIKQISSPDYKLPVSFGPFYDKFCYLESGRSSEKVVNTVF---
o Staphylococcus_epidermidis_TagF_Q5HLM5 333 LS-GYENFAIDVSNYNDVSELFLISDCLITDYSSVMFDYG-ILKRPQFFAYDIDKYDKGLRGFYMNYMEDLPGPIYTEPYGLAKELKNLDKVQQQYQEKIDAFYDRFCSVDNGKASQYIGDLIH---
o Staphylococcus_aureus_TarF_Q2G1C1 268 ---EHDDFVKDVSDYEDISDLYLISDALVTDYSSVMFDFG-VLKRPQIFYAYDLDKYGDELRGFYMDYKKELPGPIVENQTALIDALKQIDETANEYIEARTVFYQKFCSLEDGQASQRICQTIF---
o Bacillus_spizizenii_TarF_Q8RKI5 273 LT-EYKEFAYDFSDHNDIRELYLISDLLITDYSSVFFDFA-GLKRPLIFYVPDIEFYRDNLRGFYYDFEKCAPGPLLKTTEKVIEAIHKT--KNYKQDENITSFYDQFCYLEKGDSSKKVVEELL---
Consensus
